# Study of interaction of metal ions with methylthymol blue by chemometrics and quantum chemical calculations

**DOI:** 10.1038/s41598-021-85940-w

**Published:** 2021-03-19

**Authors:** Zolaikha Rasouli, Mehdi Irani, Sonia Jafari, Raouf Ghavami

**Affiliations:** 1 Chemometrics Laboratory, Chemistry Department, Faculty of Science, University of Kurdistan, P.O. Box 416, 66177-15175 Sanandaj, Iran; 2 Theoretical Chemistry Laboratory, Chemistry Department, Faculty of Science, University of Kurdistan, P.O. Box 416, 66177-15175 Sanandaj, Iran

**Keywords:** Analytical chemistry, Theoretical chemistry, Chemistry, Physical chemistry, Spectrophotometry

## Abstract

In this study, we determine the acidity constants of methylthymol blue (MTB) and association constants of its complexes with the Zn^II^, Cu^II^, and Fe^II^ metal ions (MIs), through theoretical and experimental means. The complexes were characterized using UV–Visible absorption spectroscopy combined with soft/hard chemometrics methods and quantum chemical calculations. Quantum chemical calculations revealed that electronic transitions in the UV–Visible spectra of MTB have mixed n → π* and π → π* characters. The results of molar ratio and multivariate curve resolution alternating least squares (MCR-ALS) revealed the formation of successive 1:2 and 1:1 complexes (MI:MTB) for the Zn^II^ and Cu^II^ systems. However, the formation of successive 1:1 and 2:1 complexes are suggested for Fe^II^ by the molar ratio and MCR-ALS. The majority of transitions observed in the UV–Visible spectra of the Zn(MTB) and Cu(MTB) complexes have ligand-to-ligand charge transfer (LLCT) characters. However, the transitions in the UV–Visible spectrum of the Fe(MTB) complex have LLCT and metal-to-ligand charge transfer (MLCT) characters. For the Fe_2_(MTB) complex, the lowest energy transition of has an LLCT character. However, its higher energy transitions are a mixture of LLCT, MLCT, and metal-to-metal charge transfer (MMCT) characters. The correlation between experimental and computed wavelengths revealed that the 1:1 complexes of Zn^II^ and Cu^II^ prefer square pyramidal geometries. However, the Fe^II^ complexes always show octahedral geometry.

## Introduction

Metallochromic indicators are broadly employed in chemical analysis^1,2^. The metallochromic indicators are used as equivalence point identifiers in chelometric titrations, as spectrophotometric reagents to form stable complexes in spectrophotometric/fluorimetric quantification of MIs and as preconcentration agents in separation of trace quantities of heavy MIs^3–6^. Furthermore, they are employed as active modifiers on different nanocomposites and liquid membrane electrodes for measuring MIs^7–13^. MI-metallochromic indicator complexes can form organometallic dyes, which have different colors and distinguished spectral features from that of the intact metallochromic indicators^14^. Physiochemical knowledge about MI-metallochromic indicator complexes can provide a deep insight into the determination of active chemical contributions of each chemical species at their complexation systems, coordination sites, coordination numbers and molecular geometries of MI-metallochromic indicator complexes. These are necessary for some applications e.g. in measuring acidic or association constants or when applying methods like the ion-increment procedure for the determination of free MI concentrations.

**Figure 1:**
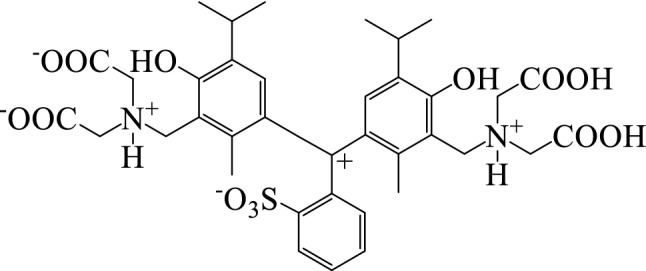
Molecular structure for the 6-protonated form of MTB.

Here, we considered 3,3′-Bis[N,N-di(carboxymethyl)aminomethyl]thymolsulfonephthalein (Methylthymol blue; MTB) as a representative of thymol containing metallochromic indicators. The MTB has nine active functional groups (four carboxylic acids, two phenols, two amines and one sulfonyl (cf. Fig. 1 for MTB structure). This indicator is similar to xylene orange and has an N_2_O_6_ coordination sphere geometry. These groups give it a capacity of complexation with various bivalent MIs. MTB is a sensitive but non-selective metallochromic indicator, and interacts with more than twenty MIs, depending on the pH value of solution^15–18^. It has been used as a chelator for iron in ferrous/Fricke gel dosimeters for 3-dimensional dosimetry in cancer radiotherapy^19,20^.

However, despite the widespread use of MTB, little is known regarding the complexation properties of MTB and MIs. Shimada et al. investigated pH-dependent chemical equilibria of bromothymol blue (BTB) and thymol blue (TB) using absorption spectra and theoretical calculations^21,22^. Both BTB and TB molecules are from thymol containing metallochromic indicator. The studies of Shimada et al. have been done in absence of any MI. Compared to the MTB, the BTB and TB are smaller molecules and have very less active functional groups, whereas the MTB is a large molecule with a large number of active functional groups.

In general, multivariate spectrophotometry techniques because of their high sensitivity are convenient methods for exploring chemical equilibria of MI-metallochromic indicator complexes^23^. Nevertheless, such studies usually lead to overlapping spectral responses. This implies that chemical equilibria analysis of MI-metallochromic indicator complexes is not straightforward using ordinary non-based factor methods. Hence, use of chemometrics calculations is the best candidate in such conditions (especially in ill conditions)^24^. In most cases, chemometrics methods such as soft modeling (e.g. MCR-ALS) do not require previous chemical knowledge of the under-study chemical system^25,26^.

Methods originated from the quantum chemical such as density functional theory (DFT) and its time-dependent form (TD-DFT) have appeared as reliable methodologies in computational chemistry^27,28^. The DFT approaches have widely been used to study different aspects of chemical studies, such as investigating electronic structures and reaction mechanisms of some small molecules^29,30^, adsorption properties of surface reactions^31^ and reaction mechanism of complicated enzymatic systems^32^. In addition, there is a large number of previous studies that used TD-DFT calculations to predict electronic transitions and to establish a correlation between the experimental and computed wavelengths^33–38^. In other words, the reliability of DFT approaches to simulate UV–Visible spectra of organic and organometallic molecules are well documented.

Our study aims to gain insights into the Zn^II^-MTB, Cu^II^-MTB and Fe^II^-MTB complexation systems in aqueous solution. In order to attain this purpose, we use UV–Visible spectroscopy combined with chemometrics and TD-DFT calculations. This paper is organized as follows. We first introduce the experimental and theoretical methods employed in this study. Then, we provide an exploratory study of the free MTB by UV–Visible spectroscopy. The acidity constants and resulting, the pure concentration/absorption profiles of the free MTB species are determined by the Marquardt–Levenberg algorithm (MLA). More, the TD-DFT calculations used for theoretical characterize a few cases of protonated/deprotonated forms of free MTB. In the following, we provide an exploratory study of the complexation MTB with Zn^II^, Cu^II^, and Fe^II^ by UV–Visible spectroscopy. The stoichiometries and the pure concentration/absorption profiles are determined by MCR-ALS. The association constants of complexes are determined by rank annihilation factor analysis (RAFA). The TD-DFT calculations were employed to identify the nature of the molecular geometries of complexes. This carries out by using TD-DFT optimization for hypothetical molecular structures (HMSs) and finally, computation of their electronic transitions to make a comparison between the experimental and computed wavelengths.

## Experimental and theoretical methods

### Materials and reagents

All chemicals were of analytical reagent grade (Sigma-Aldrich and Merck). The 0.1 M stock aqueous solutions of Zn^II^, Cu^II^, and Fe^II^ were prepared by dissolving Zn(NO_3_)_2_·6H_2_O, Cu(NO_3_)_2_·6H_2_O, and Fe(NO_3_)_2_ salts in doubly distilled-deionized water, respectively. To prevent oxidation of Fe^II^ to Fe^III^, the complexation procedure for the Fe^II^ was performed in the presence of a hydroxylamine hydrochloride solution (NH_2_OH·HCl 0.1 M). A 0.1 M solution of MTB was prepared by dissolving it in doubly distilled-deionized water. A 10 mM solution of a borax (Na_2_B_4_O_7_·10H_2_O) buffer at pH 5 was prepared by titration of borax solution with HCl. The ionic strength of all working solutions was preserved at 0.1 M NaCl (T = 25 ± 1 °C).

### Instruments

The absorbance measurements were performed on an Analytic Jena Specord E250 spectrophotometer (Germany, www.analytik-jena.de) equipped with a thermostat Lauda Ecoline Staredition RE 104 and 1-cm quartz cells. The pH measurements were carried out using Metrohm 713 model pH-meter (with precision of ± 1 mV) furnished with a combined glass-saturated calomel electrode.

### Experimental methods

Spectrophotometric-pH metric titration of MTB was performed by titration of a 50 mL of 1 mM aqueous solution of MTB at ionic strength of 0.1 M NaCl and T = 25 ± 1 °C with either concentrated HCl or NaOH solutions. Before the addition of each portion of the titrants (NaOH or HCl), the solution was permitted to equilibrate until pH drift < 0.01 unit/2 min was attained. The pH changes per titrant addition were limited to 0.25–0.5 pH units. A total number of 36 pH points in the range of 1.25–13 was recorded. In each pH, the absorption spectra were measured over the wavelength range of 240–750 nm with 1 nm intervals. The data were organized to a set of 36 × 511 dimension.

Before performing the complexation process, the stability of the MTB solution was tested. We first prepared an MTB solution and then, recorded its UV–Visible spectrum. After that, the solution was left for one hour and then, its spectrum was recorded again. Figure S1 in the Supplementary Information shows the absorption spectra and digital photographs of the MTB solution for 1 h. As can be seen, the shape and partly intensity of the absorption spectra of the MTB solution had not changed after 1 h. This result is reflected in the lack of color change of MTB solution after one hour.

The complexation procedure was performed according to the molar ratio method. For each of the complexation systems, 2.5 mL of borax buffer solution (pH 5) was transferred to 5 mL volumetric flasks. This was followed with the addition of appropriate aliquots of Zn^II^, Cu^II^, and Fe^II^ standard solutions, 100 μL of MTB 1 mM and 50 μL of 0.1 M NH_2_OH.HCl solutions (the latter was added only to Fe^II^ solutions). The concentration of each MI ranged from 0.00 to 0.06 mM up to molar ratio (*R*) ~ 3. The *R* is defined as [cation]/[MTB]. The solutions were diluted to the mark with doubly distilled-deionized water. No precipitation was observed for any of the solutions. A few previous studies^39,40^ and preliminary tests showed that the complexation reactions of MTB with MIs are relatively fast (it takes 10 min to ensure the complexation systems to equilibrate). Thus, the absorption spectrum of each solution was measured after 10 min of preparation. For each of the complexation systems, a data set with a 75 × 511 dimension was obtained. It is worthwhile to note that no side reaction among the borax buffer components and the studied MIs have been reported^41–46^. Whereas, the acetate buffer forms complexes with the Cu^II^ and Zn^II^ ions^47^ and the phosphate buffer forms complexes with the Zn^II^ ion^48^. Meanwhile, the UV–Visible spectrum of MTB is independent of the buffer type. These were the main reasons for the selection of borax buffer in this study.

## Chemometrics data processing methods

### MLA

To refine the acidity constants of MTB, we used a computer program (m-file) which was improved based on the MLA in MATLAB (version R2010a, https://www.mathworks.com) environment. Then, the program was used to calculate the pure concentration and spectral profiles of protonated/deprotonated forms of MTB during the acid–base titration. Details of MLA theory and the basis of the written m-file can be found in the Supplementary Information.

### MCR-ALS soft modeling

The Toolbox MCR-ALS GUI 2.0^49^ program was employed to extract pure concentrations and spectral profiles of active chemical species in each of the complexation systems. Theoretical details of the MCR-ALS modeling are summarized in the Supplementary Information. Here, to conduct the MCR-ALS analysis, initial approximations of all component concentrations were determined by evolving factor analysis. The non-negativity in concentration and spectral directions, unimodality in concentration direction and normalization of spectra constraints were applied during the optimization of alternative least square. The convergence value (0.1) was completed at less than 50 iterations.

### RAFA hard-soft modeling

Two m-files based on mass balance rules governing the proposed mechanisms for the complexation systems were written in MATLAB (version R2010a) environment. The m-files were used to extract the association constants of complexes. Details of RAFA theory and the basis of written m-files can be found in the Supplementary Information.

### Details of the DFT calculations

All DFT calculations were performed using the hybrid density functional B3LYP^50^, implemented in the Gaussian09 package program^51^. The reason for using the B3LYP functional was its ability to compute of absorption wavelengths in the UV–Visible domain^52^. The Lanl2dz basis set and pseudo potential^53^ was used for the MIs whereas the 6-31G basis set was used for the other atoms. For computations involving the copper ion, the unrestricted formalism of DFT was used, whereas the restricted one was used for computations of the other systems. Geometry optimizations were carried out using Berny algorithm^54^ without any constraints in the geometrical structures. TD-DFT formalism in the adiabatic approximation was used to compute excitation wavelengths and oscillators strengths.

TD-DFT methods have been shown to give accuracy comparable to post-Hartree–Fock methods with a suitable functional^55^, it is therefore often regarded as a good compromise between accuracy and CPU time for metal complexes^56^. The 100 lowest excited states were taken into account to compute vertical excitation energies. The solvent (water) was represented by explicit molecules added in the coordination sphere of MIs (the number of water molecules is determined by examination of the release of molecules during the optimization) and by the addition around this complex of an implicit solvent using the conductor like polarized continuum (CPCM) model^57^ as implemented in Gaussian. The CPCM calculations used UFF atomic rad^II^ and default water solvent parameters in the Gaussian09 package. The explicit addition of water molecules is necessary to take into account the fact that solvent molecules around MIs act both as a solvent and as a ligand. This addition leads to a good reproduction of the experimental data that a purely implicit solvent model cannot achieve and also eliminates the transitions computed at very low energies^58^. All geometrical structures were optimized before TD-DFT calculations. All geometrical structures were optimized before TD-DFT calculations. The percentage contributions of excitations were extracted from the TD-DFT output files using the Gauss Sum program^59^ (It is also possible to extract the spectral futures directly from the Gaussian log file. However, the Gausssum program makes the extraction task much convenient and faster).

## Results and discussion

### Free MTB

**Figure 2:**
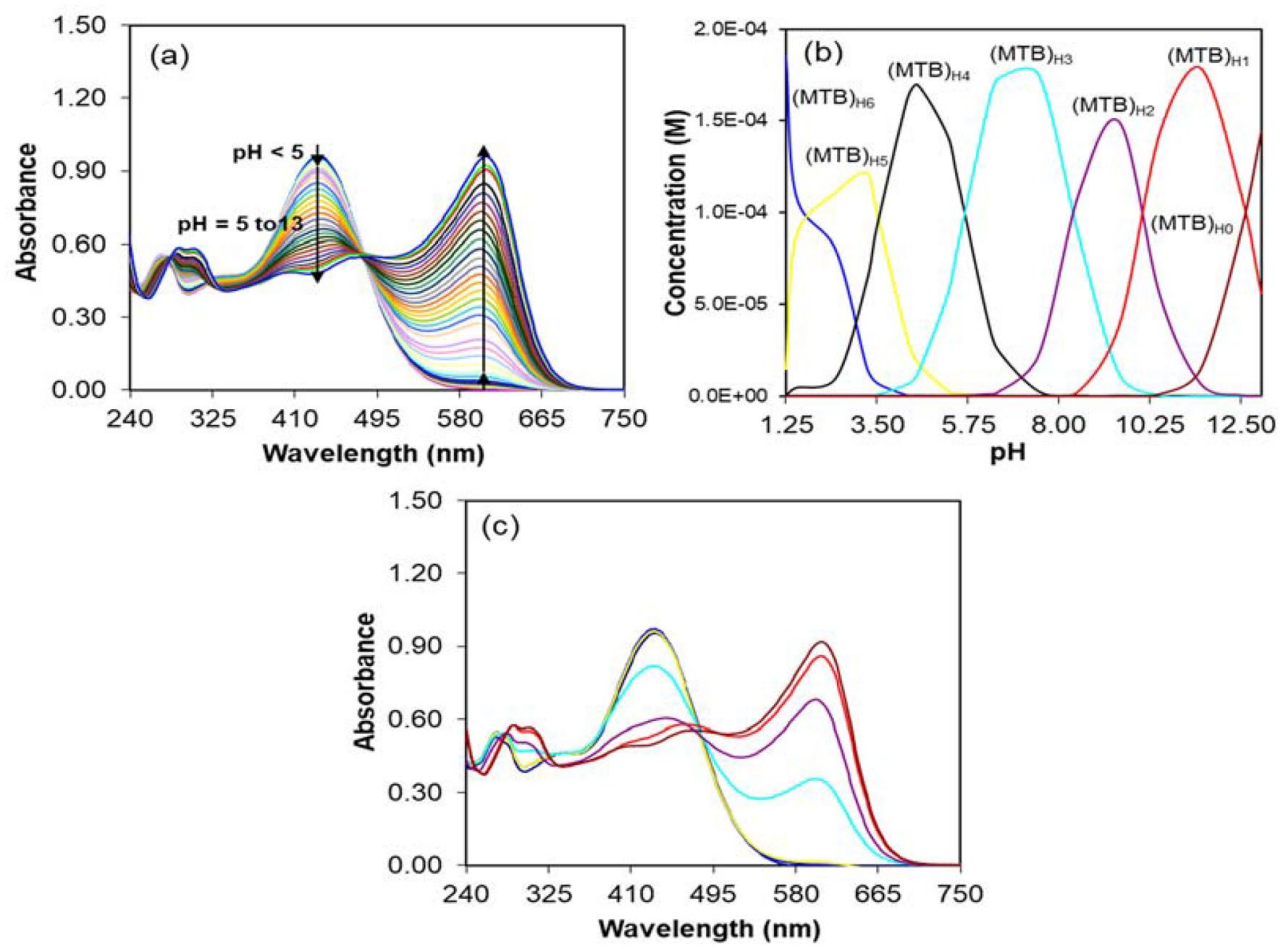
(**a**) UV–Visible spectra of MTB during the acid–base titration in the range of pH 1.25–13, (**b**) concentration profiles, and (**c**) spectral profiles resolved by the MLA.

We first centralized our attention on the acid–base absorption properties of MTB. According to Fig. 2a, for pH < 5, the UV–Visible spectrum of MTB can be described by a strong band at 440 nm and a relatively weak band at 277 nm. The bands at 440 and 277 nm are separated by a moderately intense shoulder at 355 nm. As can be seen, by increasing pH, maximum peak intensity decreases and simultaneously emerges a strong band at 606 nm and a relatively strong band around 310 nm. The band at 310 nm accompanied with a moderately intense shoulder is distinguishable around 400 nm (cf. Fig. 2a). These bands can be assigned to the different deprotonated forms of MTB. Fully deprotonation of MTB increases the negative charge on the molecule. This in turn causes a red shift in the absorption spectrum of MTB. Since MTB has nine functional groups, then it’s fully protonated form can be shown as MTB_H9_. However, previous potentiometric studies on the acid–base behavior of MTB revealed that it undergoes a 6-step acidic dissociation and thus, it can be considered as a 6-protic acid. This is in agreement with reported small p*K*_*a*_ values for the sulfonyl and two carboxylic acids functional groups. In conclusion, these functional groups are completely dissociated in water^60,61^. Figure S2 in the Supplementary Information shows different protonated forms of MTB (MTB_H6_, MTB_H5_, MTB_H4_, MTB_H3_, MTB_H2_, MTB_H1_ and MTB_H0_). Fitting of the titration data set of MTB using the MLA by the correct model and approximate initial guesses of the acidity constants results in the acidity constants listed in Table 1. As can be seen, all the acidity constants are well defined with the residual error (**σ**_r_) = 0.0086 and standard errors (**σ**_P_) < 0.15, respectively. In addition, there is a good agreement between the computed and the previously reported acidity constants values (compare the data in the second and third columns in Table 1)^62^. It should be noted that it is not possible to measure the p*K*_a1_, p*K*_a2_, and p*K*_a3_ values by the presented spectrophotometry method. Figure 2b,c shows the resolved concentration and spectral profiles by the MLA. It can be seen that the concentration and spectral profiles of all components are well resolved. The resolved spectral profiles of MTB_H6_, MTB_H5_, and MTB_H4_ are very similar in their spectral features. These suggest that the MLA is capable to resolute chemical species with very similar spectral features. At MLA the concentration matrix is expressed as a function of the acidity constants (non-linear parameters). This effectively eliminates the linear dependency in the spectral matrix.

**Table 1:** The computed p*K*_a_ values of MTB by the MLA and those reported previously using stability quotients from absorbance data (SQUAD) program^62^.

p*K*_a_	MLA	SQUAD
p*K*_a1_	–	–
p*K*_a2_	–	–
p*K*_a3_	–	–
p*K*_a4_	2.35 (± 0.048)	–
p*K*_a5_	3.54 (± 0.039)	3.47 (± 0.058)
p*K*_a6_	5.66 (± 0.089)	6.31 (± 0.081)
p*K*_a7_	8.39 (± 0.065)	8.47 (± 0.081)
p*K*_a8_	10.08 (± 0.113)	10.81 (± 0.073)
p*K*_a9_	12.59 (± 0.142)	12.97 (± 0.045)

Numbers in parentheses indicate the standard error (**σ**_P_) values on acidity constants.

**Figure 3:**
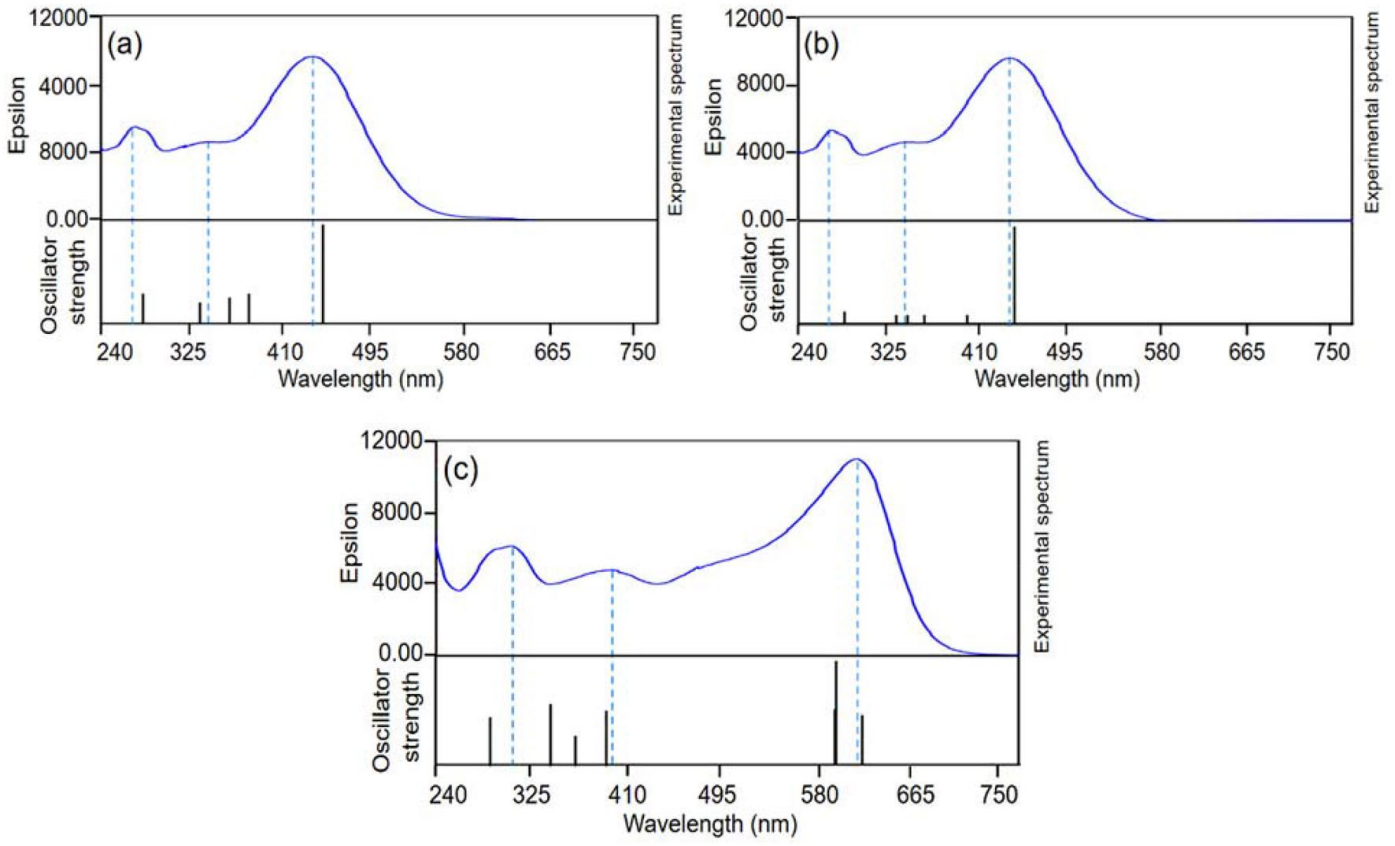
Computed wavelengths (black) and experimental spectrum (blue) for: (**a**) MTB_H4_, (**b**) MTB_H6_ and (**c**) MTB_H0_.

In the DFT/TD-DFT study of the free MTB, we focused on the MTB_H4_ (the predominant form MTB at pH 5), MTB_H6_ (fully protonated MTB) and MTB_H0_ (fully deprotonated MTB) forms. The computed wavelengths and the experimental spectra of the MTB_H4,_ MTB_H6_ and MTB_H0_ are illustrated in Fig. 3. Details of the computed and experimental wavelengths are summarized in Table 2. For MTB_H4_, the computed wavelengths at 451 nm (*f* = 0.43; the letter *f* refers to the computed oscillator strength) and 284 nm (*f* = 0.13) reproduce well the experimental absorption bands at 440 nm and 277 nm. In addition, we computed molecular orbitals (MOs), their energies and the contributions of inter MO transitions in the computed wavelengths for MTB_H4_. MO analysis shows that Ho-2 → Lu and Ho → Lu + 2 transitions have contributed 77% and 71% in the bands of 451 and 284 nm (Ho and Lu denote the HOMO and LUMO orbitals, respectively; Ho − *i* and Lu + *i* show the *i*th orbital below Ho and above Lu, respectively). However, some transitions (Ho − 7 → Lu (51%), Ho − 11 → Lu (41%), Ho − 13 → Lu (13%) and Ho − 15 → Lu (59%); cf. Table 2) contributes to the shoulder observed at 355 nm. Indeed, the computed absorption spectrum for MTB_H4_ is in good agreement with the experimental one (cf. Fig. 3a).

**Table 2:** Experimental and computed wavelengths, MO contributions (≥ %10), and oscillator strength (*f*) involved in each transition for MTB_H4_, MTB_H6_, and MTB_H0_. Phrase sh stands for shoulder.

	Exp	Com		
	λ (nm, ev)	Λ (nm, ev)	Main transitions	*f*
MTB_H4_	440 (2.82)	451 (2.74)	Ho − 2 → Lu (77%), Ho − 3 → Lu (10%)	0.43
	355 (3.49, sh)	382 (3.25)	Ho − 7 → Lu (51%), Ho − 12 → Lu (18)	0.13
		364 (3.40)	Ho − 11 → Lu (41%), Ho − 12 → Lu (14%), Ho − 13 → Lu (22%)	0.11
		339 (3.66)	Ho − 15 → Lu (59%),	0.09
	277 (4.48)	284 (4.37)	Ho → Lu + 2 (71%), Ho − 23 → Lu (11%)	0.13
MTB_H6_	440 (2.82)	443 (2.80)	Ho → Lu (89%)	0.47
		400 (3.10)	Ho − 2 → Lu (81%)	0.05
	355 (3.49, sh)	363 (3.41)	Ho − 7 → Lu (84%)	0.05
		346 (3.58)	Ho − 8 → L (34%), Ho − 9 → Lu (16%), Ho − 11 → Lu (38%)	0.05
		343 (3.61)	Ho − 8 → Lu (13%), Ho − 9 → Lu (69%)	0.05
	277 (4.48)	286 (4.33)	Ho → Lu + 1 (79%), Ho → uL + 2 (13%)	0.06
MTB_H0_	606 (2.03)	609 (2.04)	Ho − 1 → Lu (68%), Ho → Lu (32%)	0.13
		587 (2.11)	Ho → Lu (51%), Ho − 1 → Lu (25%), Ho − 2 → Lu (23%)	0.30
		585 (2.12)	Ho − 2 → Lu (75%), Ho → Lu (17%)	0.14
	396 (3.13)	393 (3.15)	Ho − 11 → Lu (42%), Ho − 18 → Lu (25%)	0.14
		369 (3.36)	Ho → Lu (68%), Ho − 18 → Lu (15%)	0.09
		347 (3.57)	Ho → Lu + 2 (92%)	0.18
	310 (4.00)	295 (4.20)	Ho → Lu + 4 (91%)	0.14

The DFT/TD-DFT calculations for MTB_H6_, reproduce well the lowest energy transition (LET) at 440 nm (the computed LET and the corresponding oscillator strength are 443 nm and 0.47, respectively). The main contributor to this transition is Ho → Lu (89%). This transition is flanked by a transition with a low intensity at 400 nm (*f* = 0.05). The band at 277 nm is also well reproduced with a transition at 286 nm (*f* = 0.06 and the main contributor is Ho → Lu + 1 with 79% contribution). The shoulder located at 355 nm is produced by some transitions with low oscillator strength (cf. Fig. 3b). The computed LET for the MTB_H0_ appeared as multi-peaks around 600 nm (i.e. 606, 587, and 585 nm). Where the transitions at shorter wavelengths are reproduced more accurately e.g. those in 393 and 295 nm (cf. Fig. 3c).

A few frontier MOs of the MTB_H4_, MTB_H6,_ and MTB_H0_ forms are illustrated in Fig. S3. Visual investigation of the 3D images of the MOs imply that the detected electronic transitions in the UV–Visible spectra of MTB_H4_, MTB_H6_ and MTB_H0_ have mixed n → π* and π → π* characters. We can see significant topological differences for the MOs of the MTB_H4_ and MTB_H6_. For MTB_H6_, in the two highest occupied MOs (Ho and Ho-2), the electronic density is localized only over the sulfonic moiety with some minor contribution of a π-system. Wherever, for MTB_H4_, Ho and Ho-2 are essentially localized on the carboxylate moieties with some minor contribution from one of the nitrogen atoms.

### Complexation of MTB with Zn^II^, Cu^II^ and Fe^II^

#### Spectral properties

**Figure 4:**
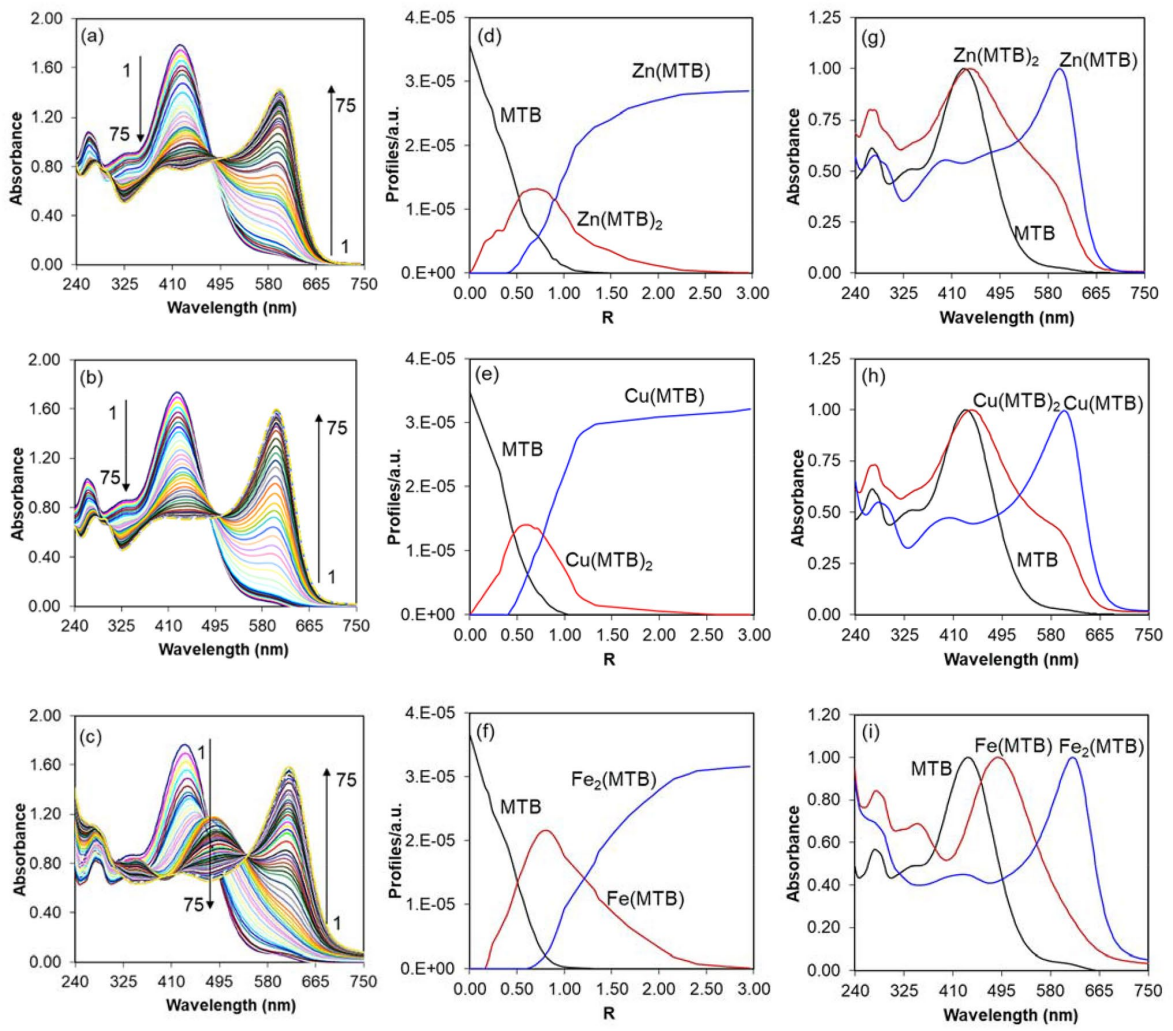
UV–Visible spectra of the complexation systems of MTB with (**a**) Zn^II^, (**b**) Cu^II^ and (**c**) Fe^II^, the concentration (**d**–**f**) and spectral (**g**–**i**) profiles obtained using MCR-ALS. The numbers 1 and 75 corresponds to 0 and 2.96 *R* values, respectively. The LOF values were below 1%.

Figure 4a–c displays the illustrative spectrophotometric complexation of the Zn^II^, Cu^II^, and Fe^II^ ions with MTB. In this section and the following sections, MTB refers to its predominant form at pH 5 (i.e. MTB_H4_). For all three complexation systems, we see bathochromic shifts in the absorption maxima peak of MTB to longer wavelengths. For the Zn^II^ and Cu^II^ complexation systems, the decrease in the peak intensity at 440 nm accompanied by a progressive shift and subsequently an emergence of a new peak in the range of 500–700 nm. However, the complexation for Fe^II^ starts with an emergence of very strong peaks at 400–550 and 550–700 nm. Plots extracted from the molar ratio method propose formation of successive Zn(MTB)_2_ and Zn(MTB) complexes for Zn^II^, Cu(MTB)_2_ and Cu(MTB) complexes for Cu^II^, and Fe(MTB) and Fe_2_(MTB) complexes for Fe^II^.

Next, we used the MCR-ALS model to estimate molar ratios that the chemical species emerge, destroy and or stabilize. Figure 4d–f and g–I show the pure concentration and spectral profiles are obtained by the MCR-ALS model. The three components detected for each of the complexation systems are sufficient to represent the majority information of the input data sets (explained variances > 97%). All the percentage lack of fit (LOF) values were below 1%. Addition of Zn^II^/or Cu^II^ to the reaction solution accompanied by a constant decrease at the concentration profile of MTB. This decrease is due to the formation of MI(MTB)_2_ and MI(MTB) complexes in succession. The complexes of Zn(MTB)_2_ and Cu(MTB)_2_ emerge from the beginning of the complexation reactions, reach their maxima at the *R* value of ~ 0.57, stabilize and then disappear. The resolved profiles (Fig. 4d–f) show the formation of Zn(MTB)_2_ and Cu(MTB)_2_ complexes. Addition of more Zn^II^ or Cu^II^ solutions decrease Zn(MTB)_2_ or Cu(MTB)_2_ concentration and consequently, form complexes of Zn(MTB) and Cu(MTB). However, adding more Fe^II^ solution, first forms complexes of Fe(MTB) and then Fe_2_(MTB). The resolved spectral profiles of all complexes are shown in Fig. 4g–i. The maxima peak for the Zn(MTB), Cu(MTB), and Fe_2_(MTB) complexes appear almost at the same location (600 nm for Zn^II^/or Cu^II^ and 622 nm for Fe^II^). Furthermore, the absorption spectrum for Zn(MTB) is accompanied by an extra shoulder at 490 but Cu^II^ and Fe^II^ do not show these shoulders.

Finally, we employed the RAFA model to estimate association constants of the complexes. At RAFA model, the lowest value in the residual standard deviation (R.S.D.) plots is detected, after removing the contribution of a chemical species (here, MTB) from the data set to reduce the rank by one. These R.S.D.s show the optimum values of association constants. The R.S.D. surfaces are shown in Fig. S4 in the Supplementary Information. The values of the calculated association constants are listed in Table 3. All the association constants are well defined with standard deviations values < 0.06. As is clear in Table 3, the stability order of the Zn^II^ and Cu^II^ complexes follow the Irving-Williams order (i.e. Fe and Cu complexes are more stable than the Zn complexes)^63^. The Zn^II^ complexes have the lowest stability and this is in agreement with the fact that their crystal field stabilization energy is zero^63^. However, the Fe^II^ complexes considerably deviate from the Irving-Williams series. One of the main reasons for this deviation is that the Fe^II^ complexes exhibit different stoichiometry than those of Cu^II^ or Zn^II^. Thus, other factors such as entropy and steric effects may play a key role in determining the association constants of Fe^II^ with MTB.

**Table 3:** The stoichiometry and association constants of MTB complexes with Zn^II^, Cu^II^, and Fe^II^ at the aqueous solution (pH 5).

MIs	Stoichiometry	Association constants	
Zn^II^	Zn(MTB)_2_	Log *K*_*a1*_	4.45 (± 0.03)
	Zn(MTB)	Log *K*_*a2*_	3.18 (± 0.05)
Cu^II^	Cu(MTB)_2_	Log *K*_*a1*_	5.64 (± 0.06)
	Cu(MTB)	Log *K*_*a2*_	3.49 (± 0.02)
Fe^II^	Fe(MTB)	Log *K*_*a1*_	6.96 (± 0.04)
	Fe_2_(MTB)	Log *K*_*a2*_	4.81 (± 0.05)

Numbers in parentheses indicate the standard deviation values (n = 3).

#### DFT study of the complexes

**Figure 5:**
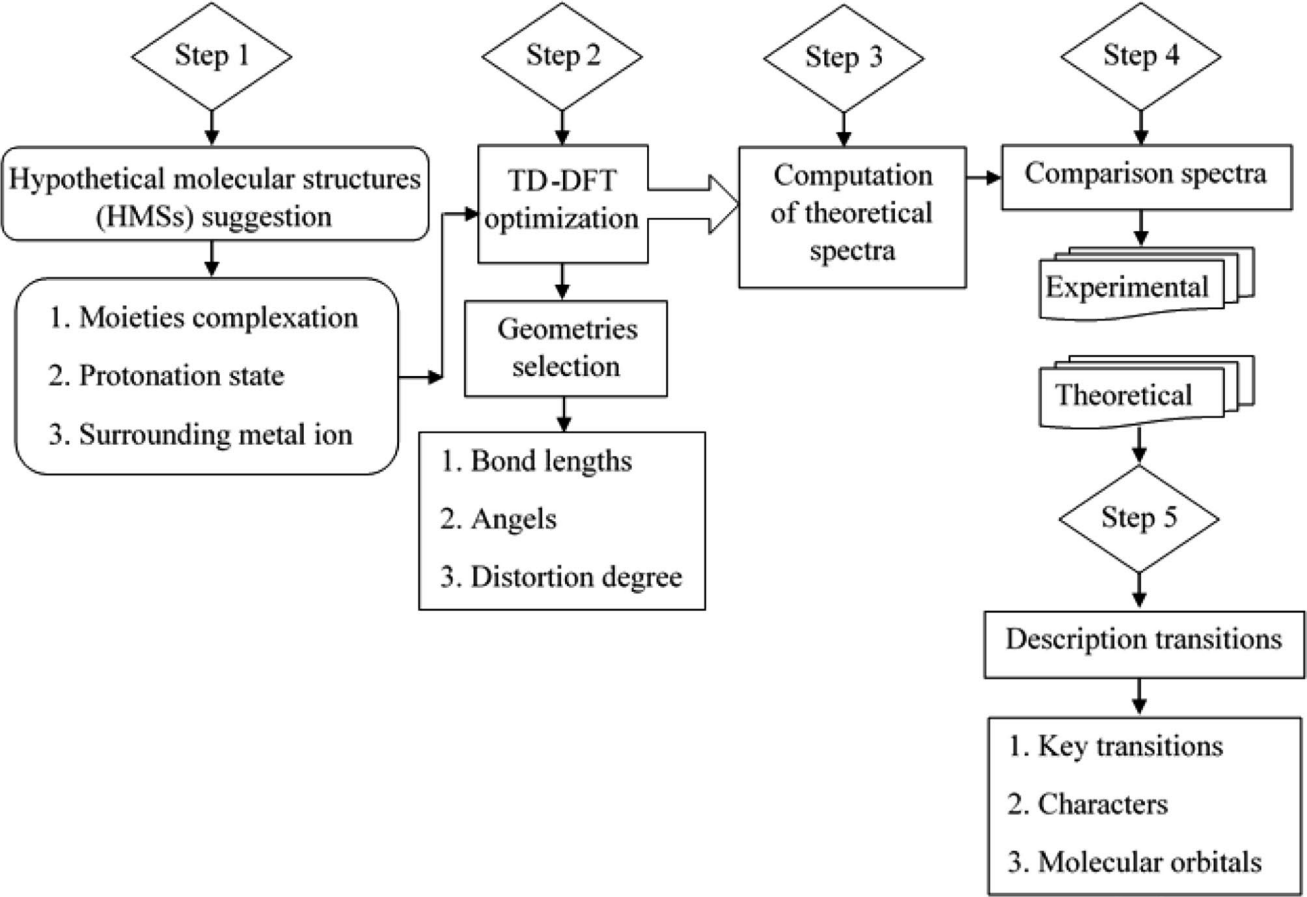
Steps of theoretical studies of the complexes.

The aim of the DFT study of the MI-MTB complexes is to obtain proper geometrical structures for the complexes that well reproduce experimental futures of the corresponding UV–Visible spectra. Then using the proper geometrical structures, we can compute the main contributor MOs and obtain the nature of the UV–Visible electronic transitions. As summarized in Fig. 5, the complete procedures of the DFT study of the complexes include five major steps: (1) suggesting HMSs different in protonation states and coordination sites of MTB and surroundings of the MI. For this purpose, we suggested 43 HMSs with octahedral (Oh), square planner (SP), and tetrahedral (Td) geometries for the MI(MTB) complexes, 22 HMSs with Oh geometry for MI_2_(MTB) complex, 74 HMSs with Oh, SP and Td geometries for MI(MTB)_2_ complexes (533 HMSs totally). (2) optimizing geometrical structures of the HMSs. The optimized HMSs were labeled to select a preferred geometry based on bond lengths, angles and distortion degree from a perfect geometry (241 HMSs were obtained). The observed geometries after optimization were Oh, SP, Td, square pyramidal (SPY) and trigonal bipyramidal (TBP). (3) Computing the theoretical wavelengths of the optimized HMSs (241 spectra were computed). (4) Comparing the computed wavelengths with experimental spectra to choose the most likely geometries of the complexes (primarily, 30 spectra were selected). (5) Describing the theoretical wavelengths of the proper HMSs. These include the electronic properties such as key transitions, characters, and contributor MOs (4 structures totally).

Generally, an MI may select definite binding geometries based on the number of its valance d electrons, surroundings, chelating agents and steric constraints. Zn^II^ has a d^10^ electron configuration and thus, has no geometric priority based on the ligand field stabilization energy^60^. Td and Oh geometries are the most common coordination spheres reported for Zn^II^ complexes and the Oh coordination is the most predominant one in solution phases^64–66^. Except for a few rare cases, complexes with the SP geometry for Zn^II^ has not been reported^67,68^. Furthermore, 5-coordinate square bipyramidal (SPY) and trigonal bipyramidal (TBP) geometries are also common in the coordination chemistry of Zn^II^^69,70^. On the other hand, Cu^II^ is usually found in a tetragonal coordination sphere. However, the Jahn–Teller distortions prevent the organization of a perfect hexavalent arrangement for Cu^II^^71^. Besides, 3- 5- 7-, and 8-coordinate complexes have also been reported for Cu^II^^72^. For Fe^II^, the most common arrangements are Oh and distorted Oh geometries. However, higher and lower coordination numbers are also reported for Fe^II^^73,74^. In the following sections, we will discuss the complexes of stoichiometry (MI:MTB) 1:1, 2:1, and 1:2.

#### DFT study of the 1:1 complexes

We have Suggested 43 HMSs for the MI(MTB) complexes according to the complexation moiety, protonation state of different functional groups of MTB and surroundings of the MIs. These 43 HMSs are shown in Fig. S5. The first set includes 25 HMSs without involving nitrogen moiety in the complexation process. Eight HMSs for the bidentate coordination on a carboxylate moiety and a hydroxyl moiety (HMSs 1–8), nine HMSs for the bidentate coordination on the two carboxylate moieties (HMSs 9–17), eight HMSs for the tridentate coordination on the two carboxylate moieties and a hydroxyl moiety (HMSs 18–25). The second set includes 18 HMSs involving nitrogen moiety in the complexation process. Four HMSs for the bidentate coordination on a hydroxyl moiety and a nitrogen moiety (HMSs 26–29), six HMSs for the tridentate coordination on two carboxylate moieties and a nitrogen moiety (HMSs 30 to 35), four HMSs for the tridentate coordination from a carboxylate moiety, a hydroxyl moiety and a nitrogen moiety (HMSs 36–39) and four HMSs four-dentate coordination on two carboxylate moieties, a hydroxyl moiety and a nitrogen moiety (HMSs 40–43). First, the optimizations were performed for Oh geometries assisted by water molecules to complete the coordination sphere. Then the SP and Td geometries were constructed from the optimized Oh geometries by adjusting or deleting the added water molecules. Finally, the constructed HMSs of SP and Td coordination spheres were also optimized (375 HMSs totally).

**Figure 6:**
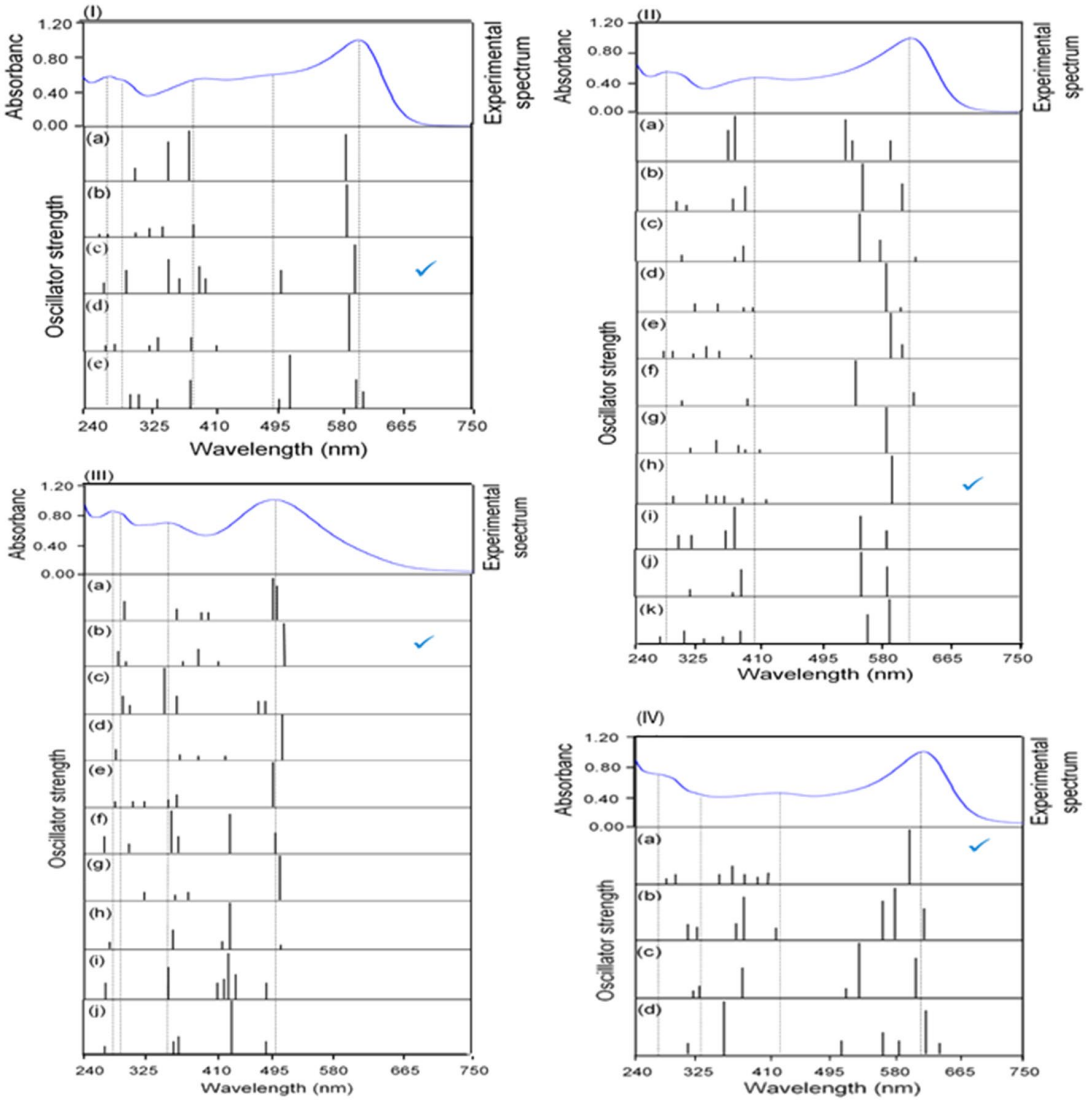
Computed wavelengths (black) and experimental spectrum (blue) for HMSs: (I) (**a**) 8-Oh(4), (**b**) 35-Oh(3), (**c**) **31-SP(1)**, (**d**) 23-SP(1) and (**e**) 20-Td(1) of Zn^II^ complexes, (II) computed wavelengths (black) and experimental spectrum (blue) for HMSs: (**a**) 8-Oh(4), (**b**) 21-Oh(1), (**c**) 27-Oh(4), (**d**) 37-Oh(3), (**e**) 38-Oh(3), (**f**) 40-Oh(2), (**g**) 16-SP(2), (**h) 28-SP(2)**, (**i**) 37-SP(1), (**j**) 38-SP(1) and (**k**) 39-SP(1) of Cu^II^ complexes, (III) computed wavelengths (black) and experimental spectrum (blue) for HMSs: (**a**) 14-Oh(4), (**b**) **15-Oh(4)**, (**c**) 24-Oh(4), (**d**) 34-Oh(3), (**e**) 9-SP(2), (**f**) 12-SP(2), (**g**) 14-SP(2), (**h**) 31-SP(1), (**i**) 32-SP(1) and (**j**) 12-Td(2) of Fe^II^ complexes and (IV) computed wavelengths (black) and experimental spectrum (blue) for HMSs: (**a**) **Oh-13(4)**, (**b**) Oh-16(3), (**c**) Oh-17(3) and (**d**) Oh-21(3) for the Fe^II^ complexes.

Some geometrical parameters for the preferred optimized geometries of the 1:1 complexes are listed in Tables S1 to S3 in the Supplementary Information (see Fig. S6 for naming of the atoms). Extracting a preferred geometry for a certain MI was performed based on bond lengths, angles and distortion degree from a perfect geometry. The optimized geometries include Oh, SP, Td, SPY and TBP coordination spheres around the MIs. To distinguish whether the geometry of the optimized coordination centers is SP or Td and TBP or SPY, the τ_4_′ and τ_5_ angular structural parameters were computed, respectively^75^. In addition, almost all of the optimized HMSs have a significant degree of distortion from the perfect coordination spheres. The optimized geometrical parameters of the selected HMSs are in a good agreement with the selected geometrical parameters in other works (for more details of geometrical parameters of preferred HMSs see page S25 of the Supplementary Information). After completing the optimization process of the HMSs for the 1:1 complexes, the theoretical spectra of them were computed. To check the validity of an optimized HMS, we considered the agreement between the experimental and computed LETs of that HMS. This method was previously used to assign valid structures for other molecules^76,77^. Tables S4 to S6 in the Supplementary Information provide the computed wavelengths, the difference between the computed and experimental wavelengths (Δλ) and oscillator strength values of LETs. Table 4 summarizes the results only for HMSs that have the lowest Δλ values for LETs. Computed wavelengths and experimental spectra for these HMSs are shown in Table S5 and Fig. 6I–III.

**Table 4:** Computed wavelengths (*λ*) and energy (*E*), difference between the theoretical and experimental values of wavelengths (Δ*λ*) and oscillator strengths (*f*) of LETs for the Zn(MTB), Cu(MTB), and Fe(MTB) complexes.

Complex	No. HMs	Transitions from 500 to 700 nm			Preferred geometry
		λ/nm (*E*/eV)	Δλ/nm (*E*, eV)	*f*	
Zn(MTB)	8-Oh(4)	584 (2.12)	16 (0.06)	0.26	Oh(3)
	35-Oh(3)	587 (2.11)	13 (0.05)	0.53	Oh(3)
	**31-SP(1)**	**595 (2.08)**	**5 (0.02)**	**0.22**	**SPY(1)**
	23-SP(1)	578 (2.14)	22 (0.08)	0.51	SPY(1)
	20-Td(1)	606 (2.05)	6 (0.02)	0.09	Td(1)
	Experiment	600 (2.07)			
Cu(MTB)	8-Oh(4)	588 (2.11)	12 (0.04)	0.08	SPY(2)
	21-Oh(1)	593 (2.09)	7 (0.02)	0.17	SPY(1)
	27-Oh(4)	614 (2.02)	14 (0.05)	0.05	SPY(3)
	37-Oh(3)	592 (2.09)	8 (0.03)	0.06	SPY(2)
	38-Oh(3)	593 (2.09)	7 (0.02)	0.14	SPY(2)
	40-Oh(2)	608 (2.04)	8 (0.03)	0.14	SPY(1)
	16-SP(2)	578 (2.15)	22 (0.08)	0.57	SPY(2)
	**28-SP(2)**	**586 (2.12)**	**14 (0.05)**	**0.56**	**SPY(2)**
	37-SP(1)	585 (2.12)	15 (0.05)	0.23	SPY(1)
	38-SP(1)	588 (2.11)	12 (0.04)	0.31	SPY(1)
	39-SP(1)	586 (2.12)	14 (0.05)	0.12	SPY(1)
	Experiment	600 (2.07)			
Fe(MTB)	14-Oh(4)	501 (2.47)	3 (0.02)	0.27	Oh(4)
	**15-Oh(4)**	**517 (2.40)**	**19 (0.09)**	**0.51**	**Oh(4)**
	24-Oh(3)	486 (2.55)	12 (0.06)	0.11	Oh(3)
	34-Oh(3)	518 (2.40)	19 (0.09)	0.61	Oh(3)
	9-SP(2)	497 (2.49)	1 (0.00)	0.53	SP(2)
	12-SP(2)	484 (2.56)	14 (0.07)	0.13	SPY(2)
	14-SP(2)	508 (2.44)	10 (0.05)	0.61	SPY(2)
	31-SP(1)	507 (2.45)	9 (0.04)	0.50	SPY(1)
	32-SP(1)	491 (2.52)	7 (0.04)	0.07	SPY(1)
	12-Td(2)	493 (2.52)	5 (0.03)	0.11	Oh(2)
	Experiment	498 (2.49)			

The significances of bold show the final optimized HMSs for the Zn(MTB), Cu(MTB), and Fe(MTB) complexes.

The number in parentheses indicates the number of water molecules involved in the coordination sphere.

First, to check the validity of a computed spectrum, the LET of the computed wavelengths is considered and is compared with the experimental value. Then, all the electronic transitions of the computed spectrum are considered to suggest a dominant structure. The experimental LETs for both the Zn(MTB) and Cu(MTB) complexes appear at 600 nm. The other spectral features for Zn(MTB) complex reveal a shoulder at 490 nm, that is well reproduced with a value of 500 nm (*f* = 0.12) only by the HMS-31-SP(1) (the first number indicates the HMS number shown in Fig. S5, SP indicated that the metal center has an SP coordination, and the second number in parenthesis indicates that there is a water molecule in the coordination center, cf. Fig. 6I(c). For HMS-31-SP(1), the computed wavelength at 595 nm (*f* = 0.22) has the closest value to the experimental one at 600 nm. The computed wavelengths for HMS-31-SP(1) reproduce all the features of the experimental spectrum for the Zn(MTB) complex. For Cu^II^, the computed wavelength at 586 nm (*f* = 0.56) for the HMS-28-SP(2) has the closest value to 600 nm. Also, the experimental wavelength at 285 nm is well reproduced with a value of 295 nm (*f* = 0.08) by HMS-28-SP(2) [cf. Fig. 6II(h)]. For Fe(MTB), the HMS-15-Oh(4) reproduces all the properties of the experimental spectrum quite well [cf. Fig. 6III(b)].

**Figure 7:**
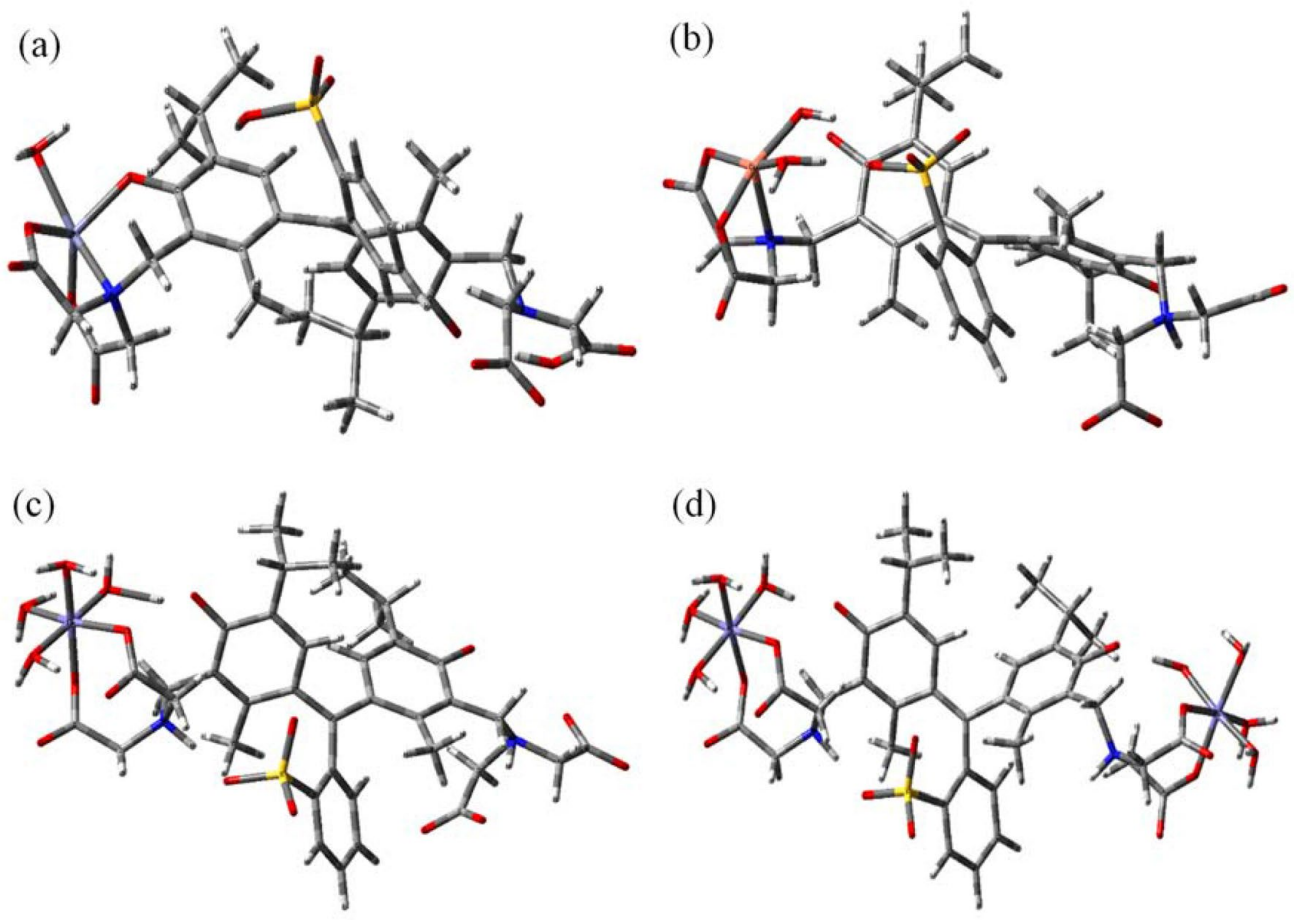
Optimized structures of the (**a**) Zn(MTB), (**b**) Cu(MTB), (**c**) Fe(MTB) and (**d**) Fe_2_(MTB) complexes.

The computed and experimental wavelengths for the proper 1:1 HMSs are collected in Table 5 and their optimized structures are depicted in Fig. 7. The τ_5_ parameter for the Zn(MTB) and Cu(MTB) structures are 0.44 and 0.12, respectively. These represent a highly distorted SPY geometry for Zn^II^ and a slightly distorted SPY geometry for Cu^II^ (for perfect SPY and TBP geometries τ_5_ = 0 and 1, respectively).

**Table 5:** Experimental and computed absorption wavelengths of Zn(MTB), Cu(MTB), Fe(MTB), and Fe_2_(MTB) complexes (the optimized structures of the complexes are shown in Fig. 6), and MO contributions (≥ %10) involved in each transition.

Complex	Exp. λ/nm (ev)	Com. λ/nm (ev)	Key transitions	*f*	Character
Zn(MTB)	600 (2.07)	595 (2.08)	Ho − 1 → Lu (93%)	0.22	LLCT
	490 (2.53,sh)	500 (2.48)	Ho − 1 → Lu + 1 (94%)	0.12	LLCT
	400 (3.10)	406 (3.05)	Ho − 5 → Lu (10%), Ho − 6 → Lu (23%), Ho − 7 → Lu (45%), Ho − 8 → Lu (13%)	0.08	LLCT
		399 (3.11)	Ho − 6 → Lu (48%), Ho − 7 → Lu (29%)	0.13	LLCT
		370 (3.35)	Ho − 1 → Lu + 1 (75%), Ho − 6 → Lu + 1 (14%)	0.07	LLCT
		353 (3.51)	Ho − 6 → Lu + 1 (56%)	0.18	LLCT
	300 (4.14,sh)	306 (4.05)	Ho − 1 → Lu + 3 (47%), Ho − 15 → Lu (11%), Ho − 15 → Lu (23%)	0.11	LLCT
	276 (4.49)	272 (4.56)	Ho − 1 → Lu + 4 (53%), Ho − 1 → Lu + 4 (23%)	0.06	LLCT
Cu(MTB) Fe(MTB)	600 (2.07)	586 (2.11)	αHo → *α*Lu (49%), *β*Ho → *β*Lu + 1 (49%)	0.56	LLCT
	400 (3.10)	411 (3.02)	*β*Ho − 8 → *β*Lu (90)	0.05	LMCT/LLCT
		385 (3.22)	αHo − 9 → *α*Lu (21%), *β*Ho − 9 → *β*Lu + 1 (28%)	0.07	LLCT
		360 (3.44)	αHo → *α*Lu + 2 (40%), *β*Ho → *β*Lu + 1 (40%)	0.10	LLCT
		345 (3.60)	αHo − 13 → *α*Lu (42%), *β*Ho − 13 > *β*Lu + 1 (42%)	0.09	LLCT
		335 (3.70)	αHo → *α*Lu + 3 (47%), *β*Ho → *β*Lu + 4 (48%)	0.15	LLCT
	285 (4.35)	295 (4.20)	αHo → *α*Lu + 4 (36%), *β*Ho → *β*Lu + 5 (37%)	0.08	LLCT
	498 (2.49)	517 (2.40)	Ho − 1 → Lu (14%), Ho − 2 → Lu (79%)	0.51	LLCT
	354 (3.50)	411 (3.02)	Ho − 7 → Lu (10%), Ho − 8 → Lu (25%), Ho − 11 → Lu (35%), Ho − 12 → Lu (20%)	0.05	MLCT/LLCT,LLCT
		392 (3.16)	Ho − 7 → Lu (26%), Ho − 8 → Lu (27%), Ho − 11 → Lu (24%), Ho − 12 → Lu (12%)	0.22	MLCT/LLCT,LLCT
		374 (3.31)	Ho − 13 → Lu (90%)	0.06	LLCT
	292 (4.25,sh)	302 (4.11)	Ho − 1 → Lu + 2 (34%), Ho − 2 → Lu + 2 (42%)	0.06	LLCT/LMCT
	282 (4.40)	286 (4.34)	Ho − 21 → Lu (13%), Ho − 23 → Lu (10%), Ho − 24 → Lu (53%)	0.19	LLCT
Fe_2_(MTB)	622 (1.99)	607 (2.04)	Ho → Lu (99%)	0.42	LLCT
	420 (2.95)	411 (3.01)	Ho − 7 → Lu (41%), Ho − 8 > Lu (38%), Ho − 9 > Lu (13%)	0.08	MLCT/LLCT,LLCT
		383 (3.24)	Ho − 7 → Lu (23%), Ho − 9 > Lu (57%), Ho − 10 > Lu (15%)	0.10	MLCT/LLCT,LLCT
		363 (3.42)	Ho − 7 → Lu (12%), Ho − 10 → Lu (14%), Ho − 11 → Lu (64%)	0.16	MLCT/LLCT,LLCT
		332 (3.73)	Ho → Lu + 2 (83%), Ho → Lu + 1 (12%)	0.11	LLCT/LMCT
	295 (4.20)	304 (4.08)	Ho − 2 → Lu + 4 (14%), Ho − 17 → Lu (36%), Ho − 18 → Lu (22%)	0.06	MMCT, LLCT
	280 (4.43)	296 (4.19)	Ho − 17 → Lu (19%), Ho − 18 → Lu (15%), Ho − 19 → Lu (14%), Ho − 22 → Lu (20%)	0.06	LLCT

Phrase LMCT stands for ligand-to-metal charge transfer.

**Figure 8:**
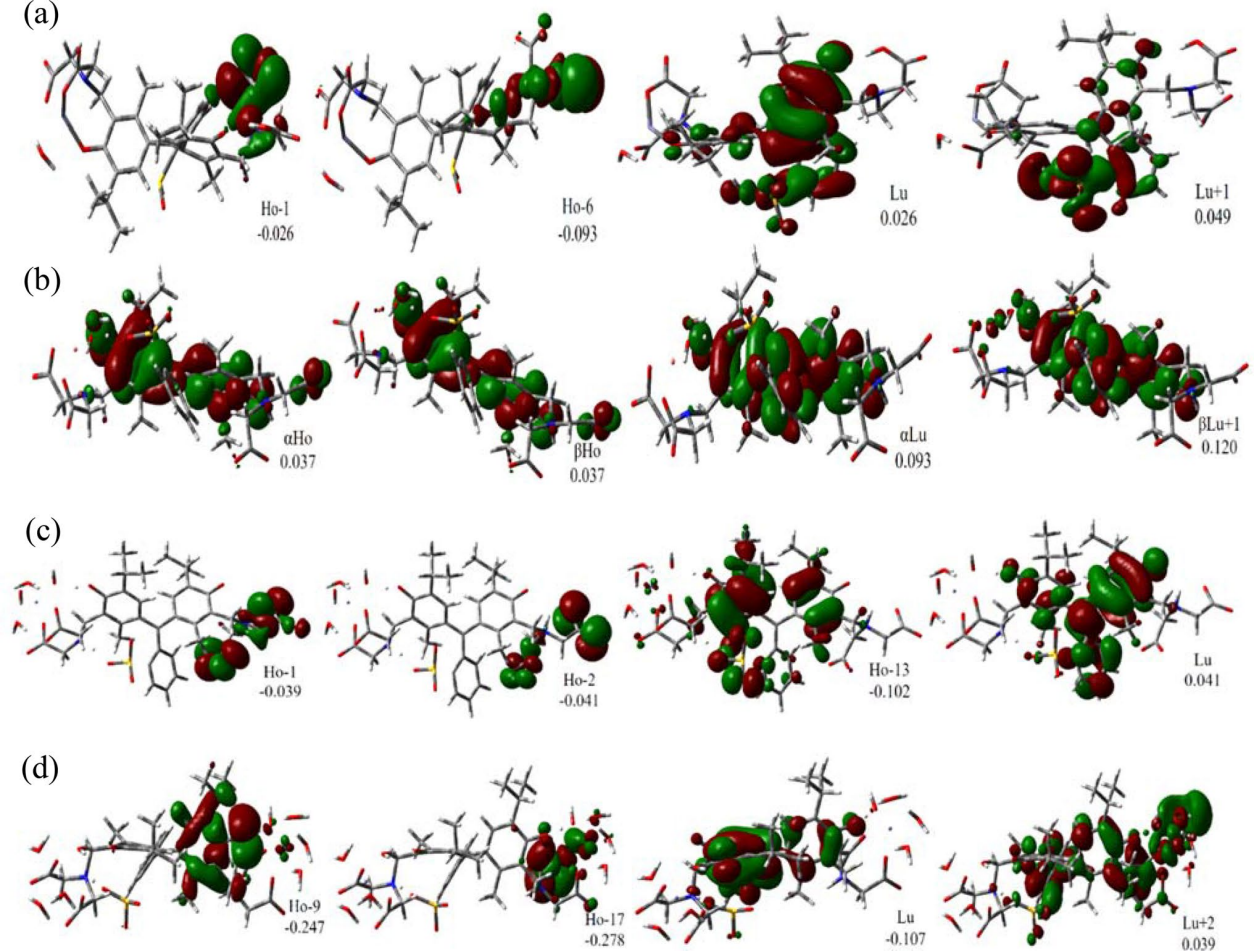
Representation some of MOs involved in the major electronic excitations for (**a**) Zn(MTB), (**b**) Cu(MTB), (**c**) Fe(MTB) and (**d**) Fe_2_(MTB) complexes, respectively.

The computed frontier MOs of the 1:1 complexes are shown in Fig. 8 and their distributions on the complexes are listed in Table 6. The character assignment for MOs was based on their composition and a visual searching in their 3D images.

**Table 6:** Compositions of some MOs for the Zn(MTB), Cu(MTB), Fe(MTB) and Fe_2_(MTB) complexes, respectively.

Complex	MO	%Comp		MO	%Comp			
		Ligand	Zn^II^		Ligand		Zn^II^	
Zn(MTB)	Ho-1	100	0	L	100		0	
	Ho-5	99	1	L + 1	100		0	
	Ho-6	100	0	L + 3	100		0	
	Ho-7	99	1	L + 4	99		1	
	Ho-8	100	0					
	Ho-15	97	3					

Complex	MO	%Comp		MO	%Comp			
		Ligand	Cu^II^		Ligand		Cu^II^	
Cu(MTB)	αHo	100	0	αL	100		0	
	*β*Ho	100	0	*β*L + 1	99		1	
	*β*Ho-8	100	0	*β*L	36		64	
	αHo-9	100	0	αL + 2	100		0	
	*β*Ho-9	100	0	αL + 3	94		6	
	αHo-13	97	3	*β*L + 4	95		5	
	*β*Ho-13	95	5	αL + 4	100		0	
				*β*L + 5	100		0	

Complex	MO	%Comp		MO	%Comp			
		Ligand	Fe^II^		Ligand		Fe^II^	
Fe(MTB)	Ho-1	100	0	L	100		0	
	Ho-2	100	0	L + 2	59		41	
	Ho-7	15	85					
	Ho-8	18	82					
	Ho-11	100	0					
	Ho-12	100	0					
	Ho-13	100	0					
	Ho-21	99	1					
	Ho-23	94	6					
	Ho-24	97	3					

Complex	MO	%Comp			MO	%Comp		
		Ligand	Fe^II^(1)	Fe^II^(2)		Ligand	Fe^II^(1)	Fe^II^(2)
Fe_2_(MTB)	Ho	99	1	0	L	100	0	0
	Ho-2	15	0	85	L + 1	71	29	0
	Ho-7	18	82	0	L + 2	79	21	0
	Ho-8	99	0	1	L + 4	15	0	85
	Ho-9	99	1	0				
	Ho-10	100	0	0				
	Ho-11	99	0	1				
	Ho-17	97	0	3				
	Ho-18	92	0	8				
	Ho-19	100	0	0				
	Ho-22	96	0	4				

For the Zn(MTB) complex, the Ho − 1, Ho − 6, and Ho − 7 orbitals have mainly n and π characters and are located on the carboxylate and nitrogen moieties (cf. Figure 8a, also Figure S7). On the other hand, the LUMOs (Lu, Lu + 1, Lu + 2, Lu + 3, and Lu + 4) are localized on the sulfonic moiety and benzene rings with a π* character. The results show that the contribution of the Zn^II^ ion in the HOMOs and LUMOs is very small (1–3%). On the other hand, the ligand contribution changes from 97 to 100% and 99 to 100% in HOMOs and LUMOs, respectively (cf. Table 6). So that, all transitions observed in the UV–Visible spectrum of the Zn(MTB) complex can be attributed to a LLCT character (cf. Table 5). For the Cu(MTB) complex, the HOMOs are mainly distributed on the benzene rings with a π character (cf. Figure 8b, also Figure S7). The only exceptions are αHo − 13 and βHo − 13 which are mainly localized on one of the carboxylate moieties with a π character. For the Cu containing systems the multiplicity is doublet and α and β represents orbitals with spin up and down electrons, respectively. The LUMOs essentially have a π* character and are localized on the benzene rings. The only exception is *β* − Lu which its main contributor is from the Cu^II^ ion (64%). According to Table 6, most transitions observed for the Cu(MTB) complex can be attributed to the LLCT character. For the Fe(MTB) complex, the Ho − 1, Ho − 2, Ho − 11, Ho − 13, and Ho − 24 orbitals are mostly distributed on the carboxylate moieties with a mixed n and π character (cf. Fig. 8c, also Fig. S7). The Ho − 7 and Ho − 8 orbitals are very similar to each other and are mainly distributed on Fe^II^. The Lu and Lu + 2 orbitals have a π* character, are localized on the benzene rings and the carboxylate moieties, respectively. Unlike the Zn(MTB) and Cu(MTB) complexes, the contribution of the Fe^II^ ion at the computed wavelengths is significant (82–85% in HOMOs and 41% in LUMOs, cf. Table 6). This leads to various characters of observed transitions for the Fe(MTB) complex (cf. Table 5).

#### DFT study of the 2:1 and 1:2 complexes

In this work, the only complex of the 2:1 stoichiometry is Fe_2_(MTB). Since the results of the previous section revealed that Fe^II^ prefers Oh geometry for its 1:1 complex, we suggested 22 HMSs with Oh geometry for the Fe_2_(MTB) complex (cf. Fig. S8). Some geometrical parameters for the preferred geometries of the MI(MTB) complexes are listed in Table S8 in the Supplementary Information. Table S9 in the Supplementary Information provides the Δ*λ*, λ and *f* values of LETs for all the optimized HMSs of Fe_2_(MTB). Among the 22 HMSs, HMS-13(4) well reproduces the experimental spectrum of Fe_2_(MTB) complex (cf. Fig. 6IV, also Table S7**).** Then we propose this HMS as the Fe_2_(MTB) complex structure (cf. Fig. 7d for the structure). The computed wavelengths for this HMS are collected in Table 5. The computed wavelength at 607 nm (*f* = 0.42) has the closest value to the experimental one at 622 nm. The Ho → Lu transition with 99% contribution is the major source for this transition. The computed wavelengths at 411, 383, 363, and 332 nm can be attributed to the experimental wavelength at 420 nm. However, the wavelengths at 280 and 290 nm were predicted with relatively low *f* values*.* In the optimized geometry of Fe_2_(MTB) complex, both hydroxyl moieties and nitrogen atoms are deprotonated and protonated, respectively. In this HMS, both Fe^II^ ions are coordinated by two carboxylic oxygen and four water molecules giving an Oh geometry to it (cf. Fig. 7d).

Some of the selected frontier MOs for the Fe_2_(MTB) complex are shown in Fig. 8d (also cf. Fig. S7) and the atomic contributions in MOs are provided in Table 6. The main occupied orbitals (Ho − 7, Ho − 8, Ho − 9, Ho − 13, Ho − 17, Ho − 18, and Ho − 19) are mainly localized on the carboxylate moieties with different contributions from the sulfonic moiety and the Fe^II^ ions. The only exception is the Ho orbital which is distributed on the benzene rings. All of these MOs have a mixed n and π character. The unoccupied orbitals are distributed over the three aromatic rings. However, Lu + 2 is also distributed on one of the carboxylate moieties and one of the Fe^II^ ions. According to Table 5, the LET is a Ho → Lu transition. The results show that 99% of the Ho and 100% of Lu orbitals are distributed on MTB (cf. Table 6). This implies that the LET has a LLCT character. However, the higher energy transitions are a mixture of LLCT, MLCT, and MMCT.

To search for the preferred HMSs of the 1:2 complexes, we optimized 74 HMSs with Oh, Td, and SP geometries (cf. Fig. S9 in the Supplementary Information). Among these HMSs, 19 geometries were successfully optimized (cf. Table S10). The others were encountered convergence errors. None of the computed UV–Visible transitions of the 19 optimized HMSs was in good agreement with the experimental spectrum. MI(MTB)_2_ HMSs have ~ 200 atoms. This number of atoms in quantum chemical and DFT systems makes the calculation very robust and time-consuming. In summary, for the very big system of MI(MTB)_2_ complexes we cannot reach a realistic geometry by DFT calculations.

## Conclusions

In summary, we investigated the acid–base properties of free MTB and its complexation systems with the Zn^II^, Cu^II^, and Fe^II^ through UV–Visible spectroscopy and theoretical means. The acidity constants of MTB were determined using MLA with residual and standard errors less than 0.0086 and 0.12, respectively. DFT calculations well reproduced spectral futures of MTB_H4_, MTB_H6_, and MTB_H0_. The electronic transitions in the UV–Visible spectra of these species have mixed n → π* and π → π* characters.

The plots extracted from the molar ratio method and MCR-ALS model were used to determine the stoichiometry of complexes. Both propose the formation of successive 1:2 and 1:1 complexes for the Zn^II^ and Cu^II^. However, the results show successive 1:1 and 2:1 complexes for Fe^II^. The association constants of the complexes are well defined using RAFA with standard deviations values < 0.06. The correlation between the experimental and computed wavelengths revealed that the Zn(MTB) and Cu(MTB) complexes prefer SPY geometries. In both, the MI binds to fully deprotonated carboxylic moieties and nitrogen atom. However, the coordination sphere of Zn^II^ also involved a hydroxyl moiety. In the case of Fe^II^, the Fe(MTB) and Fe_2_(MTB) complexes always show Oh geometry. These complexes involved a fully deprotonated carboxylic moiety and four water molecules. Our results show that the majority of transitions observed in the UV–Visible spectra of the Zn(MTB) and Cu(MTB) complexes have LLCT character. However, the transitions in the UV–Visible spectrum of the Fe(MTB) complex have LLCT and MLCT characters. The LET of Fe_2_(MTB) has an LLCT character. However, its higher energy transitions are a mixture of LLCT, MLCT and MMCT characters. In the case of Zn^II^ and Cu^II^, the natures of the 1:2 complexes were not characterized. Actually, including two MTB increases the number of atoms and electrons that cause the quantum calculations very robust and time-consuming.

## Supplementary information

Supplementary information.
