# Supplementary material for: Study of interaction of metal ions with methylthymol blue by chemometrics and quantum chemical calculations
Source: Sci Rep. 2021 Mar 19;11:6465. doi: 10.1038/s41598-021-85940-w (PMC7979746; doi:10.1038/s41598-021-85940-w)
Supplement: Supplementary file 1 — Supplementary information. [file 41598_2021_85940_MOESM1_ESM.docx]

**Supplementary Information**

**Study of Interaction of Metal Ions with Methylthymol Blue by Chemometrics and Quantum Chemical Calculations**

Zolaikha Rasouli,^1^ Mehdi Irani,^2^ Sonia Jafari,^2^ Raouf Ghavami^1,^*

^1^Chemometrics Laboratory, Chemistry Department, Faculty of Science, University of Kurdistan, P.O. Box 416, Sanandaj 66177-15175, Iran

^2^Theoretical Chemistry Laboratory, Chemistry Department, Faculty of Science, University of Kurdistan, P.O. Box 416, Sanandaj 66177-15175, Iran

Corresponding Author: r.ghavami@uok.ac.ir; rghavami2000@yahoo.com

**Figure S1.** Absorption spectra and digital photographs of the MTB solution at pH 5 over 1 hour.


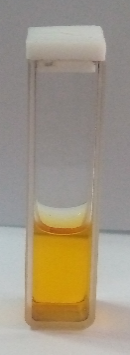

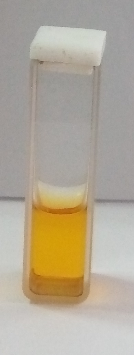


t= 0 min

t= 60 min


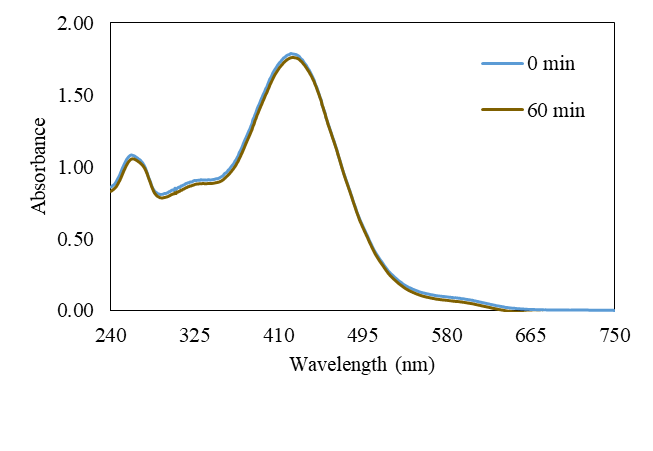


**Marquardt-Levenberg Algorithm (MLA)**. The Marquardt-Levenberg algorithm (MLA) is a standard procedure employed to solve non-linear least squares (LS) problems. Non-linear LS problems occur when the objective function is not linear in parameters. This algorithm was introduced in 1944 by Levenberg and was developed by Marquardt in 1963.^1^ Conventional and modified MLA in a wide variety of problems that are employed to determine unknown parameters and functions in different equilibrium and kinetic systems.^2-8^ The fitting of objective functions in MLA is an arrangement of two minimization algorithms: the gradient descent algorithm and the Gauss-Newton algorithm (GNA). In the gradient descent, the sum of squared (**ssq**) of residuals is decreased by updating the parameters in the steepest-descent direction. In the Gauss-Newton algorithm, the ssq of residuals is decreased assuming that the LS function is locally quadratic, and finding the minimum of the quadratic function. The performance of MLA is similar to the gradient descent algorithm when the parameters are far from their optimal values, and its performance is similar to the GNA algorithm when the parameters are close to their optimal values.^9,10^

In the MLA, like other numerical minimization algorithms, the fitting of vector **p** of parameters is an iterative procedure. The most common method used for this task is NGA. The equations governing the NGA are based on a truncated Taylor series expansion. To start minimizing, there must be a starting guess for the vector **p** of parameters. The values of the vector are iteratively fitted by a Jacobian-based relationship that results in the minimal **ssq** of the residuals. Then, the derivatives of the vector **r** of residuals concerning the vector **p** of parameters are calculated. The derivative ∂**r**(**p**)/∂**p** is known as the Jacobian **J**:

$$\boldsymbol{J=}\frac{\boldsymbol{\delta r(p)}}{\boldsymbol{\delta p}}\boldsymbol{(1)}$$

Subsequently, the vector **p** of parameters is determined by adding an appropriate parameter shift-vector **δp_i_**:

$$\boldsymbol{J\cong}\frac{\boldsymbol{r}\left( \boldsymbol{p+\delta}\boldsymbol{p}_{\boldsymbol{i}} \right)\boldsymbol{-r}\left( \boldsymbol{p} \right)}{\boldsymbol{\delta}\boldsymbol{p}_{\boldsymbol{i}}}\boldsymbol{(2)}$$

Here, ($\boldsymbol{p+\delta}\mathbf{p}_{\mathbf{i}}$) is a new vector **p** of parameters that shifted by the small value $\boldsymbol{\delta}\mathbf{p}_{\mathbf{i}}$. Typically, $\boldsymbol{\delta}\mathbf{p}_{\mathbf{i}}$ was computed as 1 × 10^-4^ $\mathbf{p}_{\mathbf{i}}$. The iterative refinement of the vector **p** of parameters is given by the below equation:

$$\boldsymbol{\delta p=-}\boldsymbol{J}^{\boldsymbol{+}}\boldsymbol{r (3)}$$

The shift vector $\boldsymbol{\delta p}$ is calculated and added to the vector **p** of parameters. If decreasing of the **ssq** of the residuals for the last set of the vector **p** of parameters is less than the predetermined values, then, the iteration ends and the last the vector **p** of parameters is considered as the answer. Commonly, this results in convergence (i.e., decrease in **ssq**). About the divergence (i.e., increase in **ssq**), the Marquardt parameter **mp** is added to the diagonal elements of **J**. Increasing the **mp** shortens $\boldsymbol{\delta p}$ and conducts it to the path of steepest descent. The standard error $\boldsymbol{\sigma}_{\mathbf{p}_{\mathbf{i}}}$in the fitted vector **p** of parameters can be assessed from the following equation:

$$\boldsymbol{\sigma}_{\boldsymbol{P}_{\boldsymbol{i}}}\boldsymbol{=}\boldsymbol{\sigma}_{\boldsymbol{r}}\sqrt{\boldsymbol{d}_{\boldsymbol{i,j}}}\boldsymbol{(4)}$$

In which, $\mathbf{d}_{\mathbf{i,j}}$ is the *i*^th^ diagonal element of the matrix ${\mathbf{(}\mathbf{J}^{\mathbf{+}}\mathbf{J)}}^{\mathbf{-1}}$, and $\boldsymbol{\sigma}_{\mathbf{r}}$ indicates standard deviation of the residuals **r** that expresses as:

$$\boldsymbol{\sigma}_{\boldsymbol{r}}\boldsymbol{=}\sqrt{\frac{\boldsymbol{ssq}}{\boldsymbol{df}}}\boldsymbol{(5)}$$

In which, $\mathbf{df}$ is the degree of freedom that defined as the number of experimental values **m** subtracted by the number of optimized parameters **np**:

$$\boldsymbol{df=m-np (6)}$$

Flowchart S1 displays a diagram of the Newton-Gauss-MLA algorithm.

Guess parameters, **p**=**p**_start;_ initial value for **mp**

Calculate residuals, **r** and the sum of squares, **ssq**

I

$$\mathbf{ssq}_{\mathbf{old}}\mathbf{<}\approx>ssq$$

$$\mathbf{mp}\boldsymbol{=0}$$

$$\mathbf{mp}\boldsymbol{=0}$$

$$\mathbf{mp}\boldsymbol{/3}$$

$$\mathbf{mp}\boldsymbol{\times5}$$

Calculate Jacobian **J**

End;

Display results

Calculate of shift vector $\boldsymbol{\delta p}$;

$$\boldsymbol{p=p+\delta p}$$

**Flowchart S1.** A diagram showing the Newton-Gauss-MLA algorithm.

As can be seen from Flowchart S1, the central core of NGA is the calculation of the residuals that can be collected in the matrix **R**. **R** which is a function of the vector **p** of parameters and model. Thus, for a given model we can write:

$$\boldsymbol{R=f}\left( \boldsymbol{p,model} \right)\boldsymbol{(7)}$$

It is obvious that there are both linear and non-linear parameters and hence, they can be resolved from each other. The vector **p** parameters define the matrix **C** of concentrations, according to the chemical model. The matrix **C**, in turn, allows the computation of the matrix **A** as:

$$\boldsymbol{R=Y-CA=Y-C}\left( \boldsymbol{C}^{\boldsymbol{+}}\boldsymbol{A} \right)\boldsymbol{=f}\left( \boldsymbol{Y,p} \right)\boldsymbol{(8)}$$

In which, **Y** is measurements. The main feature of equation (8) is that **R** is defined as a function of the vector **p** only. Nevertheless, the matrix **A** is always based on the matrix **C**. Consequently, during the iterative refinement, when the vector **p** parameters are still incorrect, **C** and **A** also are incorrect. Only in the end they will be correct.^11,12^

The principals of the application of modified Marquardt-Levenberg algorithms in the estimation of different non-linear parameters for chemical equilibria and kinetics systems were completely described in the book of practical data analysis in chemistry.^1^ In the current study, the MLA was modified and used for extraction of the acidity constants of MTB. Here, the non-linear parameters to be fitted are the acidity constants of MTB (i.e.,$K_{1},K_{2}{,K}_{3}{,K}_{4}, K_{5} and K_{6}$). Assuming that MTB is a six-protic acid $H_{6}A$, according to classical analytical chemistry, the variation of the concentrations of active chemical species of H_6_A can be estimated according to the following equations:

$$\left[ H_{6}A \right]=\frac{(C_{0}\left[ H^{+} \right]^{6})}{{(K}_{1}K_{2}K_{3}K_{4}K_{5}K_{6}+K_{1}K_{2}K_{3}K_{4}K_{5}[H^{+}]+K_{1}K_{2}K_{3}K_{4}\left[ H^{+} \right]^{2}+K_{1}K_{2}K_{3}\left[ H^{+} \right]^{3}+K_{1}K_{2}\left[ H^{+} \right]^{4}+K_{1}\left[ H^{+} \right]^{5}+\left[ H^{+} \right]^{6})} (9)$$

$$\left[ H_{5}A \right]=\frac{(K_{1}C_{0}\left[ H^{+} \right]^{5})}{{(K}_{1}K_{2}K_{3}K_{4}K_{5}K_{6}+K_{1}K_{2}K_{3}K_{4}K_{5}[H^{+}]+K_{1}K_{2}K_{3}K_{4}\left[ H^{+} \right]^{2}+K_{1}K_{2}K_{3}\left[ H^{+} \right]^{3}+K_{1}K_{2}{[H^{+}]}^{4}+K_{1}{[H^{+}]}^{5}+{[H^{+}]}^{6}} (10)$$

$$\left[ H_{4}A \right]=\frac{(K_{1}K_{2}C_{0}\left[ H^{+} \right]^{4})}{{(K}_{1}K_{2}K_{3}K_{4}K_{5}K_{6}+K_{1}K_{2}K_{3}K_{4}K_{5}[H^{+}]+K_{1}K_{2}K_{3}K_{4}\left[ H^{+} \right]^{2}+K_{1}K_{2}K_{3}\left[ H^{+} \right]^{3}+K_{1}K_{2}{[H^{+}]}^{4}+K_{1}{[H^{+}]}^{5}+{[H^{+}]}^{6}} (11)$$

$$\left[ H_{3}A \right]=\frac{(K_{1}K_{2}K_{3}C_{0}\left[ H^{+} \right]^{3})}{{(K}_{1}K_{2}K_{3}K_{4}K_{5}K_{6}+K_{1}K_{2}K_{3}K_{4}K_{5}[H^{+}]+K_{1}K_{2}K_{3}K_{4}\left[ H^{+} \right]^{2}+K_{1}K_{2}K_{3}\left[ H^{+} \right]^{3}+K_{1}K_{2}{[H^{+}]}^{4}+K_{1}{[H^{+}]}^{5}+{[H^{+}]}^{6}} (12)$$

$$\left[ H_{2}A \right]=\frac{(K_{1}K_{2}K_{3}K_{4}C_{0}\left[ H^{+} \right]^{2})}{{(K}_{1}K_{2}K_{3}K_{4}K_{5}K_{6}+K_{1}K_{2}K_{3}K_{4}K_{5}[H^{+}]+K_{1}K_{2}K_{3}K_{4}\left[ H^{+} \right]^{2}+K_{1}K_{2}K_{3}\left[ H^{+} \right]^{3}+K_{1}K_{2}{[H^{+}]}^{4}+K_{1}{[H^{+}]}^{5}+{[H^{+}]}^{6}} (13)$$

$$\left[ HA \right]=\frac{(K_{1}K_{2}K_{3}K_{4}K_{5}C_{0}{[H}^{+}])}{{(K}_{1}K_{2}K_{3}K_{4}K_{5}K_{6}+K_{1}K_{2}K_{3}K_{4}K_{5}[H^{+}]+K_{1}K_{2}K_{3}K_{4}\left[ H^{+} \right]^{2}+K_{1}K_{2}K_{3}\left[ H^{+} \right]^{3}+K_{1}K_{2}{[H^{+}]}^{4}+K_{1}{[H^{+}]}^{5}+{[H^{+}]}^{6}} (14)$$

$$\left[ A \right]=\frac{(K_{1}K_{2}K_{3}K_{4}K_{5}K_{6}C_{0})}{{(K}_{1}K_{2}K_{3}K_{4}K_{5}K_{6}+K_{1}K_{2}K_{3}K_{4}K_{5}[H^{+}]+K_{1}K_{2}K_{3}K_{4}\left[ H^{+} \right]^{2}+K_{1}K_{2}K_{3}\left[ H^{+} \right]^{3}+K_{1}K_{2}{[H^{+}]}^{4}+K_{1}{[H^{+}]}^{5}+{[H^{+}]}^{6}} (15)$$

In which, $\mathbf{C}_{\mathbf{0}}$ is the initial concentration of the $H_{6}A$ solution. The $\mathbf{d}_{\mathbf{i,j}}$ values are elements of the data set **D** (absorbance values at *i*^th^ pH and *j*^th^ wavelength). They are produced by spectrophotometric-pH-metric titration of MTB and measuring UV-Vis spectra at each pH. In this method, we start from the initial guesses for the acidity constants. Then they iteratively improved to result in the minimal **ssq** of the **R**. In iterations, the concentration of each chemical species is computed from the acidity constants following equations (9-15). These concentrations form the matrix of concentration profiles **C**. Subsequently, the matrix **A** is computed based on the matrix **C** (see Eq. (8)). The cycle estimation of acidity constants continues until the value of **r** is not changed.

**Soft Modeling: Multivariate Curve Resolution-Alternating Least Squares.** Multivariate curve resolution-alternating least square (MCR-ALS) analysis is a chemometrics soft model that decomposes a matrix **D** as a product of two matrices **C** containing the concentration profiles and **S** containing the spectral profiles for each the **r** chemical species of a multi-component system. Mathematically, it can be expressed by Eq. (1):

$$\boldsymbol{D=C}\boldsymbol{S}^{\boldsymbol{T}}\boldsymbol{+R (16)}$$

The dimensions of **D**, **C**, **S**, and residual matrix **R** in the above equation are **m×n**, **m×r**, **n×r**, and **m×n**, respectively.^13^ Eq. (16) can be solved by iterative solving of alternating least squares (ALS) with sequential approximations of concentration and spectral profiles under apply a series of constraints until a convergence criterion is attained. The convergence criterion can be a preset number of iterations or a threshold value defining the difference in fit improvement between consecutive iterations. Initial estimations of concentration and spectral profiles can be created by evolving factor analysis (EFA) and simple-to-use interactive self-modeling mixture analysis (SIMPLISMA), respectively. Among the most well-known constraints non-negativity, unimodality, known pure profiles, closure, and local rank/selectivity can be referred. The accuracy of MCR-ALS is measured by the parameter of lack of fit (**LOF**; can be expressed by Eq. (17)):

$$\boldsymbol{\%LOF=100}\sqrt{\frac{\sum_{\boldsymbol{i,j}} \boldsymbol{e}_{\boldsymbol{ij}}^{\boldsymbol{2}}}{\sum_{\boldsymbol{i,j}} \boldsymbol{d}_{\boldsymbol{ij}}^{\boldsymbol{2}}}}\boldsymbol{(17)}$$

In which $\mathbf{d}_{\mathbf{ij}}$ is an element of the data matrix **D** and $\mathbf{e}_{\mathbf{ij}}$ is the residuals. Ideally, **%LOF** must be as small as possible.^14,15^ Flowchart S2 displays an illustrative diagram of the MCR-ALS model.

Data matrix

**D [m**$\boldsymbol{\times}$**n]**

PCA or

SVA

Number of components **r**

Initial estimation

**C** **[m**$\boldsymbol{\times}$**r]**

Pure concentration

**C [m**$\boldsymbol{\times}$**r]**

Least squares (LS)

$$\mathbf{S=}{\mathbf{(}\mathbf{C}^{\mathbf{T}}\mathbf{C)}}^{\mathbf{-1}}\mathbf{C}^{\mathbf{T}}\mathbf{D}$$

Pure spectra

**S [r**$\boldsymbol{\times}$**n]**

Least squares (LS)

$$\mathbf{C}^{\mathbf{T}}\mathbf{=}{\mathbf{(}\mathbf{S}^{\mathbf{T}}\mathbf{S)}}^{\mathbf{-1}}\mathbf{SD}^{\mathbf{T}}$$

Concentration constraints:

1. Non-negativity

2. Uni-modality

3. Known pure profiles

4. …

Concentration constraints:

1. Non-negativity

2. Closure

3. Local rank/selectivity

4. Known pure profiles

5. …

Data matrix

decomposition

according to bilinear model

**Flowchart S2.** An illustrative diagram of the MCR-ALS model.

**Hard-Soft Modeling: Rank Annihilation Factor Analysis.** Rank annihilation factor analysis (RAFA) is a chemometrics hard-soft model based on rank analysis for two-way data sets. It can be used in solving many chemical problems such as spectrophotometric studies of chemical kinetics, complexation equilibria among cations and chromogenic reagents, protonation equilibria of indicators to estimate parameters such as the rate/stability/acidity constants and also, to obtain pure concentration/spectral profiles.^16-18^ In addition, RAFA has been employed to quantification of targets in systems with unknown background.^19^ The basics of RAFA is on the rank analysis by removing the contribution of a chemical species (pure target) of data set to reduce the rank by one. The lowest value is detected in the residual standard deviation (R.S.D.) plots showing the optimum values of parameters.

In the current study, we use RAFA for the estimation of the association constants of Zn^II^, Cu^II,^ and Fe^II^ complexes with MTB. The principals of the application of RAFA in the spectrophotometric estimation of association constants were described in two previously reported works.^20,21^ In our study, three two-way data sets can be produced by recording absorbance spectra at a series of cation to MTB molar ratios per constant analytical concentrations of the MTB ($\mathbf{C}_{\mathbf{MTB}}$). Removing the contribution of one chemical species (here, MTB) from the data set by RAFA, the rank of the residual data set decreases by one. By substitution of different values of association constants for a given amount of $\mathbf{C}_{\mathbf{Cation}}$ and $\mathbf{C}_{\mathbf{MTB}}$, the different concentration profiles of MTB will be achieved. The true concentration profile will be achieved by substitution of the true values of association constants. The molar absorptivity ($\boldsymbol{\varepsilon}$) of MTB can be achieved from the pure spectrum of MTB. Thus, the true absorption spectra for the MTB at different cation-MTB molar ratios are achieved by multiplying the concentration profile of the MTB by $\boldsymbol{\varepsilon}$. By removing the MTB spectra from the data set, the rank of the residual data set reduces by one.

Here, based on the involved mechanisms of the complex formation systems, the chemical equilibria for the consecutive complex formation between Zn^II^/or Cu^II^ and MTB with 1:2 ($\mathbf{Zn(MTB)}_{\mathbf{2}}$/or $\mathbf{Cu(MTB)}_{\mathbf{2}}$) and 1:1 ($\mathbf{Zn(MTB)}$/or $\mathbf{Cu(MTB)}$) stoichiometries can be described by the following equations:

$$\boldsymbol{Zn+MTB\to Zn(MTB)}\left( \boldsymbol{18} \right)$$

$$\boldsymbol{Zn(MTB)+MTB\to}\boldsymbol{Zn(MTB)}_{\boldsymbol{2}}\boldsymbol{(19)}$$

$$\boldsymbol{K}_{\boldsymbol{a}\boldsymbol{1}}\boldsymbol{=}\frac{\boldsymbol{[Zn(MTB)]}}{\left[ \boldsymbol{Zn} \right]\boldsymbol{[MTB]}}\boldsymbol{(20)}$$

$$\boldsymbol{K}_{\boldsymbol{a}\boldsymbol{2}}\boldsymbol{=}\frac{\boldsymbol{[}\boldsymbol{Zn(MTB)}_{\boldsymbol{2}}\boldsymbol{]}}{\left[ \boldsymbol{Zn(MTB)} \right]\boldsymbol{[MTB]}}\boldsymbol{(21)}$$

$$\boldsymbol{C}_{\boldsymbol{Zn}}\boldsymbol{=}\left[ \boldsymbol{Zn} \right]\boldsymbol{+}\left[ \boldsymbol{Zn(MTB)} \right]\boldsymbol{+}\left[ \boldsymbol{Zn(MTB)}_{\boldsymbol{2}} \right]\boldsymbol{(22)}$$

$$\boldsymbol{C}_{\boldsymbol{MTB}}\boldsymbol{=}\left[ \boldsymbol{MTB} \right]\boldsymbol{+}\left[ \boldsymbol{Zn(MTB)} \right]\boldsymbol{+2}\left[ \boldsymbol{Zn(MTB)}_{\boldsymbol{2}} \right]\boldsymbol{(23)}$$

Where $\mathbf{K}_{\mathbf{a1}}$ and $\mathbf{K}_{\mathbf{a2}}$ are the association constants of $\mathbf{Zn(MTB)}$ and $\mathbf{Zn(MTB)}_{\mathbf{2}}$ complexes, respectively. $\mathbf{[MTB]}$, $\left[ \mathbf{Zn} \right]$**,** **[Zn(MTB)]** and **[Zn(MTB)_2_]** are the equilibrium concentrations of **MTB**, **Zn^II^** cation and $\mathbf{Zn(MTB)}$ **and** $\mathbf{Zn(MTB)}_{\mathbf{2}}$ complexes, respectively. The $\mathbf{C}_{\mathbf{Zn}}$ and $\mathbf{C}_{\mathbf{MTB}}$ are the total Zn^II^ and MTB concentrations, respectively. Readjustments of Eqs. (20), (21) and (22) for $\left[ \mathbf{Zn} \right]$, $\mathbf{Zn(MTB)}$ and $\mathbf{Zn(MTB)}_{\mathbf{2}}$ give the Eqs. (24), (25) and (26), respectively:

$$\left[ \boldsymbol{Zn} \right]\boldsymbol{=}\frac{\boldsymbol{C}_{\boldsymbol{Zn}}}{\boldsymbol{1+}\boldsymbol{K}_{\boldsymbol{a}\boldsymbol{1}}\left[ \boldsymbol{MTB} \right]\boldsymbol{+}\boldsymbol{K}_{\boldsymbol{a}\boldsymbol{1}}\boldsymbol{K}_{\boldsymbol{a}\boldsymbol{2}}\boldsymbol{[MTB]}^{\boldsymbol{2}}}\boldsymbol{(24)}$$

$$\left[ \boldsymbol{Zn(MTB)} \right]\boldsymbol{=}\frac{\boldsymbol{K}_{\boldsymbol{a}\boldsymbol{1}}\boldsymbol{C}_{\boldsymbol{Zn}}\boldsymbol{[MTB]}}{\boldsymbol{1+}\boldsymbol{K}_{\boldsymbol{a}\boldsymbol{1}}\left[ \boldsymbol{MTB} \right]\boldsymbol{+}\boldsymbol{K}_{\boldsymbol{a}\boldsymbol{1}}\boldsymbol{K}_{\boldsymbol{a}\boldsymbol{2}}\boldsymbol{[MTB]}^{\boldsymbol{2}}}\boldsymbol{(25)}$$

$$\left[ \boldsymbol{Zn(MTB)}_{\boldsymbol{2}} \right]\boldsymbol{=}\frac{\boldsymbol{2}\boldsymbol{K}_{\boldsymbol{a}\boldsymbol{1}}\boldsymbol{K}_{\boldsymbol{a}\boldsymbol{2}}\boldsymbol{C}_{\boldsymbol{Zn}}\boldsymbol{[MTB]}^{\boldsymbol{2}}}{\boldsymbol{1+}\boldsymbol{K}_{\boldsymbol{a}\boldsymbol{1}}\left[ \boldsymbol{MTB} \right]\boldsymbol{+}\boldsymbol{K}_{\boldsymbol{a}\boldsymbol{1}}\boldsymbol{K}_{\boldsymbol{a}\boldsymbol{2}}\boldsymbol{[MTB]}^{\boldsymbol{2}}}\boldsymbol{(26)}$$

Substituting $\left[ \mathbf{Zn} \right]$, $\mathbf{Zn(MTB)}$ and $\mathbf{Zn(MTB)}_{\mathbf{2}}$ obtained from Eqs. (24), (25) and (26) into Eq. (23) gives Eq. (27) and finally, solving for $\left[ \mathbf{MTB} \right]$ gives Eq. (28).

$$\boldsymbol{C}_{\boldsymbol{MTB}}\boldsymbol{=}\left[ \boldsymbol{MTB} \right]\boldsymbol{+}\frac{\boldsymbol{K}_{\boldsymbol{a}\boldsymbol{1}}\boldsymbol{C}_{\boldsymbol{Zn}}\boldsymbol{[MTB]}}{\boldsymbol{1+}\boldsymbol{K}_{\boldsymbol{a}\boldsymbol{1}}\left[ \boldsymbol{MTB} \right]\boldsymbol{+}\boldsymbol{K}_{\boldsymbol{a}\boldsymbol{1}}\boldsymbol{K}_{\boldsymbol{a}\boldsymbol{2}}\boldsymbol{[MTB]}^{\boldsymbol{2}}}\boldsymbol{+}\frac{\boldsymbol{2}\boldsymbol{K}_{\boldsymbol{a}\boldsymbol{1}}\boldsymbol{K}_{\boldsymbol{f}\boldsymbol{2}}\boldsymbol{C}_{\boldsymbol{Zn}}\boldsymbol{[MTB]}^{\boldsymbol{2}}}{\boldsymbol{1+}\boldsymbol{K}_{\boldsymbol{a}\boldsymbol{1}}\left[ \boldsymbol{MTB} \right]\boldsymbol{+}\boldsymbol{K}_{\boldsymbol{a}\boldsymbol{1}}\boldsymbol{K}_{\boldsymbol{a}\boldsymbol{2}}\boldsymbol{[MTB]}^{\boldsymbol{2}}}\boldsymbol{(27)}$$

$$\boldsymbol{K}_{\boldsymbol{a}\boldsymbol{1}}\boldsymbol{K}_{\boldsymbol{a}\boldsymbol{2}}\boldsymbol{[MTB]}^{\boldsymbol{3}}\boldsymbol{+}\left( \boldsymbol{K}_{\boldsymbol{a}\boldsymbol{1}}\left( \boldsymbol{1+}\boldsymbol{K}_{\boldsymbol{a}\boldsymbol{2}}\left( \boldsymbol{2}\boldsymbol{C}_{\boldsymbol{Zn}}\boldsymbol{-}\boldsymbol{C}_{\boldsymbol{MTB}} \right) \right) \right)\boldsymbol{[L]}^{\boldsymbol{2}}\boldsymbol{+}\left( \boldsymbol{1+}\boldsymbol{K}_{\boldsymbol{a}\boldsymbol{1}}\left( \boldsymbol{C}_{\boldsymbol{Zn}}\boldsymbol{-}\boldsymbol{C}_{\boldsymbol{MTB}} \right) \right)\left[ \boldsymbol{MTB} \right]\boldsymbol{-}\boldsymbol{C}_{\boldsymbol{MTB}}\boldsymbol{=0 (28)}$$

For a certain value of $\mathbf{C}_{\mathbf{Zn}}$ and $\mathbf{C}_{\mathbf{MTB}}$ and different values for $\mathbf{K}_{\mathbf{a1}}$ and $\mathbf{K}_{\mathbf{a2}}$ solving the polynomial Eq. (28) for $\left[ \mathbf{MTB} \right]$ will be possible. This makes the different concentration vectors for $\left[ \mathbf{MTB} \right]$. Consequently, the true concentration vector will be obtained by replacement the true $\mathbf{K}_{\mathbf{a1}}$ and $\mathbf{K}_{\mathbf{a2}}$ values. The spectrum of the pure MTB can be used to calculate $\boldsymbol{\varepsilon}$. Thus, the absorption spectra for MTB at different values of Zn^II^ to MTB are achieved via multiplying its concentration profiles by removing the MTB contribution from the data set, the rank reduces by one. RAFA utilizes an iterative process to estimation the best $\mathbf{K}_{\mathbf{a}}$ values. The convergence is established by calculation of the $\mathbf{R.S.D.}$values; expressed as:

$$\boldsymbol{R.S.D.}\left( \boldsymbol{n} \right)\boldsymbol{=}\left( \frac{\sum_{\boldsymbol{i=n+1}}^{\boldsymbol{n}} \boldsymbol{g}_{\boldsymbol{i}}}{\boldsymbol{n(c-1)}} \right)^{\frac{\boldsymbol{1}}{\boldsymbol{2}}}\boldsymbol{(29)}$$

In which, n, $g_{i}$ and c are the number of active chemical species, eigenvalue, and the number of samples, respectively. The value of **R.S.D.** of the residual data set after deducting the MTB contribution represents the $\mathbf{K}_{\mathbf{a1}}$ and $\mathbf{K}_{\mathbf{a2}}$ optimum values.

In the current study, the chemical equilibria for the consecutive complex formation between Fe^II^ and MTB with 1:1 ($\mathbf{Fe}(\mathbf{MTB})$) and 2:1 ($\mathbf{Fe}_{\mathbf{2}}(\mathbf{MTB})$) stoichiometries can be described using the following relationships,

$$\boldsymbol{Fe+MTB\to Fe(MTB)}\left( \boldsymbol{30} \right)$$

$$\boldsymbol{Fe(MTB)+Fe\to}\boldsymbol{Fe}_{\boldsymbol{2}}\boldsymbol{(MTB) (31)}$$

$$\boldsymbol{K}_{\boldsymbol{a}\boldsymbol{1}}\boldsymbol{=}\frac{\boldsymbol{[Fe(MTB)]}}{\left[ \boldsymbol{Fe} \right]\boldsymbol{[MTB]}}\boldsymbol{(32)}$$

$$\boldsymbol{K}_{\boldsymbol{a}\boldsymbol{2}}\boldsymbol{=}\frac{\boldsymbol{[}\boldsymbol{Fe}_{\boldsymbol{2}}\boldsymbol{(MTB)]}}{\left[ \boldsymbol{Fe(MTB)} \right]\boldsymbol{[MTB]}}\boldsymbol{(33)}$$

$$\boldsymbol{C}_{\boldsymbol{Fe}}\boldsymbol{=}\left[ \boldsymbol{Fe} \right]\boldsymbol{+}\left[ \boldsymbol{Fe(MTB)} \right]\boldsymbol{+2}\left[ \boldsymbol{Fe}_{\boldsymbol{2}}\boldsymbol{(MTB)} \right] \left( \boldsymbol{34} \right)$$

$$\boldsymbol{C}_{\boldsymbol{MTB}}\boldsymbol{=}\left[ \boldsymbol{MTB} \right]\boldsymbol{+}\left[ \boldsymbol{Fe(MTB)} \right]\boldsymbol{+}\left[ \boldsymbol{Fe}_{\boldsymbol{2}}\boldsymbol{(MTB)} \right]\boldsymbol{(35)}$$

Readjustment of the Eq. (34) for $\left[ \mathbf{Fe}_{\mathbf{2}}\mathbf{(MTB)} \right]$ gives:

$$\boldsymbol{[Fe}_{\boldsymbol{2}}\boldsymbol{(MTB)]=}\frac{\boldsymbol{C}_{\boldsymbol{Fe}}\boldsymbol{-}\left[ \boldsymbol{Fe} \right]\boldsymbol{-}\left[ \boldsymbol{Fe(MTB)} \right]}{\boldsymbol{2}}\boldsymbol{(36)}$$

By substituting of $\left[ \mathbf{Fe}_{\mathbf{2}}\mathbf{(MTB)} \right]$ from Eq. (36) to Eq. (35) and readjustment for $\left[ \mathbf{Fe} \right]$ gives:

$$\left[ \boldsymbol{Fe} \right]\boldsymbol{=}\frac{{\boldsymbol{2}\boldsymbol{C}}_{\boldsymbol{MTB}}\boldsymbol{-2}\left[ \boldsymbol{MTB} \right]\boldsymbol{-}\boldsymbol{C}_{\boldsymbol{Fe}}}{\boldsymbol{K}_{\boldsymbol{a}\boldsymbol{1}}\left[ \boldsymbol{MTB} \right]\boldsymbol{-1}} \left( \boldsymbol{37} \right)$$

By substituting of $\left[ \mathbf{Fe(MTB)} \right]$, $\left[ \mathbf{Fe}_{\mathbf{2}}\mathbf{(MTB)} \right]$ and $\left[ \mathbf{Fe} \right]$ from Eqs. (32), (36) and (37) into Eq. (35):

$$\boldsymbol{C}_{\boldsymbol{MTB}}\boldsymbol{=}\left[ \boldsymbol{MTB} \right]\boldsymbol{+}\frac{\boldsymbol{K}_{\boldsymbol{a}\boldsymbol{1}}\boldsymbol{C}_{\boldsymbol{Fe}}\left[ \boldsymbol{MTB} \right]}{\boldsymbol{1+}\boldsymbol{K}_{\boldsymbol{a}\boldsymbol{1}}\left[ \boldsymbol{MTB} \right]\boldsymbol{+2}\boldsymbol{K}_{\boldsymbol{a}\boldsymbol{1}}\boldsymbol{K}_{\boldsymbol{a}\boldsymbol{2}}\left[ \boldsymbol{Fe} \right]\left[ \boldsymbol{MTB} \right]}\boldsymbol{+}\frac{\boldsymbol{2}\boldsymbol{K}_{\boldsymbol{a}\boldsymbol{1}}\boldsymbol{K}_{\boldsymbol{a}\boldsymbol{2}}\boldsymbol{C}_{\boldsymbol{Fe}}\left[ \boldsymbol{Fe} \right]\left[ \boldsymbol{MTB} \right]}{\boldsymbol{1+}\boldsymbol{K}_{\boldsymbol{a}\boldsymbol{1}}\left[ \boldsymbol{MTB} \right]\boldsymbol{+2}\boldsymbol{K}_{\boldsymbol{a}\boldsymbol{1}}\boldsymbol{K}_{\boldsymbol{a}\boldsymbol{2}}\left[ \boldsymbol{Fe} \right]\left[ \boldsymbol{MTB} \right]} \left( \boldsymbol{38} \right)$$

Finally, readjustment as a function of $\left[ \mathbf{MTB} \right]$ gives:

$$\left( \boldsymbol{K}_{\boldsymbol{a}\boldsymbol{1}}^{\boldsymbol{2}}\boldsymbol{-4}\boldsymbol{K}_{\boldsymbol{a}\boldsymbol{1}}\boldsymbol{K}_{\boldsymbol{a}\boldsymbol{2}} \right)\boldsymbol{[MTB]}^{\boldsymbol{3}}\boldsymbol{+}\left( \boldsymbol{K}_{\boldsymbol{a}\boldsymbol{1}}^{\boldsymbol{2}}\boldsymbol{C}_{\boldsymbol{Fe}}\boldsymbol{-}\boldsymbol{K}_{\boldsymbol{a}\boldsymbol{1}}^{\boldsymbol{2}}\boldsymbol{C}_{\boldsymbol{MTB}}\boldsymbol{+8}\boldsymbol{K}_{\boldsymbol{a}\boldsymbol{1}}\boldsymbol{K}_{\boldsymbol{a}\boldsymbol{2}}\boldsymbol{C}_{\boldsymbol{MTB}}\boldsymbol{-6}\boldsymbol{K}_{\boldsymbol{a}\boldsymbol{1}}\boldsymbol{K}_{\boldsymbol{a}\boldsymbol{2}}\boldsymbol{C}_{\boldsymbol{Fe}} \right)\boldsymbol{[MTB]}^{\boldsymbol{2}}\boldsymbol{-}\left( \boldsymbol{K}_{\boldsymbol{a}\boldsymbol{1}}\boldsymbol{C}_{\boldsymbol{Fe}}\boldsymbol{+2}\boldsymbol{K}_{\boldsymbol{a}\boldsymbol{1}}\boldsymbol{K}_{\boldsymbol{a}\boldsymbol{2}}\left( \boldsymbol{2}\boldsymbol{C}_{\boldsymbol{MTB}}^{\boldsymbol{2}}\boldsymbol{-C}_{\boldsymbol{Fe}}^{\boldsymbol{2}} \right)\boldsymbol{-6}\boldsymbol{K}_{\boldsymbol{a}\boldsymbol{1}}\boldsymbol{K}_{\boldsymbol{a}\boldsymbol{2}}\boldsymbol{C}_{\boldsymbol{Fe}}\boldsymbol{C}_{\boldsymbol{MTB}}\boldsymbol{+1} \right)\left[ \boldsymbol{MTB} \right]\boldsymbol{+}\boldsymbol{C}_{\boldsymbol{MTB}}\boldsymbol{=0.0 (39)}$$

Similar to the Zn^II^/or Cu^II^ case, by removing the MTB contribution from the original data set the true values of association constants are accessible.

**Figure S2.** Involved species during MTB acidity equilibria.^22,23^

1. MTB_H4_


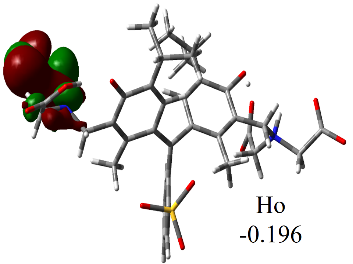

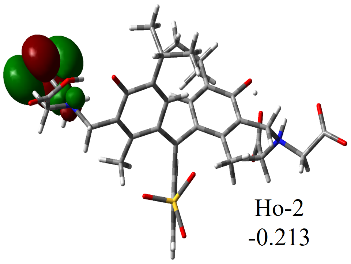

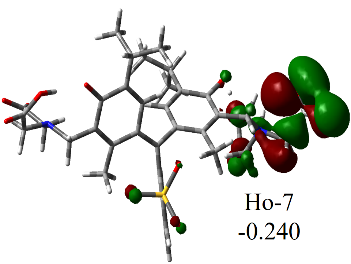

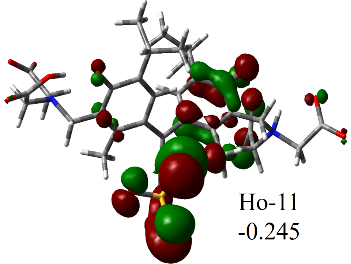


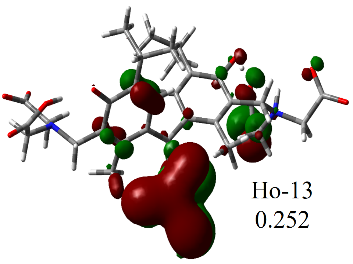

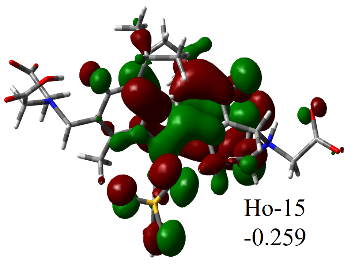

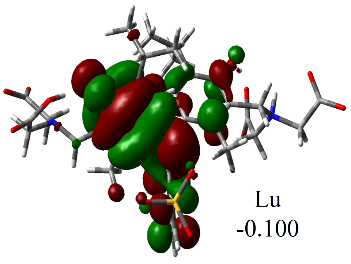

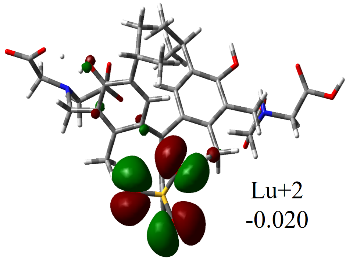


1. MTB_H6_


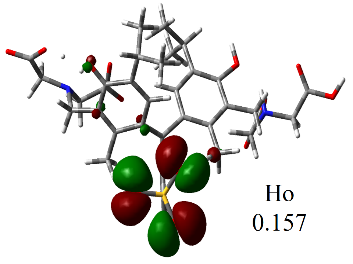

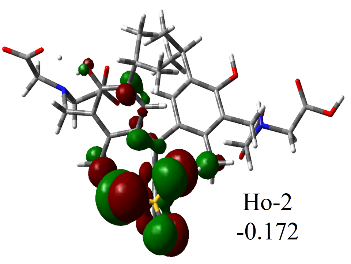

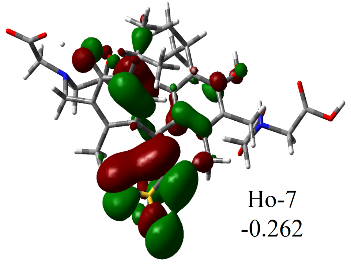

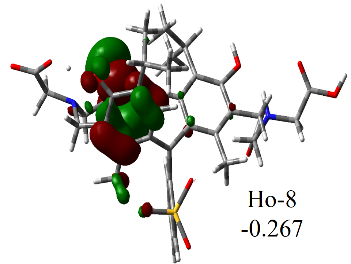


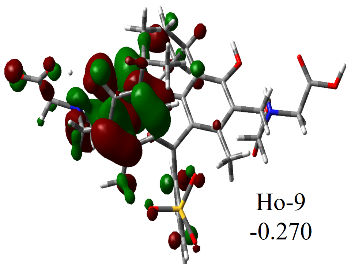

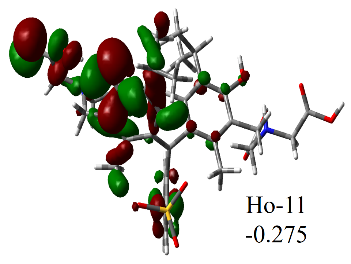

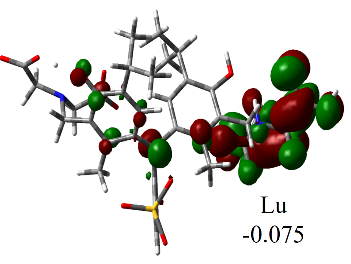

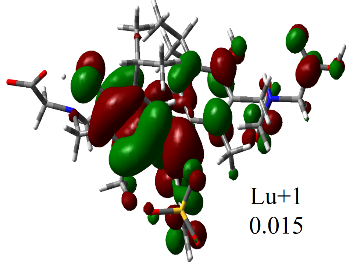


1. MTB_H0_


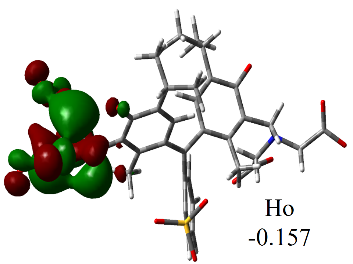

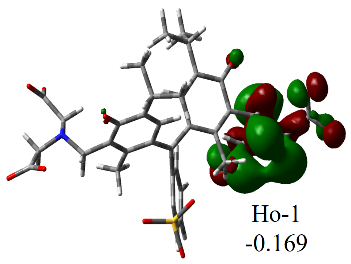

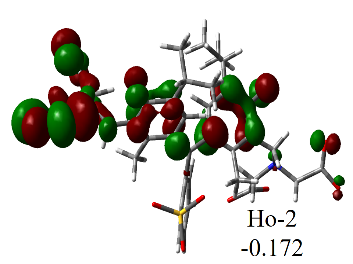

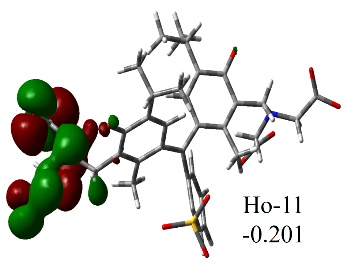


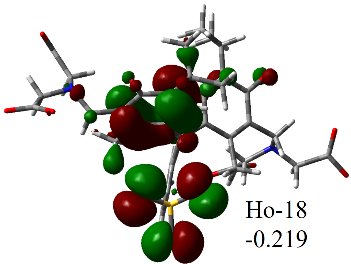

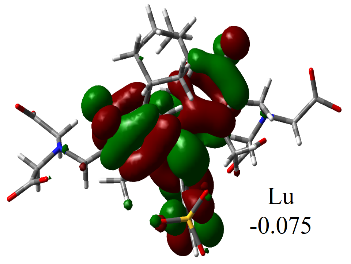

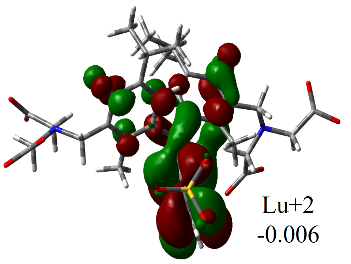

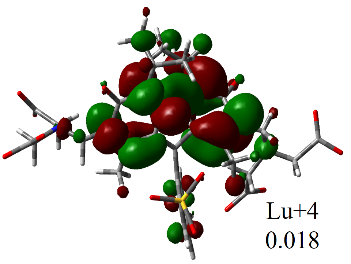


**Figure S3.** Representation of MOs involved in the major electronic transitions for (a) MTB_H4_, (b) MTB_H6_ and (c) MTB_H0_


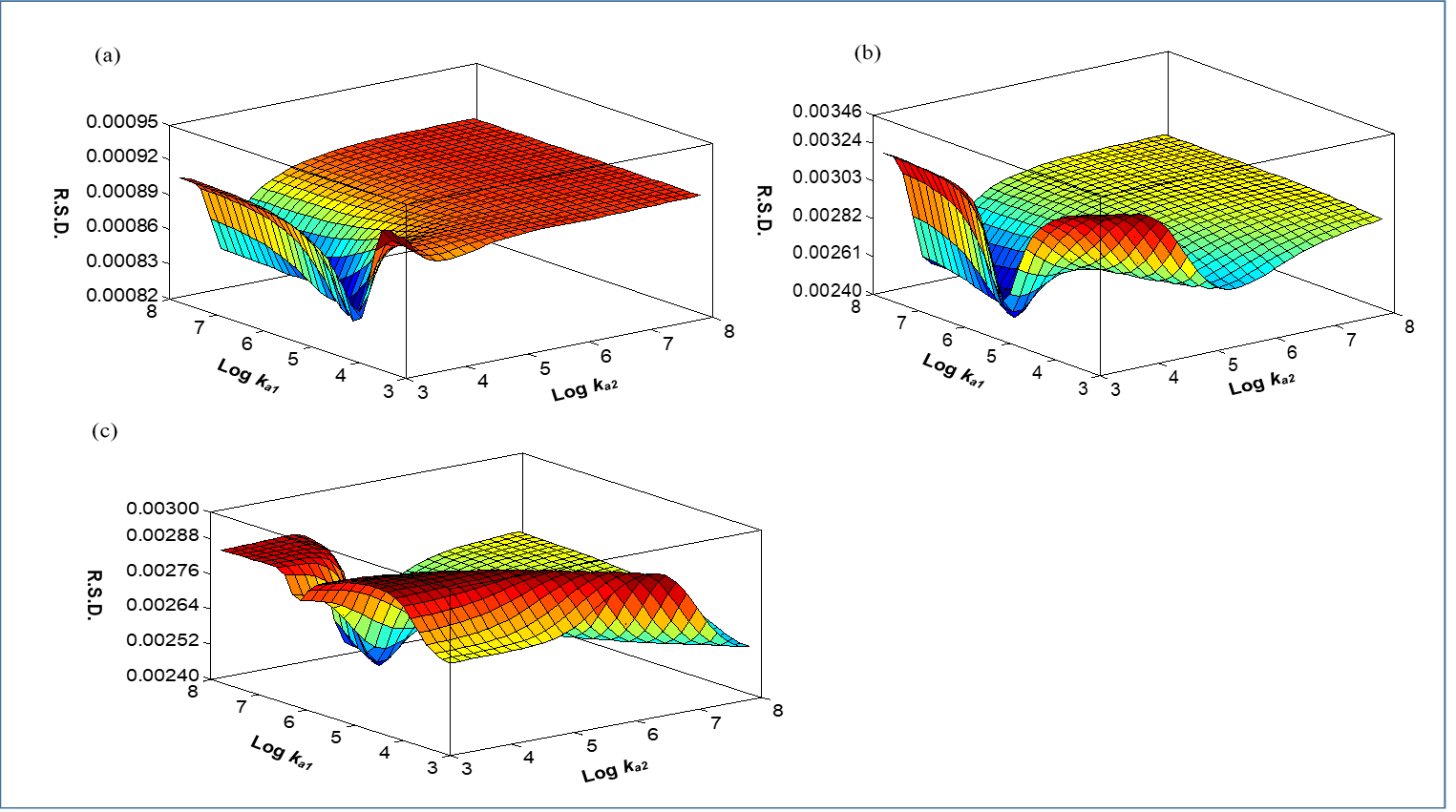


**Figure S4.** Relationship between the R.S.D. and association constants for (a) Zn^II^, (b) Cu^II^, and (c) Fe^II^ complexes.

.

**Figure S5.** Hypothetical molecular structures (HMSs) used in the computations for the Zn(MTB), Cu(MTB) and Fe(MTB) complexes.

**Cont. Figure S5.** Hypothetical molecular structures (HMSs) used in the computations for the Zn(MTB), Cu(MTB) and Fe(MTB) complexes.

**Con. Figure S5.** Hypothetical molecular structures (HMSs) used in the computations for the Zn(MTB), Cu(MTB) and Fe(MTB) complexes.

**Cont. Figure S5.** Hypothetical molecular structures (HMSs) used in the computations for the Zn(MTB), Cu(MTB) and Fe(MTB) complexes.

**Figure S6.** Molecular structure of MTB_H4_, symbols show the involved moieties and metal ions in coordination spheres in the complexes.

**Table S1.** Bond lengths (Å) and angles (°) for the optimized complexes of Zn(MTB) with Oh, SP, and Td geometries in HMSs 1 to 43 in Figure S5. The atom names are indicated in Figure S6. The number in parentheses indicates the number of water molecules involved in the coordination.

| Metal ion | Geometry | HMS | Bond lengths (Å) | | | | | | | | Angles (°) | | | | | | | | | | | | | | | | | | Pref. geometry |
| --- | --- | --- | --- | --- | --- | --- | --- | --- | --- | --- | --- | --- | --- | --- | --- | --- | --- | --- | --- | --- | --- | --- | --- | --- | --- | --- | --- | --- | --- |
|  |  |  | MI-N | | | MI-Ohy | | | MI-Oca_1_ | | N-MI-Ohy | | | | | | | N-MI-Oca_1_ | | | | Ohy-MI-Oca_1_ | | | | | | |  |
| Zn^II^ | Oh | 1(4) | - | | | 2.07 | | | 2.17 | | - | | | | | | | - | | | | 99.05 | | | | | | | Oh(4) |
|  |  | 2(4) | 2.28 | | | 2.05 | | | 2.12 | | 81.16 | | | | | | | 78.15 | | | | 150.12 | | | | | | | Oh(3) |
|  |  | 3(4) | - | | | 2.08 | | | 2.13 | | - | | | | | | | - | | | | 93.06 | | | | | | | SPY(3) |
|  |  | 4(4) | 2.28 | | | 1.99 | | | 2.15 | | 82.05 | | | | | | | 76.89 | | | | 148.97 | | | | | | | Oh(3) |
|  |  | 5(4) | - | | | 2.08 | | | 2.12 | | - | | | | | | | - | | | | 92.88 | | | | | | | SPY(3) |
|  |  | 6(4) | 2.26 | | | 1.98 | | | 2.14 | | 82.80 | | | | | | | 76.43 | | | | 142.39 | | | | | | | SPY(3) |
|  |  | 7(4) | - | | | 2.05 | | | 2.08 | | - | | | | | | | - | | | | 98.46 | | | | | | | SPY(3) |
|  |  | 8(4) | 2.21 | | | 2.08 | | | 2.10 | | 80.87 | | | | | | | 81.89 | | | | 158.42 | | | | | | | Oh(3) |
|  |  |  | MI-O_Ca1_ | | | | MI-O_Ca2_ | | | | Oca_1_-MI-Oca_2_ | | | | | | | | | | | | | | | | | |  |
|  |  | 9(4) | 2.15 | | | | 2.04 | | | | 91.78 | | | | | | | | | | | | | | | | | | Oh(4) |
|  |  | 10(4) | 2.09 | | | | 2.07 | | | | 99.58 | | | | | | | | | | | | | | | | | | Oh(4) |
|  |  | 13(4) | 2.15 | | | | 2.04 | | | | 91.78 | | | | | | | | | | | | | | | | | | Oh(4) |
|  |  | 14(4) | 2.07 | | | | 2.09 | | | | 99.58 | | | | | | | | | | | | | | | | | | Oh(4) |
|  |  |  | MI-N | MI-Ohy | | | MI-O_Ca1_ | | MI-O_Ca2_ | | N-MI-Ohy | Ohy-MI-Oca_1_ | | | Ohy-MI-Oca_2_ | | | | Oca_1_-MI-Oca_2_ | | | | N-MI-Oca_1_ | | | | | N-MI-Oca_2_ |  |
|  |  | 20(3) | - | 2.06 | | | - | | 2.14 | | - | 114.92 | | | 89.28 | | | | 77.66 | | | | | | - | | | - | SPY(3) |
|  |  | 21(3) | 2.21 | 2.03 | | | 2.17 | | 2.19 | | 83.84 | 158.79 | | | 91.54 | | | | 97.77 | | | | | | 79.63 | | | 77.71 | Oh(1) |
|  |  | 22(3) | - | 2.01 | | | 2.04 | | - | | - | 111.58 | | | - | | | | - | | | | | | - | | | - | SPY(2) |
|  |  | 23(3) | 2.22 | 2.05 | | | 2.25 | | 2.15 | | 90.74 | 158.72 | | | 95.63 | | | | 97.81 | | | | | | 75.50 | | | 80.00 | Oh(2) |
|  |  | 25(3) | 2.22 | 2.05 | | | 2.26 | | 2.15 | | 90.84 | 158.41 | | | 96.09 | | | | 97.52 | | | | | | 75.19 | | | 79.94 | Oh(2) |
|  |  |  | MI-N | | | | | MI-Ohy | | | N-MI-Ohy | | | | | | | | | | | | | | | | | |  |
|  |  | 27(4) | 2.27 | | | | | 2.04 | | | 83.06 | | | | | | | | | | | | | | | | | | Oh(4) |
|  |  | 28(4) | 2.27 | | | | | 2.04 | | | 82.95 | | | | | | | | | | | | | | | | | | Oh(4) |
|  |  | 29(4) | 2.25 | | | | | 2.02 | | | 84.35 | | | | | | | | | | | | | | | | | | Oh(4) |
|  |  |  | MI-N | | MI-Ohy | | | MI-Oca_1_ | | MI-Oca_2_ | N-MI-Ohy | | Ohy-MI-Oca_1_ | | | | Ohy-MI-Oca_2_ | | | | Oca_1_-MI-Oca_2_ | | | | | N-MI-Oca_1_ | | N-MI-Oca_2_ |  |
|  |  | 34(3) | 2.27 | | - | | | 2.12 | | 2.07 | - | | - | | | | - | | | | 104.35 | | | | | 80.43 | | 78.12 | Oh(3) |
|  |  | 35(3) | 2.24 | | 2.13 | | | 2.14 | | - | 79.40 | | 160.34 | | | |  | | | | - | | | | | 80.99 | | - | Oh(3) |
|  |  |  | MI-N | | MI-Ohy | | | MI-Oca_1_ | | MI-Oca_2_ | N-MI-Ohy | | | | | | | Ohy-MI-Oca_2_ | | | | N-MI-Oca_2_ | | | | | | | SPY(2) |
|  |  | 37(3) | 2.13 | | 2.07 | | |  | | 2.01 | 87.24 | | | | | | | 103.01 | | | | 83.41 | | | | | | | SPY(2) |
|  |  | 38(3) | 2.13 | | 2.05 | | | 2.03 | | - | 88.30 | | | | | | | 103.40 | | | | 83.10 | | | | | | | SPY(2) |
|  |  | 39(3) | 2.14 | | 2.11 | | | - | | 2.00 | 85.91 | | | | | | | 101.57 | | | | 83.39 | | | | | | |  |
|  |  |  | MI-N | | MI-Ohy | | | MI-Oca_1_ | | MI-Oca_2_ | N-MI-Ohy | | | Ohy-MI-Oca_1_ | | Ohy-MI-Oca_2_ | | | | Oca_1_-MI-Oca_2_ | | | | N-MI-Oca_1_ | | | N-MI-Oca_2_ | |  |
|  |  | 42(2) | 2.22 | | 2.00 | | | 2.03 | | 2.07 | 90.11 | | | 134.15 | | 123.15 | | | | 100.27 | | | | 83.24 | | | 79.83 | | TBP(1) |
| Zn^II^ | SP |  | MI-N | | | MI-Ohy | | | MI-O_Ca1_ | | N-MI-Ohy | | | | | | | N-MI-Oca_1_ | | | | Ohy-MI-Oca_1_ | | | | | | |  |
|  |  | 2(2) | 2.12 | | | 2.03 | | | 2.00 | | 86.33 | | | | | | | 83.42 | | | | 161.29 | | | | | | | SP(1) |
|  |  | 4(2) | 2.12 | | | 2.04 | | | 2.02 | | 88.10 | | | | | | | 83.20 | | | | 161.29 | | | | | | | SP(1) |
|  |  | 5(2) | - | | | 2.07 | | | 1.99 | | - | | | | | | | - | | | | 96.00 | | | | | | | SP(2) |
|  |  | 6(2) | 2.12 | | | 1.98 | | | 2.01 | | 87.97 | | | | | | | 83.05 | | | | 159.32 | | | | | | | SP(1) |
|  |  | 8(2) | 2.12 | | | 2.07 | | | 2.03 | | 89.37 | | | | | | | 82.98 | | | | 170.65 | | | | | | | SPY(2) |
|  |  |  | MI-N | | | MI-O_Ca1_ | | | MI-O_Ca2_ | | Oca_1_-MI-Oca_2_ | | | | | | | N-MI-Oca_1_ | | | | N-MI-Oca_2_ | | | | | | |  |
|  |  | 9(2)^a^ | - | | | 2.00 | | | 2.04 | | 93.47 | | | | | | | - | | | | - | | | | | | | SP(2) |
|  |  | 11(2) | - | | | 2.05 | | | 2.06 | | 89.84 | | | | | | | - | | | | - | | | | | | | SP(2) |
|  |  | 17(2) | 2.26 | | | 2.14 | | | 2.01 | | 100.63 | | | | | | | 78.25 | | | | 83.96 | | | | | | | SPY(2) |
|  |  |  | MI-N | | MI-Ohy | | | MI-Oca_1_ | | MI-O_Ca2_ | N-MI-Ohy | | | Ohy-MI-Oca_1_ | | Ohy-MI-Oca_2_ | | | | Oca_1_-MI-Oca_2_ | | | | N-MI-Oca_1_ | | | N-MI-Oca_2_ | |  |
|  |  | 19(1) | 2.18 | | 2.12 | | | 2.13 | | 2.00 | 89.89 | | | 154.27 | | 97.96 | | | | 104.01 | | | | 78.72 | | | 85.69 | | SPY(1) |
|  |  | 21(1) | 2.17 | | 2.05 | | | 2.19 | | 2.02 | 91.51 | | | 157.00 | | 100.98 | | | | 98.43 | | | | 77.78 | | | 85.57 | | SPY(1) |
|  |  | 23(1) | 2.18 | | 2.06 | | | 2.17 | | 2.02 | 91.25 | | | 157.40 | | 99.87 | | | | 99.13 | | | | 78.12 | | | 85.57 | | SPY(1) |
|  |  |  | MI-N | | MI-Ohy | | | MI-Oca_1_ | | MI-Oca_2_ | N-MI-Ohy | | | Ohy-MI-Oca_1_ | | Oph-MI-Oca_2_ | | | | Oca_1_-MI-Oca_2_ | | | | N-MI-Oca_1_ | | | N-MI-Oca_2_ | |  |
|  |  | 30(1) | 2.18 | | 2.08 | | | 2.02 | | 1.96 | 89.76 | | | 112.54 | | 119.21 | | | | 126.48 | | | | 82.94 | | | 84.49 | | SPY(1) |

**Cont.**

|  |  | 31(1) | 2.20 | 2.02 | | 2.04 | | | | 1.98 | 92.02 | | 113.77 | 123.03 | | 121.42 | | 81.95 | | 82.93 | | SPY(1) |
| --- | --- | --- | --- | --- | --- | --- | --- | --- | --- | --- | --- | --- | --- | --- | --- | --- | --- | --- | --- | --- | --- | --- |
|  |  | 32(1) | 2.23 | 2.04 | | 2.04 | | | | 1.98 | 89.04 | | 119.50 | 115.11 | | 122.28 | | 81.03 | | 82.64 | | SPY(1) |
|  |  | 33(1) | 2.19 | 2.00 | | 2.05 | | | | 1.99 | 93.03 | | 114.38 | 125.87 | | 118.42 | | 82.27 | | 83.07 | | SPY(1) |
|  |  | 34(1) | 2.19 | 2.03 | | 2.04 | | | | 1.98 | 91.95 | | 114.82 | 122.10 | | 121.39 | | 82.12 | | 83.16 | | SPY(1) |
|  |  | 35(1) | 2.18 | 2.00 | | 2.05 | | | | 1.99 | 93.17 | | 114.93 | 125.38 | | 118.51 | | 82.54 | | 83.33 | | SPY(1) |
| Zn^II^ | Td |  | MI-N | MI-Ohy | MI-Oca_1_ | | MI-Oca_2_ | | | | N-MI-Ohy | N-MI-Oca_1_ | | N-MI-Oca_2_ | | Ohy-MI-Oca_1_ | | Ohy-MI-Oca_2_ | | Oca_1_-MI-Oca_2_ | |  |
|  |  | 1(2) | - | 2.00 | | 1.96 | | | | - | - | | - | - | | 116.07 | | - | | - | | Td(2) |
|  |  | 2(2) | 2.21 | 2.24 | | 2.09 | | | | 2.10 | 86.96 | | 79.22 | 80.71 | | 156.71 | | 92.15 | |  | | Oh(2) |
|  |  | 3(2) | - | 1.99 | | 1.97 | | | | - | - | | - | - | | 117.07 | | - | | - | | Td(2) |
|  |  | 4(2) | 2.19 | 2.06 | | 2.11 | | | | - | 89.99 | | 79.10 | - | | 162.80 | |  | | - | | SPY(2) |
|  |  | 5(2) | - | 1.99 | | 1.97 | | | | - | - | | - | - | | 117.17 | | - | | - | | Td(2) |
|  |  | 6(2) | 2.19 | 2.07 | | 2.11 | | | | - | 89.70 | | 79.23 | - | | 162.89 | | - | | - | | SPY(2) |
|  |  | 7(2) | - | 2.01 | | 1.98 | | | | - | - | | - | - | | 115.53 | | - | | - | | Td(2) |
|  |  | 8(2) | 2.19 | 2.05 | | 2.12 | | | | - | 90.83 | | 79.12 | - | | 165.50 | | - | | - | | SPY(2) |
|  |  |  | MI-N | MI-Ohy | MI-Oca_1_ | | | MI-Oca_2_ | | | N-MI-Ohy | Ohy-MI-Oca_1_ | | | Ohy-MI-Oca_2_ | | Oca_1_-MI-Oca_2_ | | N-MI-Oca_1_ | | N-MI-Oca_2_ |  |
|  |  | 9(2) | - | - | 2.00 | | | | 1.96 | | - | - | | | - | | 96.07 | | - | | - | Td(2) |
|  |  | 10(2) |  | 2.00 | 2.02 | | | |  | |  |  | | |  | | 117.07 | |  | |  | Td(2) |
|  |  | 13(2) | - | 2.07 | 2.03 | | | | 1.97 | | - | 102.60 | | | 111.90 | | 98.13 | | - | | - | SPY(2) |
|  |  |  | MI-N | MI-Ohy | MI-O_Ca1_ | | MI-O_Ca2_ | | | | N-MI-Ohy | Ohy-MI-Oca_1_ | | Ohy-MI-Oca_1_ | | | Oca_1_-MI-Oca_2_ | | N-MI-Oca_1_ | | N-MI-Oca_2_ |  |
|  |  | 19(1) | 2.18 | 1.98 | 2.04 | | 2.01 | | | | 92.33 | 121.58 | | 120.04 | | | 116.83 | | 80.87 | | 84.20 | SPY(1) |
|  |  | 20(1) | - | 1.91 | 1.96 | | 2.07 | | | | - | 118.79 | | 119.66 | | | 96.57 | | - | | - | Td(1) |
|  |  | 22(1) | - | 1.91 | 1.96 | | 2.07 | | | | - | 118.47 | | 119.26 | | | 96.69 | | - | | - | Td(1) |
|  |  | 23(1) | 2.18 | 1.98 | 2.04 | | 2.01 | | | | 92.33 | 121.57 | | 120.04 | | | 116.83 | | 80.87 | | 84.19 | SPY(1) |
|  |  | 25(1) | 2.17 | 1.93 | 2.05 | | 2.07 | | | | 96.00 | 128.81 | | 123.54 | | | 106.96 | | 81.42 | | 82.88 | SPY(1) |

**Table S2.** Bond lengths (Å) and angles (°) for the optimized complexes of Cu(MTB) with Oh, SP and Td geometries in HMSs 1 to 43 in Figure S5. The atom names are indicated in Figure S6. The number in parentheses indicates the number of water molecules involved in the coordination.

| Metal ion | Geometry | HMS | Bond lengths (Å) | | | | | | Angles (°) | | | | | | | | | | | | | | | | | | | Pref. geometry |
| --- | --- | --- | --- | --- | --- | --- | --- | --- | --- | --- | --- | --- | --- | --- | --- | --- | --- | --- | --- | --- | --- | --- | --- | --- | --- | --- | --- | --- |
|  |  |  | MI-N | | | MI-Ohy | | MI-Oca_1_ | N-MI-Ohy | | | | | | N-MI-Oca_1_ | | | | | | Ohy-MI-Oca_1_ | | | | | | |  |
| Cu^II^ | Oh | 3(4) | - | | | 1.99 | | - | - | | | | | | - | | | | | | - | | | | | | | SPY(4) |
|  |  | 4(4) | 2.09 | | | 1.93 | | 2.00 | 85.45 | | | | | | 84.50 | | | | | | 163.50 | | | | | | | SPY(2) |
|  |  | 5(4) | - | | | 1.99 | | - | - | | | | | | - | | | | | | - | | | | | | | SPY(4) |
|  |  | 6(4) | 2.09 | | | 1.93 | | 2.00 | 85.52 | | | | | | 84.87 | | | | | | 164.48 | | | | | | | SPY(2) |
|  |  | 8(4) | 2.10 | | | 1.93 | | 2.02 | 84.91 | | | | | | 84.12 | | | | | | 164.80 | | | | | | | SPY(2) |
|  |  |  | MI-N | MI-Ohy | | | MI-O_Ca1_ | MI-O_Ca2_ | Oca_1_-MI-Oca_2_ | | | | | | | | | | | | | | | | | | |  |
|  |  | 9(4) | - | - | | | 1.94 | 2.24 | 92.85 | | | | | | | | | | | | | | | | | | | SPY(3) |
|  |  | 10(4) | - | - | | | 1.93 | 2.06 | 89.30 | | | | | | | | | | | | | | | | | | | SPY(3) |
|  |  | 12(4) | - | - | | | - | 2.02 | - | | | | | | | | | | | | | | | | | | | SP(3) |
|  |  | 16(4) |  | - | | | - | 2.02 | - | | | | | | | | | | | | | | | | | | | SP(3) |
|  |  |  | MI-N | MI-Ohy | | | MI-O_Ca1_ | MI-O_Ca2_ | N-MI-Ohy | | | | Ohy-MI-Oca_1_ | | | | Ohy-MI-Oca_2_ | | Oca_1_-MI-Oca_2_ | | | | N-MI-Oca_1_ | | | N-MI-Oca_2_ | |  |
|  |  | 18(3) |  | - | | | 1.93 | 2.06 | - | - | | | | | | | - | | 89.30 | | | | - | | | - | | SPY(3) |
|  |  | 19(3) | 2.25 | 2.06 | | | - | 1.97 | 82.35 | - | | | | | | | 88.50 | | - | | | | - | | | 81.13 | | Oh(3) |
|  |  | 20(3) | - | 1.96 | | | 2.02 | - | - | 88.49 | | | | | | | - | | - | | | | - | | | - | | SPY(3) |
|  |  | 21(3) | 2.19 | 1.96 | | | 1.99 | 2.12 | 84.37 | | 165.40 | | | | | | 90.71 | | 96.10 | | | | 84.20 | | | 79.45 | | SPY(1) |
|  |  | 22(3) | - | - | | | 1.94 | 2.06 | - | | | | - | | | | - | | 88.38 | | | |  | | | - | | SPY(3) |
|  |  | 23(3) | 2.23 | 1.93 | | | 1.88 | 1.97 | 89.98 | | | | 169.02 | | 91.63 | | | | | 92.38 | | 80.28 | | | | | 84.10 | SPY(1) |
|  |  | 25(3) | 2.21 | 1.98 | | | 2.03 | 2.08 | 94.34 | | | | 165.36 | | 90.69 | | | | | 95.32 | | 79.73 | | | | | 82.46 | SPY(1) |
|  |  |  | MI-N | | | | MI-Ohy | | N-MI-Ohy | | | | | | | | | | | | | | | | | | |  |
|  |  | 27(4) | 2.36 | | | | 1.94 | | 80.93 | | | | | | | | | | | | | | | | | | | SPY(2) |
|  |  | 29(4) | 2.36 | | | | 1.94 | | 81.03 | | | | | | | | | | | | | | | | | | | SPY(2) |
|  |  |  | MI-N | MI-Ohy | | | MI-Oca_1_ | MI-Oca_2_ | N-MI-Ohy | | | | | | Ohy-MI-Oca_2_ | | | | | | N-MI-Oca_2_ | | | | | | |  |
|  |  | 36(3) | 2.03 | 2.30 | | | - | 1.95 | 82.54 | | | | | | 91.01 | | | | | | 82.54 | | | | | | | SPY(2) |
|  |  | 37(3) | 2.02 | 2.22 | | |  | 1.97 | 84.28 | | | | | | 94.34 | | | | | | 84.94 | | | | | | | SPY(2) |
|  |  | 38(3) | 2.02 | 2.22 | | |  | 1.97 | 84.52 | | | | | | 94.74 | | | | | | 84.92 | | | | | | | SPY(2) |
|  |  |  | MI-N | MI-Ohy | | | MI-Oca_1_ | MI-Oca_2_ | N-MI-Ohy | | | Ohy-MI-Oca_1_ | | | | | | Ohy-MI-Oca_2_ | | Oca_1_-MI-Oca_2_ | | | | | N-MI-Oca_1_ | | N-MI-Oca_2_ |  |
|  |  | 40(2) ^a^ | 2.06 | 1.93 | | | 2.00 | 2.25 | 94.66 | | | 160.50 | | | 111.34 | | | | 88.10 | | | 86.01 | | | | | 82.66 | SPY(1) |
| Cu^II^ | SP |  | MI-N | | | MI-Ohy | | MI-O_Ca1_ | N-MI-Ohy | | | | | | N-MI-Oca_1_ | | | | | | Ohy-MI-Oca_1_ | | | | | | |  |
|  |  | 2(2) | 2.03 | | | 1.97 | | 1.95 | 87.55 | | | | | | 85.73 | | | | | | 165.74 | | | | | | | SP(1) |
|  |  | 4(2) | 2.03 | | | 1.94 | | 1.96 | 87.84 | | | | | | 86.08 | | | | | | 165.48 | | | | | | | SP(1) |
|  |  | 5(2) | - | | | 1.97 | | 1.94 | - | | | | | | - | | | | | | 96.25 | | | | | | | SP(2) |
|  |  | 6(2) | 2.03 | | | 1.95 | | 1.96 | 87.79 | | | | | | 85.82 | | | | | | 164.90 | | | | | | | SP(1) |
|  |  |  | MI-N | | | MI-O_Ca1_ | | MI-O_Ca2_ | Oca_1_-MI-Oca_2_ | | | | | | N-MI-Oca_1_ | | | | | | N-MI-Oca_2_ | | | | | | |  |
|  |  | 9(2) | - | | | 1.95 | | 1.96 | 92.43 | | | | | | - | | | | | | - | | | | | | | SP(2) |
|  |  | 10(2) | 2.54 | | | 1.97 | | 1.94 | 104.50 | | | | | | 75.10 | | | | | | 77.25 | | | | | | | SPY(2) |
|  |  | 12(2) | 2.50 | | | 1.98 | | 1.94 | 102.31 | | | | | | 75.80 | | | | | | 78.13 | | | | | | | SPY(2) |
|  |  | 13(2) | - | | | 1.94 | | 1.92 | 92.79 | | | | | | - | | | | | | - | | | | | | | SP(2) |
|  |  | 14(2) | 2.46 | | 1.99 | | | 1.94 | 101.34 | | | | | 76.62 | | | | | | | | 78.92 | | | | | | SPY(2) |
|  |  | 15(2) | - | | 1.95 | | | 1.93 | 92.21 | | | | | | - | | | | | | | - | | | | | | SP(2) |
|  |  | 16(2) | 2.42 | | 2.00 | | | 1.96 | 99.08 | | | | | | 77.24 | | | | | | | 80.03 | | | | | | SPY(2) |
|  |  | 17(2) | 2.43 | | 1.98 | | | 1.97 | 80.472 | | | | | | 80.733 | | | | | | | 93.175 | | | | | | SPY(2) |
|  |  |  | MI-N | MI-Ohy | | | MI-Oca_1_ | MI-O_Ca2_ | N-MI-Ohy | | | Ohy-MI-Oca_1_ | | | | | Ohy-MI-Oca_2_ | | | Oca_1_-MI-Oca_2_ | | | | N-MI-Oca_1_ | | | N-MI-Oca_2_ |  |
|  |  | 19(1) | 2.25 | 2.05 | | | 2.01 | 1.96 | 90.38 | | | 162.99 | | | 93.84 | | | | 98.62 | | | 79.74 | | | | | 83.11 | SPY(1) |
|  |  | 21(1) | 2.23 | 1.98 | | | 2.03 | 2.02 | 91.35 | | | 164.41 | | | 96.73 | | | | 79.85 | | | 79.85 | | | | | 82.25 | SPY(1) |
|  |  | 23(1) | 2.23 | 1.99 | | | 2.03 | 2.00 | 91.20 | | | 164.20 | | | 94.67 | | | | 97.08 | | | 79.77 | | | | | 83.25 | SPY(1) |
|  |  | 25(1) |  | 1.97 | | | 1.93 | 1.89 | 88.59 | | | 156.54 | | | 93.41 | | | | 93.66 | | | 72.38 | | | | | 72.25 | SP(1) |
|  |  |  | MI-N | MI-Ohy | | | MI-Oca_1_ | MI-Oca_2_ | N-MI-Ohy | | | Ohy-MI-Oca_1_ | | | | Oph-MI-Oca_2_ | | | Oca_1_-MI-Oca_2_ | | | N-MI-Oca_1_ | | | | | N-MI-Oca_2_ |  |
|  |  | 28(2) | 2.42 |  | | | 2.00 | 1.95 | - | | | - | | | - | | | | 99.27 | | | 77.30 | | | | | 79.80 | SP(2) |

**Cont.**

|  |  | HMS | Bond lengths (Å) | | | | | | | | | Angles (°) | | | | | | | | | | | | | | | | | Pref. geometry |
| --- | --- | --- | --- | --- | --- | --- | --- | --- | --- | --- | --- | --- | --- | --- | --- | --- | --- | --- | --- | --- | --- | --- | --- | --- | --- | --- | --- | --- | --- |
|  |  |  | MI-N | MI-Ohy | | | | MI-Oca_1_ | | MI-Oca_2_ | | N-MI-Ohy | Ohy-MI-Oca_1_ | | | Oph-MI-Oca_2_ | | | | Oca_1_-MI-Oca_2_ | | | | | N-MI-Oca_1_ | | | N-MI-Oca_2_ |  |
|  |  | 30(1) | 2.05 | 2.26 | | | | 1.93 | | 1.94 | | 91.15 | 105.65 | | | 94.31 | | | | 159.25 | | | | | 86.53 | | | 87.47 | SPY(1) |
|  |  | 32(1) | 2.03 | 2.16 | | | | 2.00 | | 1.95 | | 92.06 | 106.64 | | | 115.91 | | | | 137.10 | | | | | 86.04 | | | 87.18 | SPY(1) |
|  |  | 34(1) | 2.03 | 2.20 | | | | 1.99 | | 1.95 | | 93.19 | 105.78 | | | 112.44 | | | | 141.27 | | | | | 85.18 | | | 86.68 | SPY(1) |
|  |  | 35(1) | 2.10 | 2.01 | | | | 2.21 | | 2.00 | | 93.09 | 101.76 | | | 145.10 | | | | 112.14 | | | | | 82.36 | | | 83.69 | SPY(1) |
|  |  |  | MI-N | MI-Ohy | | | | MI-Oca_1_ | | MI-Oca_2_ | | N-MI-Ohy | Ohy-MI-Oca_1_ | | | Ohy-MI-Oca_2_ | | | | Oca_1_-MI-Oca_2_ | | | | | N-MI-Oca_1_ | | | N-MI-Oca_2_ |  |
|  |  | 36(1) | 2.24 | 2.00 | | | | 1.98 | | 1.98 | | 84.42 | 163.52 | | | 90.92 | | | | 99.02 | | | | | 84.30 | | | 80.52 | SPY(1) |
|  |  | 37(1) | 2.21 | 1.95 | | | | 2.00 | | 2.05 | | 84.63 | | 163.33 | | | | 92.15 | | | | 97.64 | | | | 83.75 | | 80.13 | SPY(1) |
|  |  | 38(1) | 2.22 | 1.96 | | | | 1.99 | | 2.04 | | 84.41 | | 162.29 | | | | 92.32 | | | | 98.37 | | | | 83.58 | | 80.05 | SPY(1) |
|  |  | 39(1) | 2.19 | 1.94 | | | | 2.01 | | 2.11 | | 84.63 | | 163.63 | | | | 92.69 | | | | 96.39 | | | | 79.89 | | 83.60 | SPY(1) |
| Cu^II^ | Td |  | MI-N | MI-Ohy | | | | MI-Oca_1_ | | MI-Oca_2_ | | N-MI-Ohy | | | N-MI-Oca_1_ | | N-MI-Oca_2_ | | | | Ohy-MI-Oca_1_ | | | Ohy-MI-Oca_2_ | | | Oca_1_-MI-Oca_2_ | |  |
|  |  | 2(2) | 2.34 | - | | | | 1.98 | | 2.00 | | - | | | - | | - | | | | - | | | - | | | 99.19 | | SPY(2) |
|  |  |  | MI-N | MI-Ohy | | | | MI-Oca_1_ | | MI-Oca_2_ | | N-MI-Ohy | | | Ohy-MI-Oca_1_ | | Ohy-MI-Oca_2_ | | | | Oca_1_-MI-Oca_2_ | | | N-MI-Oca_1_ | | | N-MI-Oca_2_ | |  |
|  |  | 9(2) | - | - | | | | 1.95 | | 1.96 | | - | | | - | | - | | | | 92.43 | | | - | | | - | | SP(2) |
|  |  | 12(2) | 2.26 | 2.16 | | | | 1.99 | | 1.98 | | 84.09 | | | 161.00 | | 89.98 | | | | 97.94 | | | 79.89 | | | 82.50 | | SPY(1) |
|  |  | 13(2) | - | 1.99 | | | | 2.19 | | 1.95 | | - | | | 103.48 | | 110.74 | | | | 95.70 | | | - | | | - | | SPY(2) |
|  |  | 14(2) | 2.27 | 2.04 | | | | 2.03 | | 1.98 | | 89.75 | | | 163.54 | | 94.23 | | | | 95.89 | | | 78.85 | | | 82.07 | | SPY(2) |
|  |  | 16(2) | 2.23 | 2.02 | | | | 2.00 | | 2.04 | | 90.44 | | | 166.58 | | 93.48 | | | | 93.87 | | | 79.41 | | | 82.54 | | SPY(1) |
|  |  |  | MI-N | | MI-Ohy | | | | MI-O_Ca1_ | | MI-O_Ca2_ | N-MI-Ohy | | | Ohy-MI-Oca_1_ | | Ohy-MI-Oca_1_ | | | | Oca_1_-MI-Oca_2_ | | | N-MI-Oca_1_ | | | N-MI-Oca_2_ | |  |
|  |  | 19(1) | 2.02 | | 2.00 | | | | 1.97 | | 2.12 | 95.38 | | | 135.22 | | 112.50 | | | | 112.11 | | | 84.31 | | | 85.45 | | SPY(1) |
|  |  | 21(1) | 2.02 | | 2.00 | | | | 1.97 | | 2.12 | 95.38 | | | 135.22 | | 112.50 | | | | 112.11 | | | 84.31 | | | 85.45 | | SPY(1) |
|  |  | 23(1) | 2.02 | | 2.00 | | | | 1.97 | | 2.12 | 95.38 | | | 135.22 | | 112.50 | | | | 112.11 | | | 84.31 | | | 85.45 | | SPY(1) |
|  |  |  | MI-N | | MI-Ohy | | | | MI-Oca_1_ | | MI-Oca_2_ | N-MI-Ohy | | | Ohy-MI-Oca_1_ | | Ohy-MI-Oca_2_ | | | | Oca_1_-MI-Oca_2_ | | | N-MI-Oca_1_ | | | N-MI-Oca_2_ | |  |
|  |  | 30(1) | 2.04 | | 2.22 | | | | 1.93 | | 1.95 | 90.74 | | | 105.52 | | 103.63 | | | | 150.30 | | | 86.87 | | | 87.18 | | SPY(1) |
|  |  |  | MI-N | | | | MI-Ohy | | | MI-Oca_1_ | | N-MI-Ohy | | | | | | | Ohy-MI-Oca_1_ | | | | N-MI-Oca_1_ | | | | | |  |
|  |  | 36(1) | 2.02 | | | | 1.94 | | | 1.90 | | 95.31 | | | | | | | 172.66 | | | | 87.78 | | | | | | SP(1) |
|  |  | 38(1) | 2.03 | | | 1.91 | | | | 1.91 | | 96.27 | | | | | 171.07 | | | | | | | 87.66 | | | | | SP(1) |

**Table S3.** Bond lengths (Å) and angles (°) for the optimized complexes of Fe(MTB) with Oh, SP and Td geometries in HMSs 1 to 43 in Figure S5. The atom names are indicated in Figure S6. The number in parentheses indicates the number of water molecules involved in the coordination.

| Metal ion | Geometry | HMS | Bond lengths (Å) | | | | | | Angles (°) | | | | | | | | | | | | Pref. geometry |
| --- | --- | --- | --- | --- | --- | --- | --- | --- | --- | --- | --- | --- | --- | --- | --- | --- | --- | --- | --- | --- | --- |
|  |  |  | MI-N | | MI-Ohy | | | MI-Oca_1_ | N-MI-Ohy | | | | | | N-MI-Oca_1_ | | | Ohy-MI-Oca_1_ | | |  |
| Fe^II^ | Oh | 1(4) | - | | 2.02 | | | 2.04 | - | | | | | | - | | | 107.97 | | | Oh(4) |
|  |  | 2(4) | - | | 1.99 | | | 2.03 | - | | | | | | - | | | 116.49 | | | Oh(4) |
|  |  | 3(4) | - | | 2.00 | | | 2.03 | - | | | | | | - | | | 102.76 | | | Oh(4) |
|  |  | 4(4) | - | | 1.98 | | | 2.08 | - | | | | | | - | | | 118.75 | | | Oh(4) |
|  |  | 5(4) | - | | 2.00 | | | 2.03 | - | | | | | | - | | | 102.50 | | | Oh(4) |
|  |  | 6(4) | - | | 1.98 | | | 2.07 | - | | | | | | - | | | 118.74 | | | Oh(4) |
|  |  | 7(4) | - | | 1.99 | | | 2.03 | - | | | | | | - | | | 101.69 | | | Oh(4) |
|  |  | 8(4) | - | | 2.08 | | | 2.08 | - | | | | | | - | | | 118.51 | | | Oh(4) |
|  |  |  | MI-N | MI-Ohy | | MI-O_Ca1_ | | MI-O_Ca2_ | Oca_1_-MI-Oca_2_ | | | | | | | | | | | |  |
|  |  | 9(4) | - | - | | 2.00 | | 2.00 | 93.12 | | | | | | | | | | | | Oh(4) |
|  |  | 10(4) | - | - | | 2.00 | | 2.00 | 97.5 | | | | | | | | | | | | Oh(4) |
|  |  | 11(4) | - | - | | 2.00 | | 2.01 | 91.9 | | | | | | | | | | | | Oh(4) |
|  |  | 12(4) | - | - | | 2.02 | | 2.02 | 98.2 | | | | | | | | | | | | Oh(4) |
|  |  | 13(4) | - | - | | 2.00 | | 2.00 | 93.2 | | | | | | | | | | | | Oh(4) |
|  |  | 14(4) | - | - | | 2.00 | | 2.01 | 97.5 | | | | | | | | | | | | Oh(4) |
|  |  | 15(4) | - | - | | 2.01 | | 2.02 | 96.8 | | | | | | | | | | | | Oh(4) |
|  |  | 16(4) | - | - | | 2.02 | | 2.03 | 98.1 | | | | | | | | | | | | Oh(4) |
|  |  | 17(4) |  |  | | 1.98 | | 2.07 | 99.15 | | | | | | | | | | | | Oh(4) |
|  |  |  | MI-N | MI-Ohy | | MI-O_Ca1_ | | MI-O_Ca2_ | N-MI-Ohy | Ohy-MI-Oca_1_ | | | Ohy-MI-Oca_2_ | | | Oca_1_-MI-Oca_2_ | | | N-MI-Oca_1_ | N-MI-Oca_2_ |  |
|  |  | 18(3) | - | 2.10 | | 2.03 | | 2.00 | - | | | 114.30 | | 87.35 | | | 91.86 | | - | - | Oh(3) |
|  |  | 19(3) | - | 2.01 | | 2.08 | | 2.00 | - | | | 119.54 | | 88.10 | | | 104.38 | | - | - | Oh(3) |
|  |  | 20(3) | - | 2.04 | | 2.10 | | 1.98 | - | | | 117.06 | | 86.10 | | | 92.79 | | - | - | Oh(3) |
|  |  | 21(3) | - | 2.03 | | 2.12 | | 2.01 | - | | | 121.51 | | 99.79 | | | 91.8 | | - | - | Oh(3) |
|  |  | 22(3) | - | 2.09 | | 2.03 | | 2.01 | - | | 114.69 | | | 90.65 | | | 87.54 | | - | - | Oh(3) |
|  |  | 23(3) | - | 2.12 | | 2.07 | | 1.99 | - | | 124.62 | | | 93.09 | | | 96.11 | | - | - | Oh(3) |
|  |  | 24(3) | - | 2.09 | | 2.02 | | 2.02 | - | | 114.96 | | | 87.66 | | | 89.71 | | - | - | Oh(3) |
|  |  | 25(3) | - | 2.10 | | 2.07 | | 1.99 | - | | | 124.57 | | 89.71 | | | 93.28 | | - | - | Oh(3) |
|  |  |  | MI-N | | | | MI-Ohy | | N-MI-Ohy | | | | | | | | | | | |  |
|  |  | 26(4) | 2.11 | | | | 1.94 | | 85.33 | | | | | | | | | | | | Oh(4) |
|  |  | 27(4) | 2.10 | | | | 1.95 | | 86.07 | | | | | | | | | | | | Oh(4) |
|  |  | 28(4) | 2.10 | | | | 1.95 | | 86.03 | | | | | | | | | | | | Oh(4) |
|  |  | 29(4) | 2.09 | | | | 1.95 | | 86.34 | | | | | | | | | | | | Oh(4) |
|  |  |  | MI-N | MI-Ohy | | MI-Oca_1_ | | MI-Oca_2_ | N-MI-Ohy | | | Ohy-MI-Oca_1_ | | Ohy-MI-Oca_2_ | | | Oca_1_-MI-Oca_2_ | | N-MI-Oca_1_ | N-MI-Oca_2_ |  |
|  |  | 30(3) ^a^ | 2.12 | - | | 1.97 | | 1.97 | - | | | - | | - | | | 101.65 | | 85.51 | 81.44 | Oh(3) |
|  |  | 31(3) | 2.11 | - | | 1.96 | | 1.99 | - | | | - | | - | | | 100.22 | | 85.94 | 81.58 | Oh(3) |
|  |  | 32(3) | 2.10 | 2.01 | | 1.96 | | - | 82.25 | | | 169.51 | | - | | | - | | 87.32 | - | Oh(3) |
|  |  | 33(3) | 2.05 | 1.99 | | 1.95 | | - | 88.94 | | | 176.02 | | - | | | - | | 87.70 | - | Oh(3) |
|  |  | 34(3) | 2.07 | - | | 1.95 | | 1.96 | - | | | - | | - | | | 97.15 | | 87.15 | 83.08 | Oh(3) |
|  |  | 35(3) | 2.04 | 1.98 | | 1.99 | | - | 87.06 | | | 173.89 | | - | | | - | | 87.74 | 83.08 | Oh(3) |
|  |  |  | MI-N | MI-Ohy | | MI-Oca_1_ | | MI-Oca_2_ | N-MI-Ohy | | | Ohy-MI-Oca_1_ | | Oph-MI-Oca_2_ | | | Oca_1_-MI-Oca_2_ | | N-MI-Oca_1_ | N-MI-Oca_2_ |  |
|  |  | 31(1) | 1.98 | 1.99 | | 1.94 | | 1.96 | 86.36 | | | 173.51 | | 85.71 | | | 98.93 | | 89.41 | 84.71 | SPY(1) |
|  |  | 32(1) | 1.98 | 2.00 | | 1.94 | | 1.95 | 85.02 | | | 171.74 | | 86.45 | | | 99.10 | | 89.41 | 84.36 | SPY(1) |
|  |  | 33(1) | 1.99 | 1.99 | | 1.92 | | 1.91 | 92.68 | | | 163.01 | | 101.33 | | | 95.64 | | 88.42 | 88.73 | SPY(1) |
|  |  | 34(1) | 1.98 | 1.93 | | 1.97 | | 1.95 | 96.38 | | | 102.29 | | 105.92 | | | 151.57 | | 86.13 | 87.34 | SPY(1) |
|  |  | 35(1) | 1.99 | 1.98 | | 1.93 | | 1.98 | 96.94 | | | 99.84 | | 156.78 | | | 103.37 | | 87.48 | 83.69 | SPY(1) |
|  |  |  | MI-N | MI-Ohy | | MI-Oca_1_ | | MI-Oca_2_ | N-MI-Ohy | | | | | Ohy-MI-Oca_2_ | | | | | N-MI-Oca_2_ | |  |
|  |  | 36(3) | 2.00 | 1.93 | | 1.96 | | - | 89.26 | | | | | 95.40 | | | | | 84.57 | | Oh(3) |
|  |  | 37(3) | 2.00 | 1.93 | | 1.97 | | - | 89.34 | | | | | 95.41 | | | | | 84.39 | | Oh(3) |
|  |  | 38(3) | 2.00 | 1.93 | | 1.97 | | - | 89.41 | | | | | 95.63 | | | | | 84.32 | | Oh(3) |
|  |  | 39(3) | 2.00 | 1.93 | | 1.98 | | - | 89.36 | | | | | 95.27 | | | | | 84.27 | | Oh(3) |

**Cont.**

|  |  | HMS | Bond lengths (Å) | | | | | | | | | Angles (°) | | | | | | | | | Pref. geometry |
| --- | --- | --- | --- | --- | --- | --- | --- | --- | --- | --- | --- | --- | --- | --- | --- | --- | --- | --- | --- | --- | --- |
|  |  |  | MI-N | MI-Ohy | | | MI-Oca_1_ | | | MI-Oca_2_ | | N-MI-Ohy | Ohy-MI-Oca_1_ | | Ohy-MI-Oca_2_ | | Oca_1_-MI-Oca_2_ | | N-MI-Oca_1_ | N-MI-Oca_2_ |  |
|  |  | 40(2) ^a^ | 1.99 | 2.00 | | | 1.95 | | | 1.99 | | 94.74 | 95.26 | | 170.42 | | 94.16 | | 87.41 | 83.99 | Oh(2) |
|  |  | 41(2) | 1.99 | 1.99 | | | 1.96 | | | 2.00 | | 94.78 | 95.22 | | 172.44 | | 92.09 | | 87.62 | 83.75 | Oh(2) |
|  |  | 42(2) | 1.99 | 1.99 | | | 1.94 | | | 1.99 | | 95.77 | 96.52 | | 97.95 | | 97.95 | | 86.98 | 84.25 | Oh(2) |
|  |  | 43(2) | 1.99 | 2.00 | | | 1.95 | | | 1.99 | | 94.78 | 95.29 | | 170.79 | | 93.77 | | 87.50 | 83.98 | Oh(2) |
| Fe^II^ | SP |  | MI-N | | | MI-Ohy | | | MI-O_Ca1_ | | | N-MI-Ohy | | | N-MI-Oca_1_ | | | | Ohy-MI-Oca_1_ | |  |
|  |  | 7(2) | - | | | 2.00 | | | 1.97 | | | - | | | - | | | | 96.12 | | SP(2) |
|  |  | 8(2) | 1.96 | | | 1.97 | | | 1.98 | | | 93.09 | | | 84.69 | | | | 177.64 | | SPY(2) |
|  |  |  | MI-N | | | MI-O_Ca1_ | | | MI-O_Ca2_ | | | Oca_1_-MI-Oca_2_ | | | N-MI-Oca_1_ | | | | N-MI-Oca_2_ | |  |
|  |  | 9(2) | - | | | 1.96 | | | 1.96 | | | 99.70 | | | - | | | | - | | SP(2) |
|  |  | 12(2) | 2.03 | | | 1.97 | | | 1.95 | | |  | | | 82.5 | | | | 85.9 | | SPY(2) |
|  |  | 14(2) | 2.04 | | | 2.00 | | | 1.94 | | | 99.37 | | | 81.95 | | | | 85.23 | | SPY(2) |
|  |  | 16(2) | 2.04 | | | 2.00 | | | 1.95 | | |  | | | 82.0 | | | | 85.60 | | SPY(2) |
|  |  | 17(2) | 2.04 | | | 2.00 | | | 1.96 | | | 95.55 | | | 82.09 | | | | 85.90 | | SPY(2) |
|  |  |  | MI-N | MI-Ohy | | | MI-Oca_1_ | | | MI-O_Ca2_ | | N-MI-Ohy | Ohy-MI-Oca_1_ | | Ohy-MI-Oca_2_ | | Oca_1_-MI-Oca_2_ | | N-MI-Oca_1_ | N-MI-Oca_2_ |  |
|  |  | 19(1) | 1.97 | 1.97 | | | 1.99 | | | 1.94 | | 96.92 | 169.96 | | 92.35 | | 97.67 | | 83.17 | 86.66 | SPY(1) |
|  |  | 21(1) | 1.98 | 1.97 | | | 2.03 | | | 1.96 | | 96.23 | 170.46 | | 92.49 | | 96.9 | | 82.63 | 86.38 | SPY(1) |
|  |  | 23(1) | 1.98 | 1.97 | | | 2.02 | | | 1.96 | | 96.29 | 170.43 | | 92.31 | | 97.13 | | 82.81 | 86.45 | SPY(1) |
|  |  | 25(1) | 1.95 | 1.80 | | | 1.86 | | | 1.81 | | 96.42 | 161.42 | | 100.98 | | 97.58 | | 83.79 | 86.73 | SPY(1) |
|  |  |  | MI-N | MI-Ohy | | | MI-Oca_1_ | | | MI-Oca_2_ | | N-MI-Ohy | Ohy-MI-Oca_1_ | | Oph-MI-Oca_2_ | | Oca_1_-MI-Oca_2_ | | N-MI-Oca_1_ | N-MI-Oca_2_ |  |
|  |  | 26(2) | 1.98 | 1.96 | | | 1.96 | | | 2.00 | | 95.72 | 99.33 | | 92.28 | | 167.38 | | 86.73 | 87.21 | Oh(2) |
|  |  | 27(2) | 1.98 | 1.96 | | | 1.98 | | | 2.03 | | 95.36 | 95.36 | | 98.12 | | 161.93 | | 86.08 | 86.77 | Oh(2) |
|  |  | 28(2) | 1.98 | 1.96 | | | 1.98 | | | 2.02 | | 95.63 | 95.63 | | 95.39 | | 164.93 | | 86.39 | 86.91 | Oh(2) |
|  |  |  | MI-N | | MI-Ohy | | | MI-Oca_1_ | | | MI-Oca_2_ | N-MI-Ohy | | Ohy-MI-Oca_1_ | | Ohy-MI-Oca_2_ | | Oca_1_-MI-Oca_2_ | N-MI-Oca_1_ | N-MI-Oca_2_ |  |
|  |  | 36(1) | 1.97 | | 1.93 | | | 1.96 | | | 1.96 | 90.26 | | 172.76 | | 91.85 | | 95.27 | 89.14 | 84.53 | SPY(1) |
|  |  | 37(1) | 1.97 | | 1.94 | | | 1.99 | | | 1.97 | 89.64 | | 172.62 | | 91.75 | | 95.15 | 88.49 | 84.02 | SPY(1) |
|  |  | 38(1) | 1.97 | | 1.94 | | | 1.99 | | | 1.97 | 89.68 | | 172.00 | | 92.23 | | 95.31 | 88.48 | 84.03 | SPY(1) |
|  |  | 39(1) | 1.97 | | 1.94 | | | 2.01 | | | 1.99 | 89.46 | | 173.16 | | 92.10 | | 93.95 | 88.04 | 83.80 | SPY(1) |
| Fe^II^ | Td |  | MI-N | | MI-Ohy | | | MI-Oca_1_ | | | MI-Oca_2_ | N-MI-Ohy | | N-MI-Oca_1_ | | N-MI-Oca_2_ | | Ohy-MI-Oca_1_ | Ohy-MI-Oca_2_ | Oca_1_-MI-Oca_2_ |  |
|  |  | 2(2) | 2.00 | | 2.00 | | | 1.99 | | | 1.96 | 95.91 | | 82.49 | | 86.24 | | 169.75 | 92.55 | - | Oh(2) |
|  |  | 4(2) | 2.01 | | 1.96 | | | 2.01 | | | - | 95.96 | | 82.98 | | - | | 173.73 | - | - | SPY(2) |
|  |  | 6(2) | 2.01 | | 1.96 | | | 2.01 | | | - | 95.77 | | 83.41 | | - | | 174.52 |  |  | SPY(2) |
|  |  | 8(2) | 2.02 | | 1.95 | | | 2.03 | | | - | 96.14 | | 82.46 | | - | | 173.82 | - | - | SPY(2) |
|  |  |  | MI-N | | MI-Ohy | | | MI-Oca_1_ | | | MI-Oca_2_ | N-MI-Ohy | | Ohy-MI-Oca_1_ | | Ohy-MI-Oca_2_ | | Oca_1_-MI-Oca_2_ | N-MI-Oca_1_ | N-MI-Oca_2_ |  |
|  |  | 10(2) | 2.01 | | 2.07 | | | 1.97 | | | 1.95 | 90.99 | | 169.32 | | 90.10 | | 98.63 | 83.53 | 86.03 | Oh(2) |
|  |  | 12(2) | 2.01 | | 2.06 | | | 1.97 | | | 1.96 | 90.85 | | 169.68 | | 90.81 | | 97.36 | 83.48 | 85.95 | Oh(2) |
|  |  | 14(2) | 2.00 | | 1.99 | | | 1.97 | | | 1.96 | 95.54 | | 173.44 | | 93.03 | | 93.37 | 83.31 | 87.16 | Oh(2) |
|  |  | 16(2) | 2.00 | | 2.02 | | | 1.99 | | | 1.98 | 94.20 | | 172.33 | | 94.09 | | 92.71 | 82.66 | 86.46 | Oh(2) |
|  |  |  | MI-N | | MI-Ohy | | | MI-O_Ca1_ | | | MI-O_Ca2_ | N-MI-Ohy | | Ohy-MI-Oca_1_ | | Ohy-MI-Oca_1_ | | Oca_1_-MI-Oca_2_ | N-MI-Oca_1_ | N-MI-Oca_2_ |  |
|  |  | 19(1) | 1.98 | | 1.97 | | | 1.94 | | | 1.92 | 95.87 | | 157.16 | | 101.62 | | 101.22 | 88.04 | 84.78 | SPY(1) |
|  |  | 21(1) | 1.99 | | 1.92 | | | 1.93 | | | 1.97 | 94.13 | | 160.41 | | 107.93 | | 91.6 | 86.51 | 88.58 | SPY(1) |
|  |  | 23(1) | 1.98 | | 1.97 | | | 1.92 | | | 1.94 | 95.87 | | 160.41 | | 107.93 | | 101.22 | 84.78 | 88.04 | SPY(1) |
|  |  | 25(1) | 1.99 | | 1.90 | | | 1.94 | | | 1.98 | 94.89 | | 155.25 | | 114.98 | | 89.72 | 85.92 | 88.25 | SPY(1) |
|  |  |  | MI-N | | MI-Ohy | | | MI-Oca_1_ | | | MI-Oca_2_ | N-MI-Ohy | | Ohy-MI-Oca_1_ | | Oph-MI-Oca_2_ | | Oca_1_-MI-Oca_2_ | N-MI-Oca_1_ | N-MI-Oca_2_ |  |
|  |  | 26(2) ^a^ | 2.00 | | 1.94 | | | 1.96 | | | 1.97 | 89.87 | | 175.45 | | 92.02 | | 92.30 | 89.10 | 84.86 | Oh(2) |
|  |  | 27(2) | 1.99 | | 1.95 | | | 1.99 | | | 1.99 | 89.09 | | 176.08 | | 91.81 | | 91.26 | 88.78 | 84.40 | Oh(2) |
|  |  | 28(2) | 2.00 | | 1.94 | | | 1.98 | | | - | 88.88 | | 173.78 | | - | | - | 88.12 | - | SPY(2) |

**Discussion on the Geometrical Parameters in Tables S1 to S3.** The Zn^II^–Oca_1_ (1.93 to 2.26 Å), Zn^II^–Oca_2_ (1.96 to 2.19 Å), Zn^II^–Ohy (1.91 to 2.24 Å), Zn^II^–N (2.12 to 2.28 Å), Cu^II^–Oca_1_ (1.88 to 2.21 Å), Cu^II^–Oca_2_ (1.89 to 2.25 Å), Cu^II^–Ohy (1.93 to 2.30 Å), Cu^II^–N (2.02 to 2.54 Å), Fe^II^–Oca_1_ (1.86 to 2.12 Å), Fe^II^–Oca_2_ (1.81 to 2.07 Å), Fe^II^–Ohy (1.80 to 2.12 Å) and Fe^II^–N (1.94 to 2.12 Å) computed bond lengths are all comparable with the reported normal ranges for other complexes of the studied MIs.^24-30^ Outstanding results of the optimization processes can be summarized as following: *i*) Optimized HMSs always show Oh geometries for Fe^II^ complexes. *ii*) In all optimized HMSs a protonated tertiary amine nitrogen atom does not participate in the coordination spheres. However, a phenolic group can participate in coordination in –OH and –O^-^ forms. *iii*) Tendency of the MIs toward the nitrogen atom decreases according to Fe^II^ > Zn^II^ > Cu^II^ (The bond lengths of N-Cu^II^ have the longest values) i*v*) Apical distances in complexes with the SPY geometry for Cu^II^ are longer than the equatorial ones. Lengthening of these bonds is not a Jahn-Teller distortion. On the other hand, it is a consequence of the double electron occupancy of the antibonding a1 (dz^2^) orbital, and the single occupancy of b1 (d_x_^2^-_y_^2^) orbital. These lead to an increased antibonding electron density along the apical axis.^31^

**Table S4.** Wavelength (*λ*), excitation energy (*E*), difference with the experimental values ($\Delta$*λ*), and oscillator strengths (*f*) of lowest computed energy transitions for the obtained Zn(MTB) complexes.

| Geometry | No. HMS | Transitions from 500 to 700 nm | | | Geometry | No. HMS | Transitions from 500 to 700 nm | | |
| --- | --- | --- | --- | --- | --- | --- | --- | --- | --- |
|  |  | λ/nm (*E*/eV) | $\Delta$λ/nm(*E*, eV) | *f* |  |  | λ/nm (*E*/eV) | $\Delta$λ/nm(*E*, eV) | *f* |
| Oh | Experiment | 600(2.07) |  |  | Td | Experiment | 600(2.07) |  |  |
|  | 1-Oh(4) | 537(2.31) | 63(0.24) | 0.61 |  | 1-Td(2) | 562(2.20) | 38(0.14) | 0.54 |
|  | 2-Oh(4) | 438(2.83) | 162(0.76) | 0.59 |  | 2-Td(2) | 511(2.43) | 89(0.36) | 0.65 |
|  | 3-Oh(4) | 503(2.47) | 97(40) | 0.34 |  | 3-Td(2) | 549(2.26) | 51(0.19) | 0.22 |
|  | 4-Oh(4) | 565(2.19) | 35(0.13) | 0.58 |  | 4-Td(2) | 565(2.20) | 35(0.13) | 0.53 |
|  | 5-Oh(4) | 498(2.49) | 102(0.43) | 0.35 |  | 5-Td(2) | 546(2.27) | 54(0.21) | 0.29 |
|  | 6-Oh(4) | 558(2.22) | 42(0.16) | 0.62 |  | 6-Td(2) | 574(2.16) | 26(0.10) | 0.52 |
|  | 7-Oh(4) | 483(2.56) | 117(0.50) | 0.07 |  | 7-Td(2) | 514(2.41) | 86(0.34) | 0.17 |
|  | **8-**Oh(4) | **584(2.12)** | **16(0.23)** | **0.26** |  | 8-Td(2) | 553(2.24) | 47(0.18) | 0.32 |
|  | 9-Oh(4) | 533(2.32) | 67(0.26) | 0.56 |  | 9-Td(2) | 477(2.60) | 123(0.53) | 0.45 |
|  | 10-Oh(4) | 497(2.49) | 103(0.43) | 0.63 |  | 10-Td(2) | 473(2.62) | 127(0.55) | 0.52 |
|  | 13-Oh(4) | 533(2.32) | 67(0.26) | 0.56 |  | 13-Td(2) | 558(2.22) | 42(0.16) | 0.70 |
|  | 14-Oh(4) | 497(2.49) | 103(0.43) | 0.63 |  | 19-Td(1) | 513(2.42) | 87(0.35) | 0.61 |
|  | 20-Oh(3) | 548(2.26) | 52(0.20) | 0.14 |  | **20-Td(1)** | **606(2.05)** | **6(0.02)** | **0.09** |
|  | 21-Oh(3) | 557(2.23) | 43(0.16) | 0.63 |  | 22-Td(1) | 535(2.32) | 65(0.25) | 0.65 |
|  | 22-Oh(3) | 549(2.26) | 51(0.19) | 0.33 |  | 23-Td(1) | 542(2.29) | 58(0.22) | 0.61 |
|  | 23-Oh(3) | 557(2.22) | 43(0.16) | 0.53 |  | 25-Td(1) | 544(2.28) | 56(0.21) | 0.33 |
|  | 25-Oh(3) | 553(2.24) | 47(0.18) | 0.16 |  |  |  |  |  |
|  | 27-Oh(4) | 554(2.24) | 46(0.17) | 0.67 |  |  |  |  |  |
|  | 28-Oh(4) | 556(2.23) | 44(0.16) | 0.64 |  |  |  |  |  |
|  | 29-Oh(4) | 555(2.23) | 45(0.17) | 0.38 |  |  |  |  |  |
|  | 34-Oh(3) | 518(2.39) | 82(0.33) | 0.62 |  |  |  |  |  |
|  | **35-**Oh(3) | **587(2.11)** | **13(0.05)** | **0.53** |  |  |  |  |  |
|  | 37-Oh(3) | 569(2.18) | 31(0.11) | 0.64 |  |  |  |  |  |
|  | 38-Oh(3) | 560(2.21) | 40(0.15) | 0.62 |  |  |  |  |  |
|  | 39-Oh(3) | 571(2.17) | 29(0.11) | 0.41 |  |  |  |  |  |
|  | 42-Oh(2) | 515(2.41) | 85(0.34) | 0.62 |  |  |  |  |  |
| SP | 2-SP(2) | 506(2.45) | 94(0.38) | 0.54 |  |  |  |  |  |
|  | 4-SP(2) | 565(2.19) | 35(0.13) | 0.50 |  |  |  |  |  |
|  | 5-SP(2) | 536(2.31) | 64(0.25) | 0.50 |  |  |  |  |  |
|  | 6-SP(2) | 573(2.16) | 27(0.1) | 0.51 |  |  |  |  |  |
|  | 8-SP(2) | 552(2.24) | 48(0.18) | 0.22 |  |  |  |  |  |
|  | 9-SP(2) | 496(2.50) | 104(0.43) | 0.58 |  |  |  |  |  |
|  | 11-SP(2) | 449(2.76) | 151(0.70) | 0.40 |  |  |  |  |  |
|  | 17-SP(2) | 583(2.13) | 85(0.36) | 0.32 |  |  |  |  |  |
|  | 19-SP(1) | 513(2.42) | 87(0.35) | 0.65 |  |  |  |  |  |
|  | 21-SP(1) | 570(2.17) | 30(0.11) | 0.52 |  |  |  |  |  |
|  | **23-SP(1)** | **578(2.14)** | **22(0.08)** | **0.51** |  |  |  |  |  |
|  | 30-SP(1) | 444(2.79) | 156(0.73) | 0.44 |  |  |  |  |  |
|  | **31-SP(1)** | **595(2.08)** | **5(0.02)** | **0.22** |  |  |  |  |  |
|  | 32-SP(1) | 448(2.77) | 152(0.70) | 0.43 |  |  |  |  |  |
|  | 33-SP(1) | 1439(0.86) | 839(1.20) | 0.13 |  |  |  |  |  |
|  | 35-SP(1) | 538(2.30) | 62(0.24) | 0.61 |  |  |  |  |  |

**Table S5.** Wavelength (*λ*), excitation energy (*E*), difference with the experimental values ($\Delta$*λ*), and oscillator strengths (*f*) of lowest computed energy transitions for the obtained Cu(MTB) complexes.

| Geometry | No. HMS | Transitions from 500 to 700 nm | | | Geometry | No. HMS | Transitions from 500 to 700 nm | | |
| --- | --- | --- | --- | --- | --- | --- | --- | --- | --- |
|  |  | λ/nm (*E*/eV) | $\Delta$*λ/nm(E, eV)* | *f* |  |  | λ/nm (*E*/eV) | $\Delta$*λ/nm(E, eV)* | *f* |
| Oh | Experiment | 600(2.07) |  |  | Td | Experiment | 600(2.07) |  |  |
|  | 1-Oh(4) | 695(1.78) | 95(0.29) | 0.14 |  | 2-Td(2) | 513(2.42) | 88(0.35) | 0.62 |
|  | 3-Oh(4) | 679(1.82) | 79(0.24) | 0.05 |  | 9-Td(2) | 488(2.54) | 112(0.47) | 0.55 |
|  | 4-Oh(4) | 692(1.79) | 92(0.22) | 0.05 |  | 12-Td(2) | 492(2.52) | 108(0.45) | 0.13 |
|  | 5-Oh(4) | 658(1.88) | 58(0.19) | 0.10 |  | 13-Td(2) | 571(2.17) | 29(0.10) | 0.08 |
|  | 6-Oh(4) | 574(2.16) | 26(0.09) | 0.29 |  | 14-Td(2) | 755(1.64) | 155(0.43) | 0.05 |
|  | 7-Oh(4) | 636(1.95) | 36(0.12) | 0.14 |  | 16-Td(2) | 824(1.50) | 224(0.56) | 0.05 |
|  | **8-Oh(4)** | **588(2.11)** | **12(0.04)** | **0.08** |  | 19-Td(1) | 516(2.40) | 84(0.33) | 0.63 |
|  | 9-Oh(4) | 537(2.13) | 63(0.24) | 0.62 |  | 21-Td(1) | 575(2.16) | 25(0.09) | 0.19 |
|  | 10-Oh(4) | 551(2.25) | 49(0.18) | 0.62 |  | 23-Td(1) | 516(2.40) | 84(0.33) | 0.63 |
|  | 12-Oh(4) | 512(2.42) | 88(0.35) | 0.57 |  | 30-Td(1) | 467(2.66) | 133(0.59) | 0.45 |
|  | 16-Oh(4) | 515(2.41) | 85(0.34) | 0.48 |  | 36-Td(1) | 524(2.36) | 76(0.29) | 0.17 |
|  | 18-Oh(3) | 551(2.25) | 49(0.18) | 0.62 |  | 38-Td(1) | 527(2.35) | 73(0.28) | 0.40 |
|  | 19-Oh(3) | 519(2.39) | 81(0.32) | 0.60 |  |  |  |  |  |
|  | 20-Oh(3) | 963(1.29) | 363(0.78) | 0.06 |  |  |  |  |  |
|  | **21-Oh(3)** | **593(2.09)** | **7(0.02)** | **0.17** |  |  |  |  |  |
|  | 22-Oh(3) | 523(2.37) | 77(0.30) | 0.38 |  |  |  |  |  |
|  | 23-Oh(3) | 1075(1.15) | 475(0.91) | 0.09 |  |  |  |  |  |
|  | 25-Oh(3) | 706(1.76) | 106(0.31) | 0.05 |  |  |  |  |  |
|  | **27-Oh(4)** | **614(2.02)** | **14(0.05)** | **0.05** |  |  |  |  |  |
|  | 29-Oh(4) | 644(1.99) | 44(0.14) | 0.05 |  |  |  |  |  |
|  | 36-Oh(3) | 534(2.32) | 66(0.35) | 0.11 |  |  |  |  |  |
|  | **37-Oh(3)** | **592(2.09)** | **8(0.03)** | **0.06** |  |  |  |  |  |
|  | **38-Oh(3)** | **593(2.09)** | **7(0.02)** | **0.14** |  |  |  |  |  |
|  | **40-Oh(2)** | **608(2.04)** | **8(0.03)** | **0.14** |  |  |  |  |  |
| SP | 2-SP(2) | 656(1.89) | 56(0.18) | 0.07 |  |  |  |  |  |
|  | 4-SP(2) | 782(1.59) | 182(0.48) | 0.06 |  |  |  |  |  |
|  | 5-SP(2) | 828(1.50) | 228(0.57) | 0.11 |  |  |  |  |  |
|  | 6-SP(2) | 781(1.59) | 181(0.48) | 0.06 |  |  |  |  |  |
|  | 9-SP(2) | 496(2.50) | 104(0.43) | 0.56 |  |  |  |  |  |
|  | 10-SP(2) | 474(2.62) | 126(0.55) | 0.36 |  |  |  |  |  |
|  | 12-SP(2) | 483(2.57) | 117(0.50) | 0.14 |  |  |  |  |  |
|  | 13-SP(2) | 568(2.18) | 32(0.11) | 0.34 |  |  |  |  |  |
|  | 14-SP(2) | 508(2.44) | 92(0.37) | 0.59 |  |  |  |  |  |
|  | 15-SP(2) | 556(2.23) | 44(0.16) | 0.25 |  |  |  |  |  |
|  | **16-SP(2)** | **578(2.15)** | **22(0.08)** | **0.57** |  |  |  |  |  |
|  | 17-SP(2) | 649(1.91) | 49(0.16) | 0.07 |  |  |  |  |  |
|  | 21-SP(1) | 691(1.79) | 91(0.27) | 0.14 |  |  |  |  |  |
|  | 23-SP(1) | 848(1.46) | 248(0.61) | 0.05 |  |  |  |  |  |
|  | **28-SP(2)** | 586(2.12) | 14(0.05) | 0.56 |  |  |  |  |  |
|  | 30-SP(1) | 475(2.61) | 125(0.54) | 0.05 |  |  |  |  |  |
|  | 32-SP(1) | 442(2.80) | 158(0.73) | 0.09 |  |  |  |  |  |
|  | 34-SP(1) | 493(2.52) | 107()0.45 | 0.58 |  |  |  |  |  |
|  | 35-SP(1) | 639(1.94) | 39(0.13) | 0.08 |  |  |  |  |  |
|  | 36-SP(1) | 557(2.23) | 43(0.16) | 0.10 |  |  |  |  |  |
|  | **37-SP(1)** | **585(2.12)** | **15(0.05)** | **0.23** |  |  |  |  |  |
|  | **38-SP(1)** | **588(2.11)** | **12(0.04)** | **0.31** |  |  |  |  |  |
|  | 39-SP(1) | 586(2.12) | 14(0.05) | 0.12 |  |  |  |  |  |

**Table S6.** Wavelength (*λ*), excitation energy (*E*), difference with the experimental values ($\Delta$*λ*) and oscillator strengths (*f*) of lowest computed energy transitions for the obtained Fe(MTB) complexes.

| Geometry | No. HMS | Transitions from 400 to 550 nm | | | Geometry | No. HMS | Transitions from 400 to 550 nm | | |
| --- | --- | --- | --- | --- | --- | --- | --- | --- | --- |
|  |  | λ/nm (*E*/eV) | $\Delta$*λ/nm(E, eV)* | *f* |  |  | λ/nm (*E*/eV) | $\Delta$*λ/nm(E, eV)* | *f* |
| Oh | Experiment | 498(2.49) |  |  | Td | 2-Td(2) | 900(1.38) | 402(1.11) | 0.05 |
|  | 1-Oh(4) | 580(2.14) | 82(0.35) | 0.23 |  | 4-Td(2) | 673(1.84) | 175(0.65) | 0.24 |
|  | 2-Oh(4) | 584(2.12) | 86(0.37) | 0.32 |  | 6-Td(2) | 639(1.94) | 141(0.55) | 0.25 |
|  | 3-Oh(4) | 552(2.14) | 54(0.25) | 0.20 |  | 8-Td(2) | 591(2.10) | 93(0.39) | 0.13 |
|  | 4-Oh(4) | 676(1.83) | 178(0.66) | 0.42 |  | 10-Td(2) | 467(2.65) | 31(17) | 0.55 |
|  | 5-Oh(4) | 550(2.25) | 52(0.23) | 0.20 |  | **12-Td(2)** | **493(2.52)** | **5(0.03)** | **0.11** |
|  | 6-Oh(4) | 654(1.90) | 156(0.59) | 0.40 |  | 14-Td(2) | 783(1.58) | 285(0.91) | 0.10 |
|  | 7-Oh(4) | 489(2.53) | 9(0.04) | 0.10 |  | 16-Td(2) | 677(1.83) | 179(0.66) | 0.12 |
|  | 8-Oh(4) | 678(1.83) | 180(0.66) | 0.20 |  | 19-Td(1) | 739(1.68) | 241(0.81) | 0.10 |
|  | 9-Oh(4) | 535(2.32) | 37(0.17) | 0.52 |  | 21-Td(1) | 667(1.86) | 169(0.63) | 0.16 |
|  | 10-Oh(4) | 467(2.65) | 31(0.17) | 0.55 |  | 23-Td(1) | 739(1.68) | 241(0.81) | 0.10 |
|  | 11-Oh(4) | 539(2.30) | 41(0.19) | 0.25 |  | 25-Td(1) | 651(1.90) | 153(0.59) | 0.07 |
|  | 12-Oh(4) | 526(2.36) | 28(0.13) | 0.32 |  | 26-Td(2) | 774(1.60) | 276(0.89) | 0.05 |
|  | 13-Oh(4) | 535(2.32) | 37(0.17) | 0.52 |  | 27-Td(2) | 739(1.68) | 241(0.81) | 0.08 |
|  | **14-Oh(4)** | **501(2.47)** | **3(0.02)** | **0.27** |  | 28-Td(2) | 641(1.94) | 143(0.55) | 0.42 |
|  | **15-Oh(4)** | **517(2.40)** | **19(0.09)** | **0.51** | **SP** | 7-SP(2) | 554(2.24) | 56(0.25) | 0.22 |
|  | 16-Oh(4) | 529(2.35) | 31(014) | 0.31 |  | 8-SP(2) | 559(2.22) | 61(0.27) | 0.28 |
|  | 17-Oh(4) | 539(2.09) | 41(0.19) | 0.08 |  | **9-SP(2)** | **497(2.49)** | **1(0.00)** | **0.53** |
|  | 18-Oh(3) | 556(2.23) | 58(0.26) | 0.39 |  | **12-SP(2)** | **484(2.56)** | **14(0.07)** | **0.13** |
|  | 19-Oh(3) | 739(1.68) | 241(0.18) | 0.10 |  | **14-SP(2)** | **508(2.44)** | **10(0.05)** | **0.61** |
|  | 20-Oh(3) | 564(2.20) | 66(0.29) | 0.36 |  | 16-SP(2) | 582(2.13) | 84(0.36) | 0.52 |
|  | 21-Oh(3) | 722(1.72) | 224(0.77) | 0.05 |  | 17-SP(2) | 589(2.10) | 91(0.38) | 0.32 |
|  | 22-Oh(3) | 556(2.23) | 58(0.26) | 0.39 |  | 19-SP(1) | 666(1.86) | 168(0.63) | 0.07 |
|  | 23-Oh(3) | 611(2.03) | 113(0.46) | 0.35 |  | 21-SP(1) | 657(1.89) | 159(0.60) | 0.14 |
|  | **24-Oh(3)** | **486(2.55)** | **12(0.06)** | **0.11** |  | 23-SP(1) | 664(1.87) | 166(0.62) | 0.14 |
|  | 25-Oh(3) | 598(2.07) | 100(0.42) | 0.36 |  | 25-SP(1) | 1048(1.18) | 550(1.31) | 0.34 |
|  | 26-Oh(4) | 625(1.98) | 127(0.51) | 0.32 |  | 26-SP(2) | 827(1.50) | 329(0.99) | 0.07 |
|  | 27-Oh(4) | 648(1.91) | 150(0.58) | 0.39 |  | 27-SP(2) | 650(1.91) | 152(0.58) | 0.27 |
|  | 28-Oh(4) | 649(1.91) | 151(0.58) | 0.38 |  | 28-SP(2) | 647(1.92) | 149(0.57) | 0.22 |
|  | 29-Oh(4) | 642(1.93) | 144(0.56) | 0.29 |  | **31-SP(1)** | **507(2.45)** | **9(0.04)** | **0.50** |
|  | 30-Oh(3) | 462(2.68) | 36(0.19) | 0.35 |  | **32-SP(1)** | **491(2.52)** | **7(0.04)** | **0.07** |
|  | 31-Oh(3) | 431(2.88) | 67(0.39) | 0.35 |  | 33-SP(1) | 412(3.01) | 86(0.52) | 0.07 |
|  | 32-Oh(3) | 526(2.36) | 28(0.13) | 0.21 |  | 34-SP(1) | 702(1.77) | 204(0.72) | 0.31 |
|  | 33-Oh(3) | 1127(1.10) | 629(1.39) | 0.17 |  | 35-SP(1) | 656(1.89) | 158(0.60) | 0.28 |
|  | **34-Oh(3)** | **518(2.40)** | **19(0.09)** | **0.61** |  | 36-SP(1) | 672(1.84) | 174(0.65) | 0.23 |
|  | 35-Oh(3) | 653(1.90) | 155(0.59) | 0.36 |  | 37-SP(1) | 739(1.68) | 241(0.81) | 0.07 |
|  | 36-Oh(3) | 631(1.97) | 133(0.52) | 0.05 |  | 38-SP(1) | 746(1.66) | 248(0.83) | 0.07 |
|  | 37-Oh(3) | 625(.198) | 127(0.51) | 0.49 |  | 39-SP(1) | 651(1.90) | 153(0.58) | 0.21 |
|  | 38-Oh(3) | 675(1.84) | 177(0.65) | 0.05 |  |  |  |  |  |
|  | 39-Oh(3) | 669(1.85) | 171(0.64) | 0.22 |  |  |  |  |  |
|  | 40-Oh(2) | 666(1.86) | 168(0.63) | 0.17 |  |  |  |  |  |
|  | 41-Oh(2) | 658(1.88) | 160(0.61) | 0.16 |  |  |  |  |  |
|  | 42-Oh(2) | 683(1.82) | 185(0.67) | 0.09 |  |  |  |  |  |
|  | 43-Oh(2) | 791(1.57) | 293(0.92) | 0.05 |  |  |  |  |  |


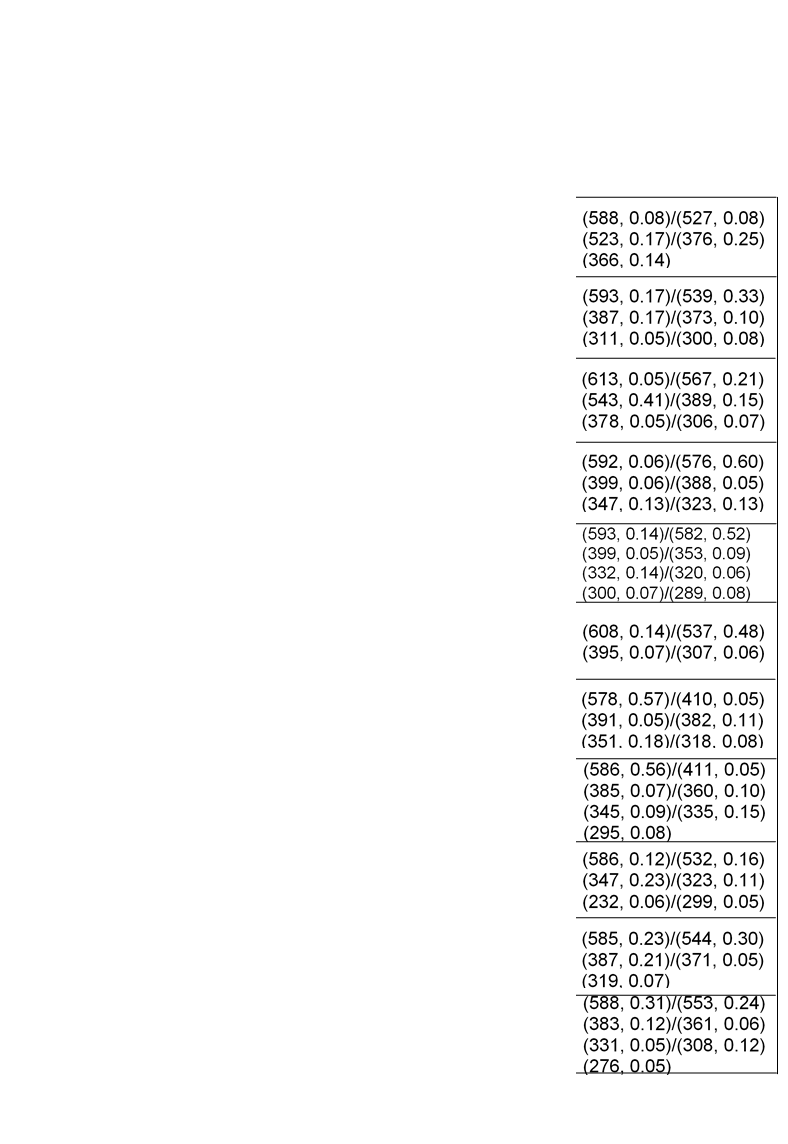


No. HMS

8-Oh (4)

21-Oh (1)

27-Oh (4)

37-Oh (3)

38-Oh (3)

40-Oh (2)

16-SP (2)

38-SP (2)

37-SP (1)

38-SP (1)

39-SP (1)

Zn(MTB)

No. HMS

8-Oh (4)

35-Oh (3)

31-SP (1)

23-SP (1)

20-Td (1) (1)


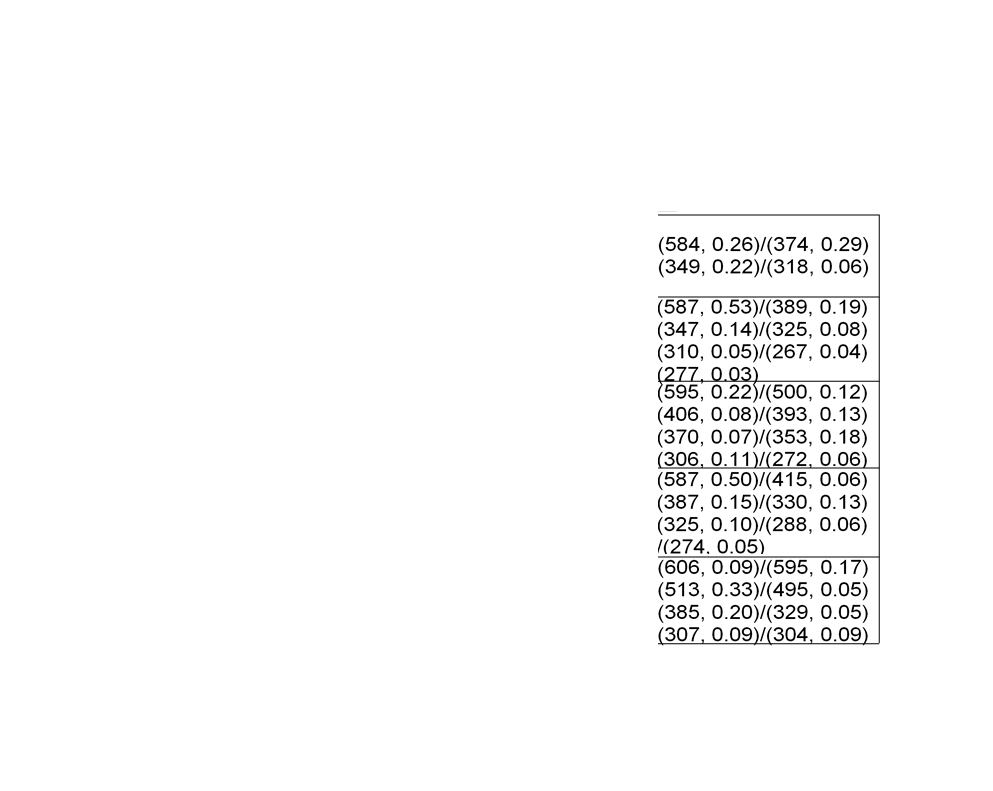

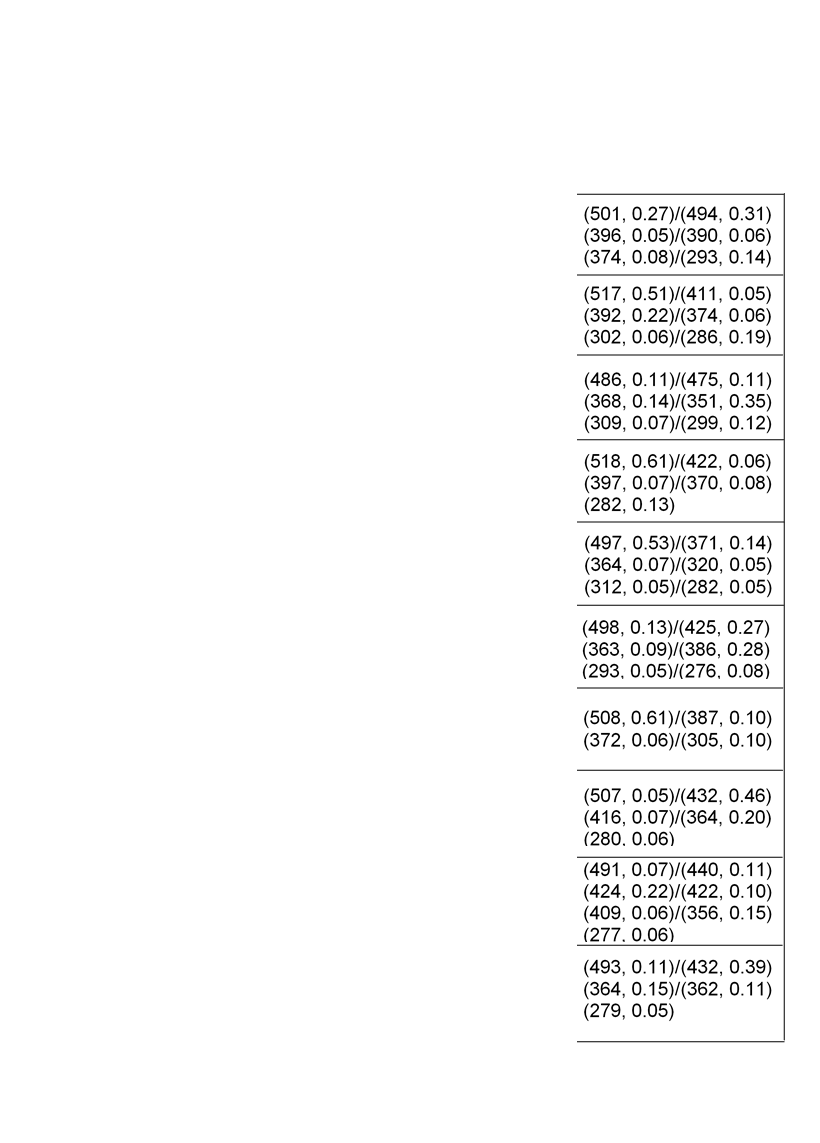


Fe(MTB)

No. HMS

14-Oh (4)

15-Oh (4)

24-Oh (4)

34-Oh (3)

9-SP (2)

12-SP (2)

14-SP (2)

31-SP (1)

32-SP (1)

12-Td (2)

Fe_2_(MTB)

No. HMS

13-Oh (4)

16-Oh (3)

17-Oh (3)

21-Oh (3)


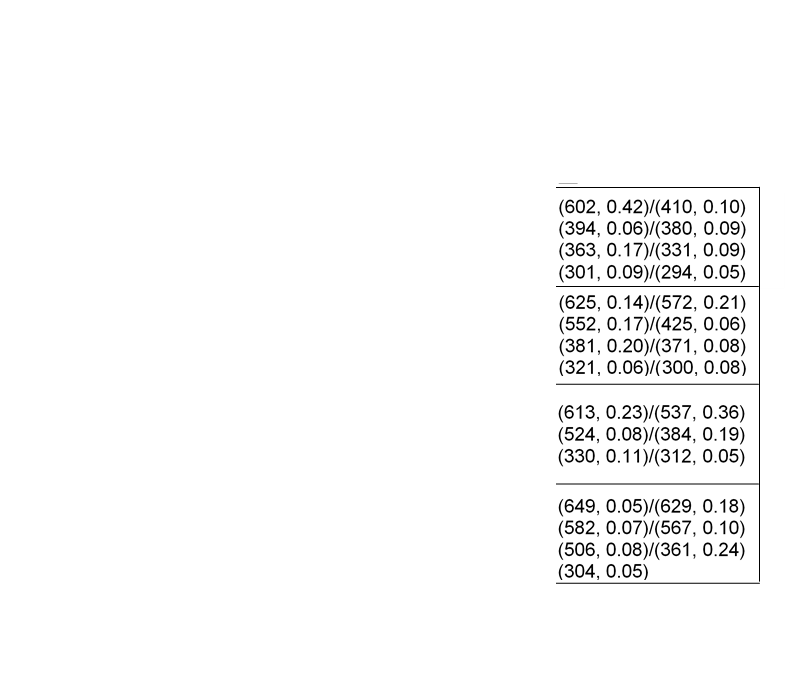


**Table S7.** The values of computed wavelengths corresponding with the computed spectra in Figure 5.

a)


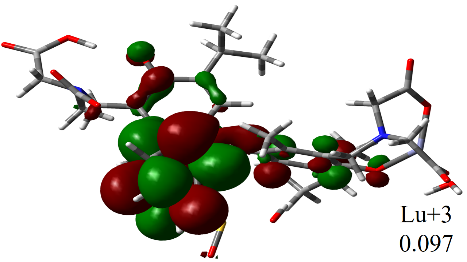

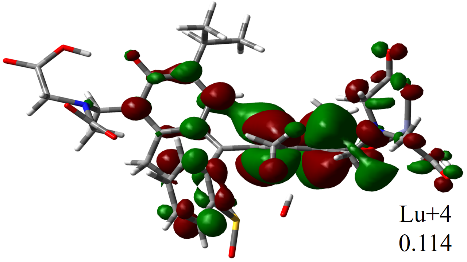

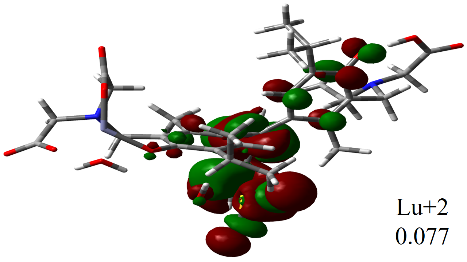


b)


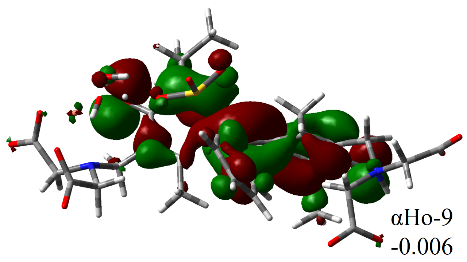

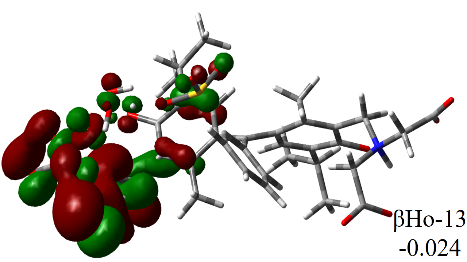

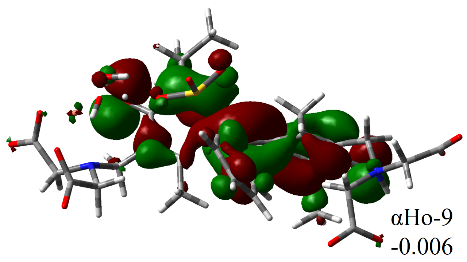

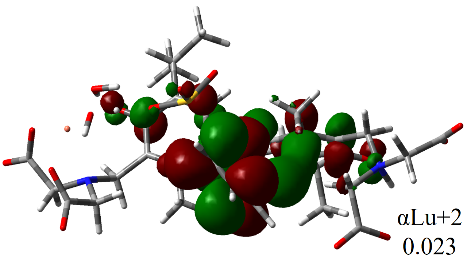

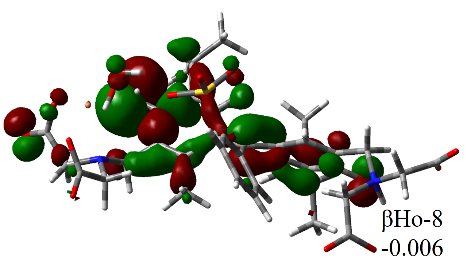

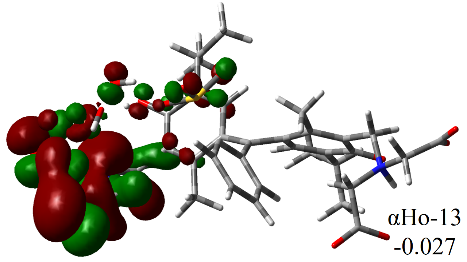

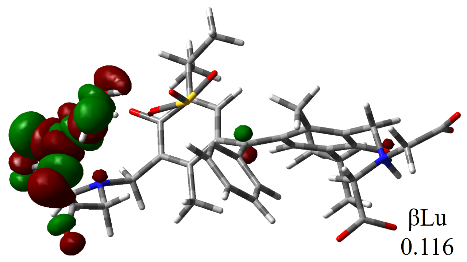

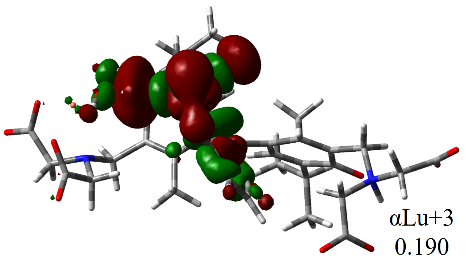

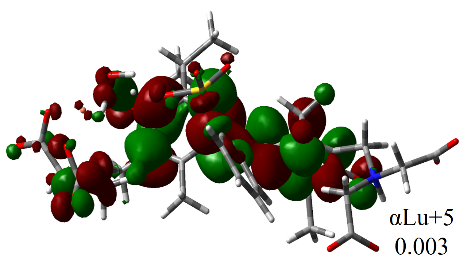

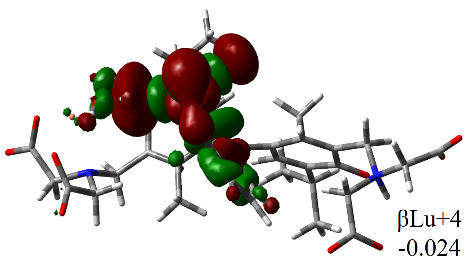

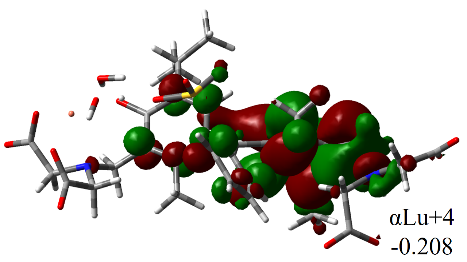


c)


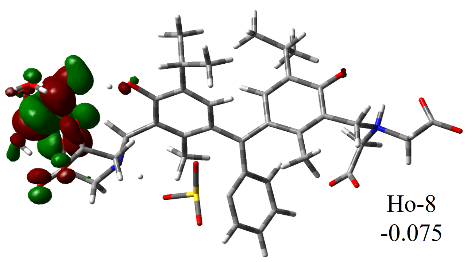

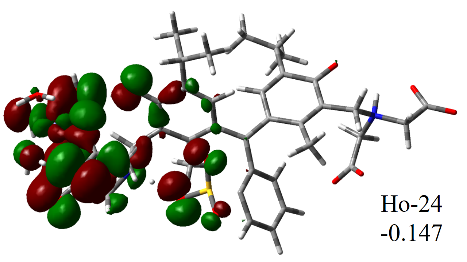

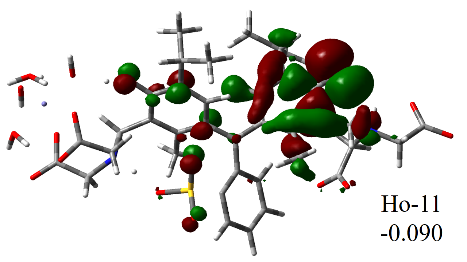

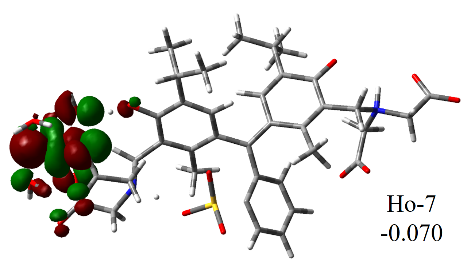

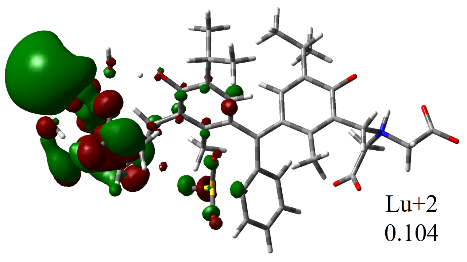

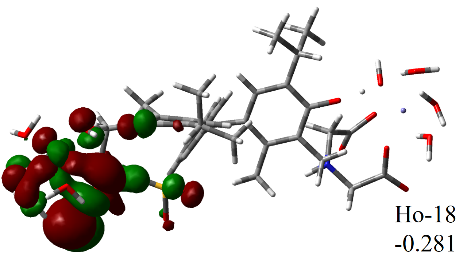

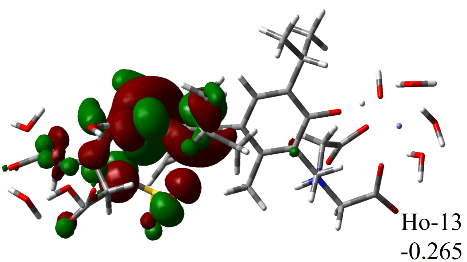

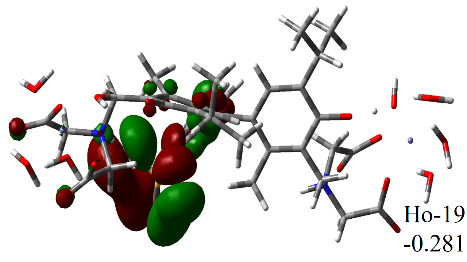


d)

**Figure S7.** Representation of MOs involved in the major electronic transitions for a) Zn(MTB), b) Cu(MTB), c) Fe(MTB) and d) Fe_2_(MTB) complexes, respectively.

**Figure S8.** HMSs used in the computations for the Fe_2_(MTB) complexes.

**Cont. Figure S8.** HMSs used in the computations for the Fe_2_(MTB) complexes.

**Table S8.** Bond lengths (Å), angles (°), and preferred geometries for the optimized complexes of Fe_2_(MTB) with Oh geometry in HMSs 1 to 22 in Figure S8. The atom names are indicated in Figure S6. The number in parentheses indicates the number of water molecules involved in the coordination.

| HMS | Metal ion | Bond lengths (Å) | | | | | | | | Angles (°) | | | | | | | | | | | | | Pref. geometry |
| --- | --- | --- | --- | --- | --- | --- | --- | --- | --- | --- | --- | --- | --- | --- | --- | --- | --- | --- | --- | --- | --- | --- | --- |
|  |  | MI-Ohy | | | | MI-Oca_1_ | | | | Ohy-MI-Oca_1_ | | | | | | | | | | | | |  |
| 1(4) | Fe^II^(1) | 1.98 | | | | 2.02 | | | | 106.14 | | | | | | | | | | | | | Oh |
|  | Fe^II^(2) | 2.10 | | | | 1.99 | | | | 108.31 | | | | | | | | | | | | | Oh |
| 2(4) | Fe^II^(1) | 1.98 | | | | 2.02 | | | | 115.19 | | | | | | | | | | | | | Oh |
|  | Fe^II^(2) | 2.08 | | | | 2.03 | | | | 106.80 | | | | | | | | | | | | | Oh |
| 3(4) | Fe^II^(1) | 1.99 | | | | 2.03 | | | | 106.10 | | | | | | | | | | | | | Oh |
|  | Fe^II^(2) | 2.09 | | | | 2.01 | | | | 109.34 | | | | | | | | | | | | | Oh |
| 4(4) | Fe^II^(1) | 1.99 | | | | 2.04 | | | | 116.64 | | | | | | | | | | | | | Oh |
|  | Fe^II^(2) | 2.10 | | | | 2.00 | | | | 108.54 | | | | | | | | | | | | | Oh |
|  |  | MI-Ohy | | | | MI-Oca_1_ | | | | Oca_1_-MI-Oca_2_ | | | | | | | | | | | | |  |
| 5(4) | Fe^II^(1) | 2.08 | | | | 1.99 | | | | 82.00 | | | | | | | | | | | | | Oh(4) |
|  | Fe^II^(2) | 2.00 | | | | 1.99 | | | | 90.23 | | | | | | | | | | | | | Oh(4) |
| 6(4) | Fe^II^(1) | 2.00 | | | | 1.99 | | | | 96.12 | | | | | | | | | | | | | Oh(4) |
|  | Fe^II^(2) | 2.00 | | | | 2.00 | | | | 85.04 | | | | | | | | | | | | | Oh(4) |
| 7(4) | Fe^II^(1) | 2.00 | | | | 2.00 | | | | 85.48 | | | | | | | | | | | | | Oh(4) |
|  | Fe^II^(2) | 2.00 | | | | 2.01 | | | | 105.77 | | | | | | | | | | | | | Oh(4) |
| 8(4) | Fe^II^(1) | 2.03 | | | | 1.99 | | | | 88.61 | | | | | | | | | | | | | Oh(4) |
|  | Fe^II^(2) | 1.99 | | | | 2.01 | | | | 90.70 | | | | | | | | | | | | | Oh(4) |
| 9(4) | Fe^II^(1) | 2.00 | | | | 2.00 | | | | 87.95 | | | | | | | | | | | | | Oh(4) |
|  | Fe^II^(2) | 2.01 | | | | 1.99 | | | | 90.88 | | | | | | | | | | | | | Oh(4) |
| 10(4) | Fe^II^(1) | 2.00 | | | | 2.00 | | | | 90.04 | | | | | | | | | | | | | Oh(4) |
|  | Fe^II^(2) | 1.98 | | | | 1.98 | | | | 94.21 | | | | | | | | | | | | | Oh(4) |
| 11(4) | Fe^II^(1) | 1.98 | | | | 1.98 | | | | 90.04 | | | | | | | | | | | | | Oh(4) |
|  | Fe^II^(2) | 1.98 | | | | 1.98 | | | | 94.21 | | | | | | | | | | | | | Oh(4) |
| 12(4) | Fe^II^(1) | 2.00 | | | | 2.01 | | | | 96.64 | | | | | | | | | | | | | Oh(4) |
|  | Fe^II^(2) | 1.98 | | | | 1.98 | | | | 95.86 | | | | | | | | | | | | | Oh(4) |
| 13(4) | Fe^II^(1) | 2.00 | | | | 2.00 | | | | 90.42 | | | | | | | | | | | | | Oh(4) |
|  | Fe^II^(2) | 2.00 | | | | 2.00 | | | | 92.45 | | | | | | | | | | | | | Oh(4) |
|  |  | N-MI | MI-Ohy | | | MI-Oca_1_ | | MI-Oca_2_ | | Ohy-MI-Oca_1_ | | | Ohy-MI-Oca_2_ | | | O_C_a_1_-MI-Oca_2_ | | N-MI-Oca_1_ | | | N-MI-Oca_2_ | |  |
| 14(3) | Fe^II^(1) |  | | 2.07 | | 2.03 | | 2.00 | | 109.31 | | 85.09 | | | 94.51 | |  | | |  | | | Oh(3) |
|  | Fe^II^(2) |  | | 2.04 | | 1.98 | | 2.00 | | 90.55 | | 104.04 | | | 100.77 | |  | | |  | | | Oh(3) |
| 15(3) | Fe^II^(1) | 2.09 | |  | | 1.96 | | 1.98 | |  | |  | | |  | | 83.09 | | | 86.46 | | | SPY(3) |
|  | Fe^II^(2) |  | | 2.04 | | 1.99 | | 1.99 | | 90.85 | | 105.80 | | | 98.92 | |  | | |  | | | Oh(3) |
| 16(3) | Fe^II^(1) |  | | 2.09 | | 2.03 | | 2.00 | | 114.07 | | 87.38 | | | 92.00 | |  | | |  | | | Oh(3) |
|  | Fe^II^(2) |  | | 2.02 | | 1.98 | | 2.04 | | 100.85 | | 108.66 | | | 107.88 | |  | | |  | | | Oh(3) |
| 17(3) | Fe^II^(1) | 2.08 | |  | | 1.97 | | 1.98 | |  | |  | | | 94.17 | | 83.24 | | | 86.75 | | | Oh(3) |
|  | Fe^II^(2) |  | | 2.01 | | 2.00 | | 2.05 | | 100.24 | | 111.90 | | | 106.12 | |  | | |  | | | Oh(3) |
|  |  | MI-Ohy | | | | MI-N | | MI-Oca_2_ | | Ohy-MI-N | | | | Ohy-MI-Oca_2_ | | | | | N-MI-Oca_2_ | | | |  |
| 18(4) | Fe^II^(1) | 1.95 | | | | 2.09 | | 1.95 | | 87.25 | | | |  | | | | |  | | | | Oh(4) |
|  | Fe^II^(2) | 2.01 | | | | 2.01 | | 1.98 | | 93.03 | | | | 173.12 | | | | | 86.26 | | | | Oh(3) |
|  |  | N-MI | | | | MI-Oca_1_ | | MI-Oca_2_ | | N-MI-Oca_1_ | | | | N-MI-Os | | | | | Oca_1_-MI-Oca_2_ | | | |  |
| 19(3) | Fe^II^(1) | 2.10 | | | | 1.99 | | 1.98 | | 85.63 | | | | 81.88 | | | | | 100.59 | | | | Oh(3) |
|  | Fe^II^(2) | 2.12 | | | | 1.97 | | 1.98 | | 81.78 | | | | 85.38 | | | | | 100.76 | | | | Oh(3) |
| 20(3) | Fe^II^(1) | 2.11 | | | | 1.99 | | 1.97 | | 85.40 | | | | 81.65 | | | | | 101.36 | | | | Oh(3) |
|  | Fe^II^(2) | 2.13 | | | | 1.96 | | 1.97 | | 82.55 | | | | 86.24 | | | | | 99.53 | | | | Oh(3) |
|  |  | Ohy-MI | | | | N-MI | | MI-Oca_2_ | | Ohy-MI-Oca_2_ | | | | | | | N-MI-Oca_2_ | | | | | | Oh(3) |
| 21(3) | Fe^II^(1) | 1.92 | | | | 2.00 | | 1.96 | | 95.50 | | | | | | | 89.37 | | | | | | Oh(3) |
|  | Fe^II^(2) | 1.99 | | | | 2.03 | | 1.95 | | 104.96 | | | | | | | 87.24 | | | | | |  |
|  |  | N-MI | | | MI-Ohy | | MI-Oca_1_ | | MI-Oca_2_ | N-MI-Ohy | Ohy-MI-Oca_1_ | | | | Ohy-MI-Oca_2_ | | Oca_1_-MI-Oca_2_ | | | N-MI-Oca_1_ | | N-MI-Oca_2_ |  |
| 22(2) | Fe^II^(1) | 1.98 | | | 1.94 | | 1.94 | | 1.98 | 91.07 | 94.40 | | | | 169.34 | | 96.26 | | | 85.45 | | 89.37 | Oh(2) |
|  | Fe^II^(2) | 1.99 | | | 1.99 | | 2.02 | | 1.95 | 96.77 | 170.50 | | | | 89.87 | | 99.59 | | | 84.68 | | 86.96 | Oh(2) |

**Discussion on the Geometrical Parameters in Table S8.** The Fe^II^ (1)–Oca_1_ (1.94 to 2.08 Å), Fe^II^ (1)–Oca_2_ (1.97 to 2.01 Å), Fe^II^ (1)–Ohy (1.92 to 2.09 Å), Fe^II^ (1)–N (1.98 to 2.11 Å), Fe^II^ (2)–Oca_1_ (1.96 to 2.01 Å), Fe^II^ (2)–Oca_2_ (1.95 to 2.05 Å), Fe^II^ (2)–Ohy (1.99 to 2.04 Å) and Fe^II^ (2)–N (1.99 to 2.13 Å) bond lengths are all within the reported normal ranges for other complexes of Fe^II^ in literature.

**Table S9.** Wavelength (*λ*), excitation energy (*E*), difference with the experimental values ($\Delta$*λ*), and oscillator strengths (*f*) of lowest computed energy transitions for the obtained Fe_2_(MTB) complexes.

| Geometry | No. HMS | Transitions from 350 to 750 nm | | |
| --- | --- | --- | --- | --- |
|  |  | λ/nm (*E*/eV) | $\Delta$*λ/nm(E, eV)* | *f* |
| Oh | Experiment | 622(1.99) |  |  |
|  | 1(4) | 588(2.11) | 34(0.12) | 0.54 |
|  | 2(4) | 560(2.21) | 62(0.22) | 0.14 |
|  | 3(4) | 633(1.96) | 31(0.09) | 0.50 |
|  | 4(4) | 586(2.12) | 36(0.13) | 0.50 |
|  | 5(4) | 482(2.57) | 140(0.58) | 0.34 |
|  | 6(4) | 356(3.48) | 266(1.49) | 0.11 |
|  | 7(4) | 394(3.15) | 228(1.15) | 0.14 |
|  | 8(4) | 429(2.89) | 193(0.90) | 0.13 |
|  | 9(4) | 426(2.91) | 196(0.92) | 0.32 |
|  | 10(4) | 463(2.68) | 159(0.68) | 0.42 |
|  | 11(4) | 463(2.68) | 159(0.68) | 0.42 |
|  | 12(4) | 450(2.76) | 172(0.76) | 0.57 |
|  | **13(4)** | **607(2.04)** | **15(0.05)** | **0.42** |
|  | 14(3) | 572(2.17) | 50(0.17) | 0.43 |
|  | 15(3) | 510(2.43) | 112(0.44) | 0.51 |
|  | **16(3)** | **625(1.98)** | **3(0.01)** | **0.36** |
|  | **17(3)** | **613(2.02)** | **9(0.03)** | **0.23** |
|  | 18(4) | 670(1.85) | 48(0.14) | 0.37 |
|  | 19(3) | 573(2.16) | 49(0.17) | 0.07 |
|  | 20(3) | 430(2.88) | 192(0.89) | 0.25 |
|  | **21(3)** | **649(1.91)** | **27(0.08)** | **0.05** |
|  | 22(2) | 820(1.51) | 198(0.48) | 0.07 |

Oh Geometry

**Figure S9.** HMSs used in the computations for the Zn(MTB)_2_ and Cu(MTB)_2_ complexes.

**Cont. Figure S9.** HMSs used in the computations for the Zn(MTB)_2_ and Cu(MTB)_2_ complexes.

**Cont. Figure S9.** HMSs used in the computations for the Zn(MTB)_2_ and Cu(MTB)_2_ complexes.

SP Geometry

**Cont. Figure S9.** HMSs used in the computations for the Zn(MTB)_2_ and Cu(MTB)_2_ complexes.

**Cont. Figure S9.** HMSs used in the computations for the Zn(MTB)_2_ and Cu(MTB)_2_ complexes.

**Cont. Figure S9.** HMSs used in the computations for the Zn(MTB)_2_ and Cu(MTB)_2_ complexes.

Td Geometry

**Cont. Figure S9.** HMSs used in the computations for the Zn(MTB)_2_ and Cu(MTB)_2_ complexes.

**Cont. Figure S9.** HMSs used in the computations for the Zn(MTB)_2_ and Cu(MTB)_2_ complexes.

**Cont. Figure S9.** HMSs used in the computations for the Zn(MTB)_2_ and Cu(MTB)_2_ complexes.

**Table S10.** Bond lengths (Å), angles (°), and preferred geometries for the optimized complexes of Zn(MTB)_2_ and Cu(MTB)_2_ with Oh, SP, and Td geometries in HMSs 1 to 27, 18 to 26, and 10 to 21 respectively in Figure S9. The number in parentheses indicates the number of water molecules involved in the coordination.

| Metal ion | HMS | Bond lengths (Å) | | | | | | | | | | | | | Angles (°) | | | | | | Pref. geometry |
| --- | --- | --- | --- | --- | --- | --- | --- | --- | --- | --- | --- | --- | --- | --- | --- | --- | --- | --- | --- | --- | --- |
|  |  | MTB_1_ | | MTB_2_ | | | | | | | | | | |  |  |  |  |  |  |  |
|  |  | MI-Oca_1_ | MI-O_Ca2_ | | | | | | | MI-O_Ca1'_ | | | | MI-O_Ca2'_ | Oca_1_-MI-Oca_2_ | Oca_1'_-MI-Oca_2'_ | Oca_1_-MI-Oca_1'_ | Oca_1_-MI-Oca_2'_ | Oca_2_-MI-Oca_1'_ | Oca_2_-MI-Oca_2'_ |  |
| Zn^II^ | 1(2) | - | 2.02 | | | | | | | 2.19 | | | | 2.02 | - | 82.80 | - | - | 172.58 | 100.98 | TBP |
|  | 1(2) | 2.21 | 1.97 | | | | | | | 1.99 | | | | - | 85.44 | - | 90.00 | - | 167.73 | - | SPY |
|  | 2(2) | 2.27 | 1.97 | | | | | | | 2.00 | | | | - | 84.49 | - | 93.25 | - | 175.49 | - | SPY |
|  | 4(2) | 2.18 | 1.98 | | | | | | | 1.99 | | | | - | 89.56 | - | 91.10 | - | 171.88 | - | SPY |
|  | 8(2) | - | 2.05 | | | | | | | 2.07 | | | | 2.02 | - | 81.00 | - | - | 157.56 | 97.00 | SPY |
|  |  | Bond lengths (Å) | | | | | | | | | | | | | Angles (°) | | | | | |  |
|  |  | MTB_1_ | | | | | | | | MTB_2_ | | | | |  |  |  |  |  |  |  |
|  |  | MI-Oca_1_ | MI-O_Ca2_ | | | | | | | | MI-O_Ca2'_ | | | MI-O_Ca2'_ | Oca_1_-MI Oca_2_ | Oca_1'_-MI-Oca_2'_ | Oca_1_-MI-Oca_1'_ | Oca_1_-MI-Oca_2'_ | Oca_2_-MI-Oca_1'_ | Oca_2_-MI-Oca_2'_ |  |
| Zn^II^ | 10(2) | - | 2.00 | | | | | | | | 1.95 | | | - | - | - | - | - | 127.50 | - | Td |
|  |  | Bond lengths (Å) | | | | | | | | | | | | | Angles (°) | | | | | |  |
|  |  | MTB_1_ | | | | | | | | MTB_2_ | | | | |  |  |  |  |  |  |  |
|  |  | MI-Oca_1_ | MI-O_Ca2_ | | | | | | | MI-O_Ca1'_ | | MI-O_Ca2'_ | | MI-N | Oca_1_-MI-Oca_2_ | Oca_1'_-MI-Oca_2'_ | Oca_1'_-MI-N | Oca_2'_-MI-N | Oca_1_-MI-N | Oca_2_-MI-N |  |
| Zn^II^ | 19(1) | 2.13 | 2.14 | | | | | | | 2.11 | | 2.00 | | 2.31 | 95.10 | 156.15 | 79.04 | 80.00 | 95.69 | 166.64 | Oh |
|  |  |  |  | | | | | | |  | |  | |  | Oca_1_-M-Oca_1'_ | Oca_1_-M-Oca_2'_ | Oca_2_-M- Oca_1'_ | Oca_2_-M- Oca_2'_ |  |  |  |
|  |  |  |  | | | | | | |  | |  | |  | 95.65 | 101.64 | 92.82 | 105.44 |  |  |  |
| Cu^II^ | 19(1) | 2.28 | 2.07 | | | | | | | 2.00 | | 1.95 | | 2.10 | 102.54 | 166.13 | 84.31 | 84.31 | 98.35 | 158.77 |  |
|  | 22(1) | 2.20 | 1.99 | | | | | | | 2.03 | | 1.98 | | 2.12 | 103.62 | 162.46 | 82.50 | 83.23 | 10.1.21 | 155.11 |  |
|  |  |  |  | | | | | | |  | |  | |  | Oca_1_-M-Oca_1'_ | Oca_1_-M-Oca_2'_ | Oca_2_-M- Oca_1'_ | Oca_2_-M- Oca_2'_ |  |  |  |
|  |  |  |  | | | | | | |  | |  | |  | 90.44 | 98.66 | 86.38 | 98.36 |  |  | SPY |
|  |  |  |  | | | | | | |  | |  | |  | 95.58 | 96.88 | 93.43 | 95.22 | - | - | SPY |
|  |  | Bond lengths (Å) | | | | | | | | | | | | | Angles (°) | | | | | |  |
|  |  | MTB_1_ | | | | | MTB_2_ | | | | | | | |  |  |  |  |  |  |  |
|  |  | MI-Oca_1_ | | |  | MI-O_Ca1'_ | | | | | | MIO_Ca2'_ | | MI-N | Oca_1_-M-Oca_1'_ | Oca_1_-MI-Oca_2'_ | Oca_1_-MI-N | Oca_1'_-MI-Oca_2'_ | Oca_1'_-MI-N | Oca_2'_-MI-N |  |
| Cu^II^ | 22 | 1.94 | | |  | 1.97 | | | | | | 1.94 | | 2.09 | 96.35 | 97.23 | 174.04 | 165.11 | 93.12 | 83.99 | SP |
|  | 24 | 1.98 | | |  | 1.94 | | | | | | 1.97 | | 2.12 | 97.66 | 100.18 | 166.56 | 154.02 | 84.82 | 82.20 | SP |
|  |  | Bond lengths (Å) | | | | | | | | | | | | | Angles (°) | | | | | |  |
|  |  | MTB_1_ | | | | | | | | | | | | MTB_2_ |  |  |  |  |  |  |  |
|  |  | MI-Oca_1_ | MI-Oca_2_ | | | | | | MI-Ohy | | | |  | M- Oca_1'_ | Oca_1_-MI- Oca_2_ | Oca_1_-MI- Ohy | Oca_2_-MI- Ohy | Oca_1'_-MI- Oca_1_ | Oca_1'_-MI- Oca_2_ | Oca_1'_-MI- Ohy |  |
| Zn^II^ | 10 | 1.96 | 1.98 | | | 2.07 | | | | | |  | | 1.95 | 98.52 | 99.02 | 115.58 | 131.40 | 117.52 | 99.82 | Td |
|  | 11 | 2.02 | 2.04 | | | 2.12 | | | | | |  | | 2.00 | 122.01 | 104.96 | 117.15 | 113.95 | 100.75 | 94.81 | Td |
|  | 12 | 1.98 | 2.02 | | | 2.02 | | | | | |  | | 1.94 | 94.85 | 103.41 | 110.40 | 123.29 | 109.35 | 108.33 | Td |
|  | 13 | 2.03 | 2.06 | | | 2.10 | | | | | |  | | 2.03 | 121.90 | 107.03 | 115.06 | 110.22 | 97.52 | 103.04 | Td |
|  |  | Bond lengths (Å) | | | | | | | | | | | | | Angles (°) | | | | | |  |
|  |  | MTB_1_ | | | | | | | | | | MTB_1_ | | |  |  |  |  |  |  |  |
|  |  | MI-Oca_1_ | MI-Ohy | | | | |  | | | | MI-N | | MI-Oca_1'_ | Oca_1_-MI- Ohy | Oca_1_-MI-N | Ohy-MI-N | Oca_1'_-MI- Oca_1_ | Oca_1’_-MI- Ohy | Oca_1’_-MI-N |  |
| Cu^II^ | 18 | 1.95 | 1.95 | | | | |  | | | | 2.05 | | 1.97 | 175.41 | 84.08 | 91.60 | 91.09 | 93.27 | 174.58 | SP |
|  | 19 | 1.97 | 1.99 | | | | |  | | | | 2.01 | | 1.95 | 171.42 | 82.60 | 88.93 | 93.06 | 95.51 | 170.23 | SP |
|  | 20 | 1.96 | 1.98 | | | | |  | | | | 2.09 | | 1.97 | 172.45 | 89.46 | 89.47 | 91.76 | 95.77 | 170.84 | SP |
|  | 21 | 1.99 | 2.05 | | | | |  | | | | 2.18 | | 1.97 | 165.07 | 85.25 | 80.40 | 92.45 | 101.61 | 172.23 | SP |

**References:**

1. Marquardt, D. W. An algorithm for least-squares estimation of nonlinear parameters. *J. Soc. Ind. Appl. Math.* **11**, 431-441 (1963).

2. Tahmasbi, V. & Noori, S. Application of levenberg-marquardt method for estimation of the thermophysical properties and thermal boundary conditions of decomposing materials. *Heat Transf. Eng.* **41**, 449-475 (2019).

3. Ghasemi, J.; Nayebi, S.; Kubista, M. & Sjogreen, B. A new algorithm for the determination of protolytic constants from spectrophotometric data in multiwavelength mode: calculations of acidity constants of 4-(2-Pyridylazo) resorcinol (PAR) in mixed nonaqueous-water solvents. *Talanta* **68**, 1201-1214 (2006).

4 Alves, L. A.; de Castro, A. H.; de Mendonça, F. G. & de Mesquita, J. P. Characterization of acid functional groups of carbon dots by nonlinear regression data fitting of potentiometric titration curves. *Appl. Surf. Sci.* **370**, 486-495 (2016).

5. Emami, F.; Maeder, M. & Abdollahi, H. Model-based analysis of coupled equilibrium-kinetic processes: indirect kinetic studies of thermodynamic parameters using the dynamic data. *Analyst* **140**, 3121-3135 (2015).

6. Coelho, L. H. G. & Gutz, I. G. R. Trace analysis of acids and bases by conductometric titration with multiparametric non-linear regression. *Talanta* **69**, 204-209 (2006).

7. Szakács, Z. & Hägele, G. Accurate determination of low pK values by 1H NMR titration. *Talanta* ***62***, 819-825 (2004).

8. Zhou, K.; Hou, J.; Fu, H.; Wei, B. & Liu, Y. Estimation of relative permeability curves using an improved levenberg-marquardt method with simultaneous perturbation jacobian approximation. *J. Hydrol.* **544**, 604-612 (2017).

9. Gavin, H. The levenberg-marquardt method for nonlinear least squares curve-fitting problems. *Dep. Civ. Environ. Eng. Duke Univ.* **28**, 1-15 (2011).

10. Press, W. H.; Teukolsky, S. A.; Vetterling, W. T. & Flannery B. P. Numerical recipes in C: the art of scientific computing (Cambridge university press, 1993).

11. Maeder, M. & Neuhold, Y.-M. Practical data analysis in chemistry (Elsevier, 2007).

12. Chaminade, P.; Baillet, A.; Ferrier, D.; Bourguignon, B. & Massart, D. L. Efficient determination of the pK_a_ values of six chlorinated phenols by reversed-phase liquid chromatography. *Anal. Chim. Acta* **280**, 93-101 (1993).

13. Kumar, K. & Mishra, A. K. Multivariate curve resolution alternating least square (MCR-ALS) analysis on total synchronous fluorescence spectroscopy (TSFS) data sets: comparing certain ways of arranging TSFS-based three-way array. *Chemom. Intell. Lab. Syst.* **147**, 66-74 (2015).

14. Smith, J. P.; Holahan, E. C.; Smith, F. C.; Marrero, V. & Booksh, K. S. A novel multivariate curve resolution-alternating least squares (MCR-ALS) methodology for application in hyperspectral raman imaging analysis. *Analyst* **144**, 5425-5438 (2019).

15. Tian, X.; Zhang, G.; Shao, Y. & Yang, Z. Towards enhanced metabolomic data analysis of mass spectrometry image: multivariate curve resolution and machine learning. *Anal. Chim. Acta* **1037**, 211-219 (2018).

16. Aslani, M. A. A.; Kuru, Y. F. & Aslani, C. K. Application of central composite design and rank annihilation factor analysis for optimization of mixed chelate of UO_2_^2+^ Complex with ammonium-N, N-Tetramethylenedithiocarbamate and estimation of Kf Value. *Radiochim. Acta* **104**, 23-31 (2016).

17. Hemmateenejad, B. & Yousefinejad, S. Multivariate standard addition method solved by net analyte signal calculation and rank annihilation factor analysis. *Anal. Bioanal. Chem.* **394**, 1965-1975 (2009).

18. Abdollahi, H. & Golshan, A. Rank annihilation factor analysis method for spectrophotometric study of second-order reaction kinetics. *Anal. Chim. Acta* **693**, 26-34 (2011).

19. Kałka, A. J. & Turek, A. M. Fast decomposition of three-component spectra of fluorescence quenching by white and grey methods of data modeling. *J. Fluoresc.* **28**, 615-632 (2018).

20. Afkhami, A.; Khajavi, F. & Khanmohammadi, H. Spectrophotometric determination of complex formation constants between a new schiff base and some transition metals by rank annihilation factor analysis. *J. Chem. Eng. Data* **54**, 866-870 (2009).

21. Afkhami, A.; Keypour, H.; Khajavi, F. & Rezaeivala, M. Application of rank annihilation factor analysis to the spectrophotometric determination of the formation constants of complexes of a new schiff base and some transition metals in different media. *J. Chem. Eng. Data* **55**, 4725-4731 (2010).

22. Ueno, K. Imamura T. & Cheng, K L. Handbook of organic analytical reagents (CRC Press: Boca Raton, 1992).

23. Balderas-Hernández, P. et al. A. Experimental correlation between the pK_a_ value of sulfonphthaleins with the nature of the substituents Groups. *Spectrochim. Acta-Part A Mol. Biomol. Spectrosc.* **69**, 1235-1245 (2008).

24. Houghton, D. T.; Gydesen, N. W.; Arulsamy, N. & Mehn, M. P. Synthesis and characterization of iron (II) quinaldate complexes. *Inorg. Chem.* **49**, 879-887 (2009).

25. Iqbal, M.; Sirajuddin, M.; Ali, S.; Sohail, M. & Tahir, M. N. O-bridged and paddlewheel copper(II) carboxylates as potent DNAC intercalator: synthesis, physicochemical characterization, electrochemical and DNA binding studies as well as POM analyses. *Inorganica Chim. Acta* **440**, 129-138 (2016).

26. Stewart, C. D. et al. Synthesis, crystal structure and investigation of mononuclear copper(II) and zinc(II) complexes of a new carboxylate rich tripodal ligand and their interaction with carbohydrates in alkaline aqueous solution. *J. Inorg. Biochem.* **149**, 25-38 (2015).

27. Kuppuraj, G.; Dudev, M. & Lim, C. Factors governing metal-ligand distances and coordination geometries of metal complexes. *J. Phys. Chem. B* **113**, 2952-2960 (2009).

28. Kuznetsova, O. V.; Egorochkin, A. N.; Khamaletdinova, N. M. & Domratcheva-Lvova, L. G. Bond lengths in organometallic and coordination complexes: substituent effects. *J. Organomet. Chem.* **745-*746***, 34-41 (2013).

29. El-Khateeb, M.; Abul-Futouh, H.; Görls, H.; Weigand, W. & Almazahreh, L. R. Synthesis, characterization and electrochemical investigations of heterocyclic-selenocarboxylate iron complexes. *Inorganica Chim. Acta* **449**, 14-19 (2016).

30. Chen, X.-J.; Yang, Y.; He, W.-W. & Ma, J.-F. Divalent copper and zinc complex based on a new 4,4′-bis-[(dicarboxymethyl)aminomethyl]biphenyl and n-donor ligands: syntheses, structures and photoluminescence. *Polyhedron* **65**, 141-151 (2013).

31. Halcrow, M. A. Jahn-Teller distortions in transition metal compounds, and their importance in functional molecular and inorganic materials. *Chem. Soc. Rev.* **42**, 1784-1795 (2013).
